# Potent Nrf2-Inducing C6-Isothiocyanate Glucose Derivatives with Dual Antioxidant and Antitumor Activity

**DOI:** 10.3390/antiox15010123

**Published:** 2026-01-18

**Authors:** Luis Alberto Prieto, Nora Khiar-Fernández, Rocío Calderón-Ruiz, Emelyne Giraud, José Manuel Calderón-Montaño, Jesús Lucia-Tamudo, Rafael León, José Antonio Pérez-Simón, Miguel López-Lázaro, Rocío Recio, Elena de la Torre, Victoria Valdivia, Inmaculada Fernández

**Affiliations:** 1 Departamento de Química Orgánica y Farmacéutica, Facultad de Farmacia, Universidad de Sevilla, 41012 Sevilla, Spain; lprieto1@us.es (L.A.P.); emelyne.giraud@orange.fr (E.G.); rrecioj@us.es (R.R.); 2 Departamento de Química Orgánica I, Facultad de Ciencias Químicas, Universidad Complutense de Madrid, 28040 Madrid, Spain; nkhiar@ucm.es; 3 Instituto de Biomedicina de Sevilla (IBiS), Hospital Universitario Virgen del Rocío, Consejo Superior de Investigaciones Ccientíficas (CSIC), Universidad de Sevilla, 41013 Sevilla, Spain; rcalderon-ibis@us.es (R.C.-R.); josea.perez.simon.sspa@juntadeandalucia.es (J.A.P.-S.); elena.torre@juntadeandalucia.es (E.d.l.T.); 4 Departamento de Farmacología, Facultad de Farmacia, Universidad de Sevilla, 41012 Sevilla, Spain; jcalderon@us.es (J.M.C.-M.); mlopezlazaro@us.es (M.L.-L.); 5 Faculty of Chemistry and Pharmacy, Institute of Physical and Theoretical Chemistry, University of Regensburg, 93040 Regensburg, Germany; jesus.lucia.tamudo@univie.ac.at; 6 Instituto de Química Médica, Consejo Superior de Investigaciones Científicas (IQM-CSIC), 28006 Madrid, Spain; rafael.leon@iqm.csic.es; 7 Departamento de Hematología, Hospital Universitario Virgen del Rocío, Universidad de Sevilla, 41013 Sevilla, Spain

**Keywords:** Isothiocyanates (ITCs), Nrf2 activation, *S*-glycosyl derivatives, anticancer activity, antileukemic activity

## Abstract

Isothiocyanates (ITCs) are well-known electrophilic agents with antioxidant and anticancer properties, largely attributed to their ability to activate the Nrf2/ARE pathway. Building on previous work with C1-ITC glycosyl derivatives, we designed and synthesized a new series of S-glycosyl isothiocyanates in which the ITC group was repositioned to the C6 carbon of the glucose scaffold. This structural rearrangement yielded stable and synthetically accessible derivatives with markedly enhanced biological profiles. Several compounds showed potent Nrf2 activation at non-cytotoxic concentrations, with CD values comparable to or exceeding those of natural ITCs. In parallel, the new C6-ITC derivatives displayed significant antiproliferative activity against leukemia and solid tumor cell lines. Among them, the phenylsulfone derivative **13** emerged as a particularly promising dual-action molecule, combining strong Nrf2 induction with low-micromolar cytotoxicity. Molecular docking was used as a hypothesis-generating approach and suggested a possible interaction with the STAT3 SH2 domain, although further studies are needed to validate this target. Overall, these results support glucose-based ITCs as a versatile platform for the development of multifunctional antioxidants with complementary anticancer properties.

## 1. Introduction

Isothiocyanates are electrophilic natural products found predominantly in cruciferous vegetables, where they act as key components in plant defense [1,2,3,4]. Beyond their ecological roles, ITCs such as sulforaphane (SFN) have garnered substantial attention for their capacity to inhibit carcinogenesis through multiple mechanisms, including the modulation of phase II detoxification enzymes, inhibition of histone deacetylases, and disruption of signaling pathways (including STAT3 and NF-κB, among others) [5,6,7,8,9].

Among these, STAT3, a transcription factor frequently overactive in various cancers, is a particularly attractive target due to its role in promoting cell survival, proliferation, angiogenesis, and immune evasion [10,11]. SFN and related ITCs are reported to potentially disrupt STAT3 signaling, including proposals of SH2-domain engagement and reduced tyrosine phosphorylation in specific models [12,13,14,15]. Among its structural analogs, iberin (the C3 homolog), 6-methylsulfinylhexyl isothiocyanate (6-HITC, C6) from *Wasabia japonica* (wasabi), and (*R*)-8-methylsulfinyloctyl isothiocyanate (C8) from *Nasturtium officinale* exhibit comparable or enhanced bioactivities, including anti-inflammatory and anticancer effects [16,17,18,19]. In addition to their well-established biological properties, SFN and related ITCs exhibit a certain degree of chemical instability, which has motivated detailed studies on their stabilization, inclusion behavior, and stereochemical discrimination in solution [20].

Nuclear factor erythroid 2–related factor 2 (Nrf2) is a key transcription factor that regulates the cellular antioxidant and cytoprotective response. Under basal conditions, Nrf2 is retained in the cytoplasm by its repressor Kelch-like ECH-associated protein 1 (Keap1) and undergoes proteasomal degradation. Upon oxidative or electrophilic stress, Nrf2 is stabilized and translocates to the nucleus, where it binds to antioxidant response elements (AREs) and induces the expression of phase II detoxifying and antioxidant enzymes, such as HO-1, NQO1, and glutathione S-transferases [21,22,23]. Consequently, pharmacological activation of the Nrf2–ARE pathway has emerged as an attractive strategy for cancer chemoprevention and the modulation of redox-regulated signaling pathways.

The promising activity of SFN and its natural homologues has encouraged the exploration of other sulfur-substituted analogs, which have also yielded positive results [24]. In this context, *S*-glycosides represent a particularly relevant yet still underexplored class of compounds. Naturally occurring *S*-glycosides, such as glucosinolates, are well established as stable precursors of bioactive isothiocyanates and play a central role in the activation of the Nrf2/ARE pathway following enzymatic or microbial hydrolysis [1,2,3,4]. In parallel, several families of synthetic *S*-glycosides have been reported to exhibit cancer-preventive and cytoprotective activities [21,25,26,27,28] frequently associated with the induction of phase II detoxifying enzymes and activation of the Nrf2 signaling pathway [21,27,28].

Notably, thioglycoside conjugates of sulfur-rich heterocycles, including 1,2-dithiole-3-thiones and 1,2-dithiol-3-ones, have shown pronounced cancer-preventive activity in vitro and in vivo, together with the ability to activate the Nrf2–ARE axis and to inhibit pro-tumorigenic transcription factors such as AP-1. These studies highlight *S*-glycosylation as an effective strategy to modulate the stability, bioavailability, and biological profile of electrophilic sulfur-containing scaffolds involved in redox-regulated pathways [27,28].

Despite these advances, the systematic exploration of synthetic *S*-glycosides as modulators of the Nrf2 pathway remains limited, leaving significant room for the development of novel *S*-glycosylated architectures with optimized biological activity.

**Figure 1:**
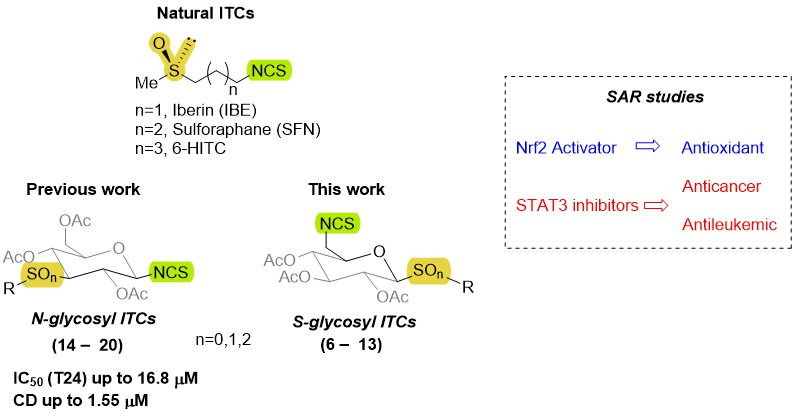
Natural ITCs and the designed *N*- and *S*-Glycosyl ITC derivatives for SAR studies. The previous work focused on *N*-glycosyl ITCs (**14–20**), while the current study focuses on the novel *S*-glycosyl ITC derivatives (**6–13**).

In recent work, we have explored sugar-based analogs of iberin to enhance pharmacokinetic behavior and target selectivity. For this purpose, we synthesized a series of carbohydrate-derived ITCs by functionalizing the anomeric position of glucose with diverse sulfur-containing groups at position 3 (*N*-glycosyl ITCs, **14**–**20**, Figure 1) [29]. These compounds exhibited significant cytotoxic activity against solid tumor cell lines, particularly bladder cancer (IC_50,_ T24 up to 16.8 μM), and activated the Nrf2 pathway at subtoxic concentrations (CD up to 1.55 μM). Molecular docking studies suggested a possible interaction with STAT3 via the SH2 domain, which, in spite of being based exclusively on in silico modeling and requiring experimental validation, prompted further exploratory analysis in the present study. Thus, we decided to extend our study to hematological malignancies, where the pathogenic role of STAT3 overactivation is well established. The results of this extended study are presented in this work.

To expand on these findings, we hypothesized that altering the topological arrangement of the ITC and sulfur groups might influence bioactivity. Specifically, building on our previous *N*-glycosyl ITC derivatives bearing the isothiocyanate group at the anomeric position, we synthesized a structurally distinct series in which the ITC moiety was relocated to the primary hydroxyl at C6, while the sulfur substituent was moved to the anomeric position (*S*-glycosyl ITCs, **6**–**13**, Figure 1). Given the known influence of the anomeric effect on electronic properties [30] and, as consequence, on binding affinity, we anticipated this rearrangement could impact both stability and biological interactions. Moreover, while varying sulfur oxidation state is a natural SAR variable in sulfur-containing scaffolds, it is particularly relevant here because the sulfur substituent is placed at the anomeric center, where the stereoelectronic environment of the glycosidic position may amplify oxidation-state–dependent effects (thioether, sulfoxide, and sulfone). A further consideration is that peracetylated sugar derivatives may undergo partial or complete esterase-mediated deacetylation in biological environments (and potentially in cell-based assays), which could affect cellular uptake, stability, and the apparent biological activity. However, the corresponding deacetylated analogs were not prepared or evaluated in this work, as this belongs to metabolism-oriented and protection-pattern optimization studies.

This study aims to systematically assess the structure–activity relationships (SAR) of these C6-ITC derivatives through biological and computational methods. We evaluated their cytotoxic effects on leukemia and solid tumor cell lines and their ability to activate the antioxidant Nrf2 pathway. In parallel, a hypothesis-generating molecular docking analysis was performed to explore potential binding modes with STAT3 as a putative target; experimental validation of STAT3 engagement was beyond the scope of the present study.

We have evaluated their docking behavior with STAT3 as a potential therapeutic target, their cytotoxic effects on leukemia and solid tumor cell lines, and their ability to activate the antioxidant Nrf2 pathway. Together, these findings contribute to the rational design of sugar-based ITCs as dual-function anticancer agents.

## 2. Materials and Methods

### 2.1. Experimental Procedures and Analytical Techniques

General experimental procedures and analytical techniques are provided in the Supporting Information and follow the conditions previously reported by our group [29]. The natural ITCs used as references, iberverine, racemic iberin, and cheiroline, have been prepared as previously described [29].

### 2.2. Chemical Synthesis

**Methyl2,3,4-tri-*O*-acetyl-6-azido-1,6-dideoxy-1-thio-β-D-glucopyranoside, 2**

Under an argon atmosphere and at room temperature, trimethyl(methylthio)silane (0.19 mL, 1.35 mmol) is added to a solution of **1** (200 mg, 0.54 mmol) in CH_2_Cl_2_ (2 mL). Subsequently, boron trifluoride etherate (0.38 mL, 3.24 mmol) is added dropwise. The reaction is kept stirring at 40 °C for 20 h. After this time, the mixture is treated with saturated NaHCO_3_ solution (15 mL). The aqueous phase is extracted with CH_2_Cl_2_ (3 × 25 mL) and the combined organic phases are washed with saturated NaCl solution (1 × 25 mL) dried over anhydrous Na_2_SO_4_, and the solvent is evaporated under reduced pressure. The crude is purified by silica gel column chromatography (hexane/AcOEt 5:1) to obtain compound **2** (31 mg, 0.09 mmol) as a brown syrup.

Yield: 16%. ^1^H-NMR 500 MHz, CDCl_3_: δ 5.22 (t, *J* = 9.4 Hz, 1H, H3), 5.06 (t, *J* = 9.7 Hz, 1H, H2), 5.02 (t, *J* = 9.7 Hz, 1H, H4), 4.42 (d, *J* = 10.0 Hz, 1H, H1), 3.72 (ddd, *J* = 9.9, 5.8 and 3.1 Hz, 1H, H5), 3.34 (dd, *J* = 13.5 and 3.1 Hz, 1H, H6), 3.30 (dd, *J* = 13.5 and 5.8 Hz, 1H, H6′), 2.18 (s, 3H, -SCH_3_), 2.06 (s, 3H, CH_3_COO-), 2.02 (s, 3H, CH_3_COO-), 2.00 (s, 3H, CH_3_COO-) ppm. ^13^C-NMR 125 MHz, CDCl_3_: δ 170.3, 169.6, 169.5, 83.0, 77.6, 73.8, 69.5, 69.1, 51.2, 20, 8, 20.7 (2), 11.2 ppm. HRMS: Calculated for C_13_H_19_O_7_N_3_NaS [M + Na]^+^: 384.0847; found 384.0844 (−0.7 ppm).

#### 2.2.1. General Procedure for the Synthesis of Isothiocyanates

To a solution of the corresponding azide (100 mol%) in Et_2_O, PPh_3_ (250 mol%) is added at room temperature, and the mixture is stirred at reflux for 30 min. Once the starting product is consumed (controlled by TLC), the solvent is evaporated under reduced pressure, and the crude is dissolved in CS_2_. Subsequently, the mixture is heated in the microwave at 150 °C for 15 min. The crude is purified by silica gel column chromatography.

**Methyl 2,3,4-tri-*O*-acetyl-6-isothiocyanato-1,6-dideoxy-1-thio-β-D-glucopyranoside, 6**

It is synthesized following the general procedure starting from azide **2** (76 mg, 0.21 mmol), Et_2_O (11 mL), PPh_3_ (143 g, 0.53 mmol), and CS_2_ (5 mL). The crude is purified by silica gel column chromatography (AcOEt/hexane 1:5) to obtain isothiocyanate **6** (39 mg, 0.10 mmol) as a brown syrup.

Yield: 49%. ^1^H-NMR 500 MHz, CDCl_3_: δ 5.24 (t, *J* = 9.4 Hz, 1H, H3), 5.05 (t, *J* = 9.7 Hz, 1H, H2), 4.98 (t, *J* = 9.6 Hz, 1H, H4), 4.45 (d, *J* = 10.0 Hz, 1H, H1), 3.72 (ddd, *J* = 9.8, 5.9 and 3.3 Hz, 1H, H5), 3.68 (dd, *J* = 14.8 and 3.2 Hz, 1H, H6), 3.61 (dd, *J* = 14.9 and 5.9 Hz, 1H, H6′), 2.22 (s, 3H, -SCH_3_), 2.06 (s, 3H, CH_3_COO-), 2.05 (s, 3H, CH_3_COO-), 2.01 (s, 3H, CH_3_COO-) ppm. ^13^C-NMR 125 MHz, CDCl_3_: δ 170.2, 169.6, 169.5, 83.2, 76.2, 73.5, 69.7, 69.1, 46.3, 20.8, 20.7, 11.7 ppm. HRMS: Calculated for C_14_H_19_O_7_NNaS_2_ [M + Na]^+^: 400.0495; found 400.0482 (−3.3 ppm).

**Ethyl 2,3,4-tri-*O*-acetyl-6-isothiocyanato-1,6-dideoxy-1-thio-β-D-glucopyranoside, 7**

It is synthesized following the general procedure starting from azide **3** (107 mg, 0.29 mmol), Et_2_O (15 mL), PPh_3_ (189 g, 0.72 mmol), and CS_2_ (15 mL). The crude is purified by silica gel column chromatography (AcOEt/hexane 1:5) to obtain isothiocyanate **7** (99 mg, 0.25 mmol) as a brown syrup.

Yield: 88%. ^1^H-NMR 500 MHz, CDCl_3_: δ 5.25 (t, *J* = 9.4 Hz, 1H, H3), 5.04 (t, *J* = 6.5 Hz, 1H, H2), 4.97 (t, *J* = 9.6 Hz, 1H, H4), 4.57 (d, *J* = 10.1 Hz, 1H, H1), 3.74–3.68 (m, 1H, H5), 3.65–3.64 (m, 2H, H6 and H6′), 2.85–2.71 (m, 2H, -SCH_2_CH_3_), 2.07 (s, 6H, CH_3_COO-), 2.02 (s, 3H, CH_3_COO-), 1.30 (t, *J* = 7.5 Hz, 3H, -SCH_2_CH_3_) ppm. ^13^C-NMR 125 MHz, CDCl_3_: δ 170.3, 169.7, 169.5, 83.5, 76.3, 73.6, 70.0, 69.9, 46.4, 24.3, 20.8, 20.7, 20.7 (2), 15.0 ppm. HRMS: Calculated for C_15_H_21_O_7_NNaS_2_ [M + Na]^+^: 4014.0652; found 4014.0645 (−1.7 ppm).

**Phenyl 2,3,4-tri-*O*-acetyl-6-isothiocyanato-1,6-dideoxy-1-thio-β-D-glucopyranoside, 8**

It is synthesized following the general procedure starting from azide **4** (150 mg, 0.35 mmol), Et_2_O (10 mL), PPh_3_ (232 g, 0.88 mmol), and CS_2_ (5 mL). The crude is purified by silica gel column chromatography (AcOEt/hexane 1:3) to obtain isothiocyanate **8** (122 mg, 0.28 mmol) as a syrup.

Yield: 79%. ^1^H-NMR 500 MHz, CDCl_3_: δ 7.54–7.52 (m, 2H, -SC_6_H_5_), 7.39–7.34 (m, 3H, -SC_6_H_5_), 5.23 (t, *J* = 9.4 Hz, 1H, H3), 4.95 (dd, *J* = 10.0 and 9.4 Hz, 1H, H2), 4.92 (t, *J* = 9.3 Hz, 1H, H4), 4.73 (d, *J* = 10.1 Hz, 1H, H1), 3.72–3.66 (m, 3H, H5, H6 and H6′), 2.09 (s, 3H, CH_3_COO-), 2.05 (s, 3H, CH_3_COO-), 1.99 (s, 3H, CH_3_COO-) ppm. ^13^C-NMR 125 MHz, CDCl_3_: δ 170.2, 169.6, 169.3, 133.6, 131.2, 129.4, 128.9, 86.2, 76.0, 73, 7, 69.9, 69.6, 46.2, 20.9, 20.7 (2) ppm. HRMS: Calculated for C_19_H_21_O_7_NNaS_2_ [M + Na]^+^: 462.0652; found 462.0653 (0.2 ppm).

**Phenyl 2,3,4-tri-*O*-acetyl-6-isothiocyanato-1,6-dideoxy-1-thio-α-D-glucopyranoside, 9**

It is synthesized following the general procedure starting from azide **5** (41 mg, 0.10 mmol), Et_2_O (5 mL), PPh_3_ (63 mg, 0.24 mmol), and CS_2_ (5 mL). The crude is purified by silica gel column chromatography (AcOEt/hexane 1:5) to obtain isothiocyanate **9** (19 mg, 0.04 mmol) as a syrup.

Yield: 45%. ^1^H-NMR 500 MHz, CDCl_3_: δ 7.45–7.44 (m, 2H, -SC_6_H_5_), 7.36–7.29 (m, 3H, -SC_6_H_5_), 5.09 (d, *J* = 5.8 Hz, 1H, H1), 5.42 (dd, *J* = 10.2 and 9.3 Hz, 1H, H3), 5.07 (dd, *J* = 10.4 and 5.7 Hz, 1H, H2), 4.97 (dd, *J* = 10.0 and 9.4 Hz, 1H, H4), 4.53–4.45 (m, 1H, H5), 3.65 (d, *J* = 4.6 Hz, 1H, H6 and H6′), 2.11 (s, 3H, CH_3_COO-), 2.08 (s, 3H, CH_3_COO-), 2.04 (s, 3H, CH_3_COO-) ppm. ^13^C-NMR 125 MHz, CDCl_3_: δ 170.0 (2), 169.8, 132.3, 132.1, 129.6, 128.2, 85.5, 70.7, 70.2, 70.0, 68.5, 45.9, 20.9, 20.8 (2) ppm. HRMS: Calculated for C_19_H_21_O_7_NNaS_2_ [M + Na]^+^: 462.0652; found 462.0635 (−3.7 ppm).

#### 2.2.2. General Procedure for the Oxidation of Thioethers to Sulfoxides

To a solution of the corresponding thioglycoside (100 mol%) in CH_2_Cl_2_ under an argon atmosphere, a solution of *m*-CPBA (110 mmol%) in CHCl_3_ is added at −78 °C. After the starting product is completely consumed, the reaction is quenched with saturated NaHCO_3_ solution (40 mL) and extracted with CH_2_Cl_2_ (3 × 40 mL). The combined organic phases are washed with saturated NaCl solution (30 mL) and dried over anhydrous Na_2_SO_4_. Finally, the solvent is evaporated under reduced pressure. The crude is purified by silica gel column chromatography.

**2,3,4-tri-*O*-acetyl-6-isothiocyanato-1,6-dideoxy-1[ethylsulfinyl]-β-D-glucopyranose, 10**

It is synthesized following the general procedure starting from thioglycoside **7** (65 mg, 0.17 mmol), 5 mL of CH_2_Cl_2_, and *m*-CPBA (44 g, 0.19 mmol) in 2 mL of CHCl_3_. After stirring for 30 min, the starting product is completely consumed. The crude contains a mixture of both diastereoisomers in a 0.5:1 *R*_S_:*S*_S_ ratio. After separation by silica gel column chromatography (toluene/tert-butyl methyl ether/MeOH 5:5:0.1), the enantiomer **(*****R*****)-10** (13 mg, 0.03 mmol) is obtained as a colorless syrup and the anomer **(*****S*****)-10** (53 mg, 0.13 mmol) as a yellow syrup, with an overall yield of 94% (18% ***R*** and 76% ***S***).

**2,3,4-tri-*O*-acetyl-6-isothiocyanato-1,6-dideoxy-1[(*S*)-ethylsulfinyl]-β-D-glucopyranose, (*S*)-10**

Yield: 76%. ^1^H-NMR 500 MHz, CDCl_3_: δ 5.32 (t, *J* = 9.2 Hz, 1H, H3), 5.25 (t, *J* = 9.6 Hz, 1H, H2), 5.03 (t, *J* = 9.4 Hz, 1H, H4), 4.39 (d, *J* = 9.9 Hz, 1H, H1), 3.82–3.78 (m, 2H, H5 and H6), 3.63–3.58 (m, 1H, H6′), 3.05–2.91 (m, 2H, -SCH_2_CH_3_), 2.07 (s, 6H, CH_3_COO-), 2.04 (s, 3H, CH_3_COO-), 1.41 (t, *J* = 7.5 Hz, 3H, -SCH_2_CH_3_) ppm. ^13^C-NMR 125 MHz, CDCl_3_: δ 170.0, 169.7, 169.5, 89.8, 76.7, 72.9, 69.0, 68.4, 45.9, 41, 6, 20.7 (3), 6.8 ppm. HRMS: Calculated for C_15_H_21_O_8_NNaS_2_ [M + Na]^+^: 430.0601; found 430.0595 (−1.4 ppm).

**2,3,4-tri-*O*-acetyl-6-isothiocyanato-1,6-dideoxy-1[(*R*)-ethylsulfinyl]-β-D-glucopyranose, (*R*)-10**

Yield: 18%. ^1^H-NMR 500 MHz, CDCl_3_: δ 5.44 (t, *J* = 9.6 Hz, 1H, H3), 5.38 (t, *J* = 9.2 Hz, 1H, H2), 4.98–4.92 (m, 1H, H4), 4.29 (d, *J* = 9.9 Hz, 1H, H1), 3.86–3.79 (m, 2H, H5 and H6), 3.55–3.49 (m, 1H, H6′), 3.26–2.87 (m, 2H, -SCH_2_CH_3_), 2.06 (s, 6H, CH_3_COO-), 2.02 (s, 3H, CH_3_COO-), 1.36 (t, *J* = 7.6 Hz, 3H, -SCH_2_CH_3_) ppm. ^13^C-NMR 125 MHz, CDCl_3_: δ 170.4, 169.5, 168.9, 135.6 (-NCS), 86.6, 77.7, 73.4, 69.4, 66, 9, 46.5, 41.7, 20.7, 20.6 (2), 7.6 ppm. HRMS: Calculated for C_15_H_21_O_8_NNaS_2_ [M + Na]^+^: 430.0601; found 430.0601 (−1.8 ppm).

**2,3,4-tri-*O*-acetyl-6-isothiocyanato-1,6-dideoxy-1[phenylsulfinyl]-β-D-glucopyranose, 11**

It is synthesized following the general procedure starting from **8** (80 mg, 0.18 mmol), 10 mL of CH_2_Cl_2_, and *m*-CPBA (47 g, 0.20 mmol) in 2 mL of CHCl_3_. After stirring for 1 h, the starting product is completely consumed. The crude contains a mixture of both stereoisomers in a ratio of 1:0.5 *R*_S_:*S*_S_. After separation by silica gel column chromatography (hexane/tert-butyl methyl ether/CH_2_Cl_2_ 5:5:0.1), the anomer **(*****R*****)-11**(26 mg, 0.06 mmol) is obtained as a colorless syrup and the anomer **(*****S*****)-11** (31 mg, 0.07 mmol) as a colorless syrup, with an overall yield of 72% (33% ***R*** and 39% ***S***).

**2,3,4-tri-*O*-acetyl-6-isothiocyanato-1,6-dideoxy-1[(*S*)-phenylsulfinyl]-β-D-glucopyranose, (*S*)-11**

Yield: 33%. ^1^H-NMR 500 MHz, CDCl_3_: δ 7.74–7.66 (m, 2H, -SC_6_H_5_), 7.60–7.51 (m, 3H, -SC_6_H_5_), 5.28–5, 23 (m, 2H, H2 and H4), 4.89–4.85 (m, 1H, H3), 4.89–4.85 (m, 1H, H1), 3.71–3.58 (m, 3H, H5, H6 and H6′), 2.03 (s, 3H, CH_3_COO-), 1.99 (s, 3H, CH_3_COO-), 1.90 (s, 3H, CH_3_COO-) ppm. ^13^C-NMR 125 MHz, CDCl_3_: δ 170.2, 169.4 (2), 138.7, 132.2, 129.3 (2), 125.7 (2), 92.4, 76.3, 73.3, 68.8, 67.5, 45.8, 20.7 (3) ppm. HRMS: Calculated for C_19_H_21_O_8_NNaS_2_ [M + Na]^+^: 478.0601; found 478.0594 (−1.3 ppm).

**2,3,4-tri-*O*-acetyl-6-isothiocyanato-1,6-dideoxy-1[(*R*)-phenylsulfinyl]-β-D-glucopyranose, (*R*)-11**

Yield: 39%. ^1^H-NMR 500 MHz, CDCl_3_: δ7.71–7.66 (m, 2H, -SC_6_H_5_), 7.61–7.52 (m, 3H, -SC_6_H_5_), 5.32 (t, *J* = 9.3 Hz, 1H, H3), 5.28 (t, *J* = 9.0 Hz, 1H, H2), 4.89 (t, *J* = 9.3 Hz, 1H, H4), 4.32 (d, *J* = 9.5 Hz, 1H, H1), 3.66–3.56 (m, 3H, H5, H6 and H6′), 2.09 (s, 3H, CH_3_COO-), 2.03 (s, 3H, CH_3_COO-), 2.00 (s, 3H, CH_3_COO-) ppm. ^13^C-NMR 125 MHz, CDCl_3_: δ 170.4, 169.4, 169.1, 138.3, 132.3, 129.5, 125.6, 89.9, 76.7, 73, 4, 69.1, 67.6, 46.0, 20.8, 20.7 (2) ppm. HRMS: Calculated for C_19_H_21_O_8_NNaS_2_ [M + Na]^+^: 478.0601; found 478.0593 (−1.7 ppm).

#### 2.2.3. General Procedure for the Oxidation of Thioethers to Sulfones

To a solution of the corresponding thioglycoside (100 mol%) in CH_2_Cl_2_, at room temperature, a solution of *m*-CPBA (210–310 mol%) in CHCl_3_ is added. After the starting product is completely consumed, the reaction is quenched with saturated NaHCO_3_ solution and extracted with CH_2_Cl_2_ (3 × 25 mL). The combined organic fractions are washed with saturated NaCl solution (1 × 25 mL), dried over anhydride Na_2_SO_4_, and the solvent is evaporated under reduced pressure. The crude is purified by silica gel column chromatography.

**2,3,4-tri-*O*-acetyl-6-isothiocyanato-1,6-dideoxy-1-ethylsulfonyl-β-D-glucopyranose, 12**

It is synthesized following the general procedure starting from thioglycoside **7** (67 mg, 0.17 mmol) in CH_2_Cl_2_ (10 mL) and *m*-CPBA (87 mg, 0.36 mmol) in CHCl_3_ (2 mL). The crude is purified by silica gel column chromatography (AcOEt/hexane 3:1) to obtain compound **12** (20 mg, 0.05 mmol) as a syrup.

Yield: 29%. ^1^H-NMR 500 MHz, CDCl_3_: 5.51 (t, *J* = 9.6 Hz, 1H, H3), 5.35 (t, *J* = 9.3 Hz, 1H, H2), 5.01 (t, *J* = 9.7 Hz, 1H, H4), 4.54 (d, *J* = 10.0 Hz, 1H, H1), 3.85–3.81 (m, 1H, H5), 3.70 (s, 1H, H6), 3.69 (s, 1H, H6′), 3.32–3.16 (m, 2H, -SCH_2_CH_3_), 2.07 (s, 3H, CH_3_COO-), 2.06 (s, 3H, CH_3_COO-), 2.04 (s, 3H, CH_3_COO-), 1.43 (t, *J* = 7.5 Hz, 3H, -SCH_2_CH_3_) ppm. ^13^C-NMR 125 MHz, CDCl_3_: δ 170.1, 169.4 (2), 87.0, 76.8, 72.8, 68.8, 66.4, 46.0, 44.4, 20.7, 20.6 (2), 5.8 ppm. HRMS: Calculated for C_15_H_21_O_9_NNaS_2_ [M + Na]^+^: 446.0550; found 446.0539 (−2.5 ppm).

**2,3,4-tri-*O*-acetyl-6-isothiocyanato-1,6-dideoxy-1-phenylsulfonyl-β-D-glucopyranose, 13**

It is synthesized following the general procedure starting from thioglycoside **8** (80 mg, 0.18 mmol) in CH_2_Cl_2_ (10 mL) and *m*-CPBA (89 mg, 0.38 mmol) in CHCl_3_ (2 mL). The crude is purified by silica gel column chromatography (AcOEt/hexane 7:1) to obtain compound **13** (30 mg, 0.06 mmol) as a yellow syrup.

Yield: 36%. ^1^H-NMR 500 MHz, CDCl_3_: δ 7.95–7.93 (m, 2H, -SC_6_H_5_), 7.76–7.72 (m, 1H, -SC_6_H_5_), 7.66–7.63 (m, 2H, -SC_6_H_5_), 5.27–5.20 (m, 2H, H2 and H3), 4.83 (t, *J* = 9.5 Hz, 1H, H4), 4.55 (d, *J* = 9.6 Hz, 1H, H1), 3.76 (dd, *J* = 14.8 and 3.1 Hz, 1H, H6), 3.67 (ddd, *J* = 9.8, 5.1 and 3.1 Hz, 1H, H5), 3.58 (dd, *J* = 14.8 and 5.1 Hz, 1H, H6′), 2.13 (s, 3H, CH_3_COO-), 2.02 (s, 3H, CH_3_COO-), 1.99 (s, 3H, CH_3_COO-) ppm. ^13^C-NMR 125 MHz, CDCl_3_: δ 170.1, 169.4, 169.3, 135.2, 135.1, 134.4, 130.5 (2), 129.4 (2), 88.9, 75.9, 73.1, 68.4, 67.1, 45.5, 20.8, 20.6 (2) ppm. HRMS: Calculated for C_19_H_21_O_9_NNaS_2_ [M + Na]^+^: 494.0550; found 494.0543 (−1.4 ppm).

### 2.3. Computational Protocol

Iberin derivatives were first modeled with the Avogadro package [31] and then geometry-optimized with ORCA 5.0.3 [32] using the B3LYP/6-311+G(d) level of theory [33,34,35]. To verify that the optimized structures corresponded to true minima, vibrational frequency analyses were carried out for each compound. Simultaneously, the unphosphorylated STAT3 crystal structure (PDB ID: 6TLC) was retrieved from the Protein Data Bank, and nonpolar hydrogens were added to the model [36]. For blind docking between STAT3 and the ligands, Autodock 4 [37] was employed. A grid of 126 × 126 × 126 points with a spacing of 0.303 Å was defined to encompass the SH2 domain. A smoothing factor of 0.5 Å and a Mehler–Solmajer distance-dependent dielectric constant (parameter −0.1465) were applied [38]. Docking was carried out with the Lamarckian Genetic Algorithm (LGA) [39], assigning Gasteiger charges to the atoms [40]. The docking protocol involved a population of 300 individuals, up to 25 million energy evaluations, 10 independent runs, a limit of 27,000 generations, a mutation rate of 0.02, a crossover rate of 0.80, and the survival of only one individual per generation. The ten resulting conformations were then clustered based on an RMSD cutoff of 0.2 Å.

Selected docking poses were further investigated by classical molecular dynamics (MD) simulations [41,42]. Each protein–ligand complex was solvated in a periodic box with an 18 Å buffer, containing roughly 135,000 water molecules, using tleap from AmberTools 22 [43,44,45]. MD simulations were carried out with AMBER 20, applying FF19SB to the protein, TIP3P to water, and GAFF2 to the ligands [46,47,48,49]. Ligand charges were assigned with the RESP approach, derived from ORCA-optimized electrostatic potentials via Multiwfn [50]. To allow a larger integration step, hydrogen mass repartitioning was also taken into account [51]. The simulation protocol began with 10,000 minimization steps: steepest descent for the first half and Newton–Raphson for the second [52]. Afterward, the system was stripped of solvent, and the protein was truncated to retain only the SH2 domain with cpptraj (AmberTools 22) [43,44,45], assuming *S*-glycosyl ITCs mainly interact within this region. The reduced system was then re-solvated (18 Å buffer, ~25,000 water molecules), neutralized with two chloride ions [46,47,48,49], and subjected to another minimization under the same conditions. Harmonic restraints (5 kcal/mol) were applied to the C_α_ atoms of the protein and to the ligand during this step. Subsequently, the system was gradually heated to 300 K over 500 ps in the NVT ensemble, using a Langevin thermostat (collision frequency 2 ps^−1^), followed by an additional 500 ps of equilibration at constant temperature. Next, five consecutive 1 ns simulations in the NPT ensemble equilibrated the density, progressively reducing ligand restraints by 1 kcal/mol in each run. The production stage consisted of a 1 μs NPT simulation at 1 bar, maintained with the Berendsen barostat (2 ps relaxation time, isotropic scaling). Long-range electrostatics were treated with the particle–mesh Ewald method (1 Å grid), and a 12 Å cutoff with a 10 Å switching function was applied for van der Waals and short-range interactions [53]. SHAKE constrained hydrogen bonds, allowing a 4 fs timestep throughout heating, equilibration, and production [54,55,56]. Snapshots were saved every 0.5 ns across the production run and later used to compute binding free energies with the MM-PBSA method in AmberTools 22 [57].

### 2.4. Biological Activity

#### 2.4.1. Reagents and Cell Lines

Azacitidine and cytarabine were obtained from the Hospital Pharmacy Service of Virgen del Rocío Hospital. HL-60 and U937 cells were acquired from American Type Culture Collection (ATCC), and Jurkat and OPM-2 cells were acquired from Leibniz Institute DSMZ. For U937, Jurkat, and OPM-2 cells, maintenance was performed in Roswell Park Memorial Institute (RPMI) medium, whereas HL-60 cells were cultured in Iscove’s Modified Dulbecco’s Medium (IMDM); both media were supplemented with 10% fetal bovine serum and 1% penicillin/streptomycin. All cell lines were cultured under standard conditions of 37 °C, 5% CO_2_, and saturated humidity.

#### 2.4.2. Cell Viability in Leukemic Cell Lines

Cell viability in leukemic cell lines was determined using the CCK-8 Cell Counting Kit (Dojindo Molecular Technologies, Munich, Germany) by the manufacturer’s instructions. A total of 50,000 cells were seeded per well in 96-well plates and subsequently exposed to increasing concentrations (0–100 μM) of each of the compounds. Following an 18 h incubation at 37 °C in 5% CO_2_, 7 μL of CCK-8 reagent was added to each well, with further incubation for 2 h under identical conditions. Absorbance at 450 nm was recorded using a Multiskan™ GO microplate reader (Thermo Fisher Scientific, Waltham, MA, USA). Cell viability values were normalized against untreated controls (100%) and reported as mean ± standard error of the mean (SEM).

#### 2.4.3. Flow Cytometric Analysis of Cell Death

Cell death was corroborated through flow cytometry utilizing Annexin V and 7-AAD staining. Briefly, 100,000 cells were seeded per well in 96-well plates, treated with increasing concentrations (0–100 μM) of the compounds, and incubated for 18 h at 37 °C with 5% CO_2_. Cells were then harvested and stained using the BD Pharmingen PE-Annexin V Apoptosis Detection Kit I (BD Biosciences, Madrid, Spain) according to the provided protocol. Data acquisition occurred on a FACS Canto II cytometer (BD Biosciences, Madrid, Spain), and subsequent analysis was performed with FlowJo v10 software. Cell viability was calculated as the percentage of Annexin V- 7-AAD negative cells, normalized to 100% of the untreated controls. Results are presented as mean ± standard error of the mean (SEM).

#### 2.4.4. Cell Viability in Cancer Cell Lines

To evaluate cell viability, cancer cell lines (A549, MeWo, and T24) and non-malignant keratinocytes (HaCaT) were cultured in parallel under standard conditions. Cell viability was assessed using the resazurin assay, which quantifies metabolic activity through the reduction of resazurin to resorufin, a product proportional to the number of viable cells. Cells were seeded in 96-well plates, treated with increasing concentrations of the test compounds for 72 h, and then incubated with resazurin prior to spectrophotometric readout. Viability was expressed as a percentage relative to untreated controls, and data are presented as mean ± SEM from at least three independent experiments. The full experimental protocol and assay parameters (cell seeding density, incubation times, and absorbance settings) are provided in the Supporting Information and follow our previously reported procedure [29]**.** Statistical comparisons between HaCaT and T24 at selected concentrations and calculation of selectivity indices (S.I. = IC50 HaCaT/IC50 cancer cell line) were performed as previously described [29].

#### 2.4.5. Antioxidant Activity (Nrf2 Induction Activity)

Nrf2/ARE activation was evaluated using AREc32 cells (MCF7 cells stably transfected with the pGL-8xARE luciferase reporter) by quantifying ARE-driven luminescence, following our previously reported protocol without modification [29]. Briefly, cells were seeded in 96-well white plates (20,000 cells/well) and, after 24 h, treated with the selected compounds at the indicated concentrations (duplicates) for an additional 24 h. Luciferase activity was measured using the Luciferase Assay System (Promega E1500, Promega Biotech Ibérica, Madrid, Spain) on a Clariostar plate reader and normalized to basal conditions. Results are reported as CD values (the concentration required to induce a twofold increase in luciferase activity), calculated from dose–response curves fitted by non-linear regression after log transformation (GraphPad Prism 8.0).

Cell viability under the Nrf2 assay conditions was assessed by MTT in parallel, as previously described [29], and EC50 values were consistently > 30 µM, confirming that Nrf2 activation occurred under non-cytotoxic conditions. Downstream Nrf2 target validation (e.g., NQO1, HO-1) was not performed in this study.

## 3. Results and Discussion

### 3.1. Synthesis of 6-ITC Glucose-Based Derivatives

**Scheme 1:**
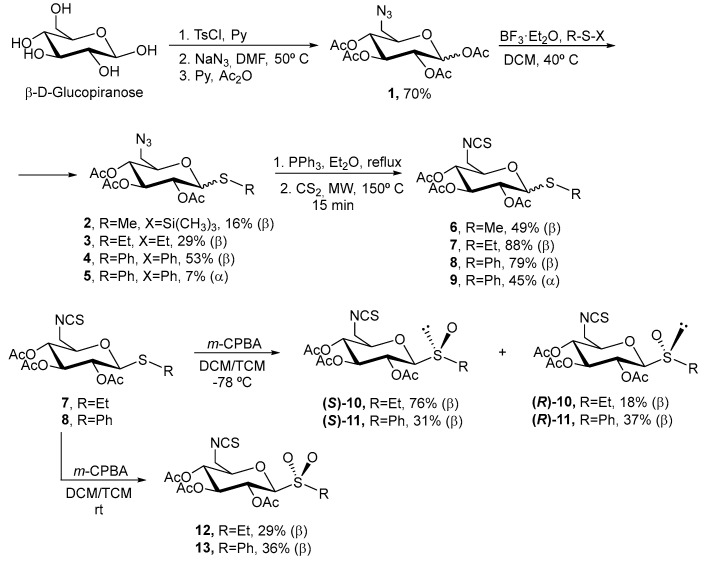
Synthetic route of 6-ITC glucose-based derivatives, **6**–**13**.

The glucose-based ITC derivatives were synthesized using a modular strategy, starting from commercially available β-D-glucopyranose (Scheme 1).

The synthetic route commenced with the regioselective tosylation of the primary alcohol of glucose, followed by nucleophilic substitution with sodium azide to give the C-6 azidoderivative **1** after the peracetylation of the sugar (Scheme 1) [58]. The sulfur function was introduced at the C1 position by thioglycosylation with various thiol nucleophiles under Lewis acid catalysis (BF_3_·Et_2_O) [59,60], to afford a library of thioglycosides (**2**–**5**, Scheme 1) in moderate yields. This step effectively enabled the incorporation of sulfur-containing aglycones with structural diversity. As anticipated from the anchimeric assistance provided by the 2-O-acetyl group during the thioglycosylation step [61,62], the reaction predominantly yielded the β-anomers, which thus became the focus of our study.

The 6-azido-thioglycosides were converted into ITCs via a two-step transformation: first, a Staudinger reduction with triphenylphosphine (PPh_3_), followed by in situ treatment with carbon disulfide (CS_2_) and heating in the microwave (MW) at 150 °C for 15 min, leading to the formation of isothiocyanate derivatives (**6**–**9**, Scheme 1). The process was particularly efficient for ethyl- and phenylthio analogs, **7** and **8**, achieving yields of up to 88%.

Controlled oxidation of thioethers to sulfoxides and sulfones was performed using *m*-chloroperbenzoic acid (*m*-CPBA). Sulfoxides were obtained at low temperatures (−78 °C), enabling access to both (*R*)- and (*S*)-configured isomers (**(*****R*****)** and **(*****S*****)-10** and **(*****R*****)** and **(*****S*****)-11**, Scheme 1) in a 1:2 diastereomeric ratio, whereas sulfones (**12** and **13**, Scheme 1) were formed at room temperature in moderate yields. The configuration at the sulfinyl sulfur of the diastereoisomers was determined following the methodology described by Khiar et al. [24], which relies on the study of proton and carbon NMR spectroscopic patterns. The stereochemical configuration at the sulfinyl sulfur has also been the subject of detailed investigation in sulforaphane analogs, reflecting ongoing interest in understanding how structural variations around the sulfinyl group relate to their chemical and biological behavior [24].

This efficient and concise synthetic approach allows the regioselective installation of the isothiocyanate group at the desired position and enables structural variation at the anomeric center through the incorporation of alkyl or aryl sulfur substituents in various oxidation states. The aqueous solubility and bioavailability of all synthesized isothiocyanates were analyzed using the SwissADME (https://www.swissadme.ch/, accessed on 1 December 2025) tool [63], with the corresponding data reported in the Supplementary Material. Solubility predictions for the ITC derivatives were obtained through the ESOL model [64], while good oral bioavailability was predicted in accordance with Lipinski’s rule-of-five criteria [65].

### 3.2. In Silico Docking to STAT3 SH2 Domain (Computational Studies: Docking, Molecular Dynamics Simulations and Binding Free Energy Predictions)

To explore potential binding modes, a molecular docking study was performed as a hypothesis-generating approach. This computational analysis does not provide experimental evidence of direct target engagement or modulation of STAT3, but it was selected as a putative target based on its frequent dysregulation in cancer and its role in tumor cell proliferation, survival, and immune evasion [66].

The docking protocol was applied to the new *S*-glycoside series (**6**–**13**) and compared with the previously reported *N*-glycoside analogs (**14**–**20**) using the same STAT3 SH2 domain model and workflow [29,67]. The calculations identified three potential binding pockets within the SH2 domain, with pocket 1 consistently providing the most favorable interaction energies for both series (see Figure S7, Supporting Information).

Across the *S*-glycoside series, phenyl-substituted derivatives showed more favorable predicted binding than their ethyl analogs, and increasing sulfur oxidation state (thioether → sulfoxide → sulfone) was generally associated with stronger interactions (Table S2, Supporting Information). In addition, *S*-configured sulfoxides tended to display more favorable binding than their *R*-configured diasteromers.

**Figure 2:**
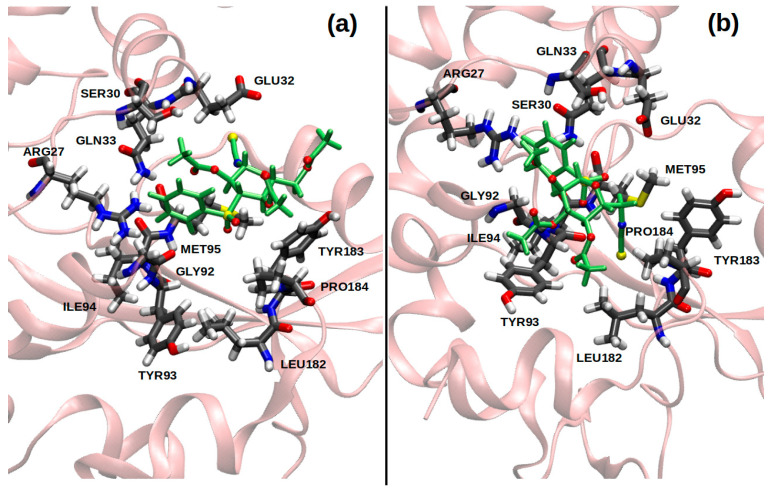
Graphical representation of the main aminoacids of STAT3 SH2 domain that contribute to the formation of the complex protein-ligand in binding pocket 1 for (**a**) compound **20** and (**b**) compound **13**. Color code: oxygen in red, carbon in gray, hydrogen in white, nitrogen in blue, sulfur in yellow, protein backbone in pink, and ligand backbone in green.

Among all evaluated compounds of both series, phenylsulfones **13** (*S*-glycoside) and **20** (*N*-glycoside) showed the most favorable predicted binding within pocket 1 of STAT3 and are represented in Figure 2.

These complexes were mainly stabilized by van der Waals and dispersion interactions, with a minor electrostatic contribution (Figure S9, Supporting Information). Detailed residue-level analyses, binding free energies, and molecular dynamics results are provided in the Supporting Information. Overall, the theoretical findings highlight pocket 1 as the preferred binding site for these ligands and provide valuable insights for understanding their structure–activity relationships. Accordingly, we investigated the antileukemic activity of both *N*- and *S*-glycoside series to compare their biological profiles, while experimental validation of STAT3 target engagement was beyond the scope of the present study.

### 3.3. Cytotoxicity Against Leukemia Cell Lines

Encouraged by the previously reported anticancer activity of ITC *N*-glycosides (**14**–**20**) in solid tumor models [29], we decided to investigate their potential antileukemic activity and extend these findings to the newly synthesized *S*-glycoside regioisomers (**6**–**13**).

To evaluate the influence of isothiocyanate positioning on antileukemic activity, we compared both series of carbohydrate-derived ITC analogs: those bearing the ITC group at the anomeric carbon (*N*-glucosides) and those with the ITC moiety relocated to position C6 (*S*-glycosides). The comparative evaluation was conducted using IC_50_ determinations in four leukemia-derived cell lines: HL-60 and U937 (acute myeloid leukemia), Jurkat (acute lymphoblastic leukemia), and OPM-2 (multiple myeloma), Table 1. For comparative purposes, the results obtained with natural ITCs derivatives previously synthesized (iberverine, racemic iberin, and cheiroline) [29] and reference antileukemic drugs, azacitidine [68] and cytarabine [69], are also included.

Among the *N*-glycosyl compounds **14**–**20** (entries 7–14, Table 1), the presence and nature of the sulfur substituent at C3 significantly influenced cytotoxic activity. The phenylsulfonyl derivative **20** exhibited potent and broad cytotoxicity across all tested cell lines (IC_50_ for HL60: 7.8 ± 0.6 µM, U937: 5.7 ± 0.3 µM, OPM-2: 10.2 ± 0.7 µM, Jurkat: 7.4 ± 0.5 µM, entry 14), clearly outperforming the alkylsulfonyl analogs, which displayed only selective activity, restricted to HL60 in the case of the methyl derivative **18** and Jurkat for the ethyl analog **19** (entries 12 and 13, respectively). In contrast, analogs bearing less oxidized sulfur moieties, such as sulfides **15** and **16** (entries 8 and 9) or the sulfoxides **(*****S*****)-17** and **(*****R*****)-17** (entries 10 and 11), were largely inactive (IC_50_ > 25 µM), underscoring the relevance of the sulfur oxidation state in determining cytotoxic potential.

Most 6-ITC *S*-glycoside derivatives exhibited cytotoxic activity across multiple cell lines (entries 1–6). Among them, the phenylsulfone **13** (entry 6) displayed the most potent and consistent profile (IC_50_ for HL60: 4.2 ± 0.4 µM, U937: 7.6 ± 0.6 µM, OPM-2: 3.4 ± 0.1 µM, Jurkat: 9.8 ± 0.6 µM), surpassing its *N*-glycosyl counterpart and highlighting the strong potential of this substitution pattern. Additionally, the sulfoxide derivatives **(*****S*****)-11** and **(*****R*****)-11** (entries 3 and 4) showed meaningful cytotoxic effects in the low micromolar range (5–16 µM). Although some differences were observed between the two sulfinyl diastereomers, their overall activity was comparable, suggesting that stereoelectronic modulation at the anomeric center may fine-tune anticancer activity without drastically altering potency.

When compared with the natural ITC derivatives iberverine, iberin, and cheiroline (entries 15–17, Table 1), compounds **13** and **20** exhibited superior or at least comparable potency across all leukemia cell lines, with the added advantage of a broader activity spectrum. Furthermore, although the reference drugs azacitidine and cytarabine (entries 18 and 19) displayed strong effects in selected models, their activity was markedly weaker or absent in others, in contrast to the consistent and balanced efficacy of **13** and **20**. These findings emphasize the potential advantage of the newly developed sulfone analogs, particularly compounds **13** and **20**, as promising leads for the development of novel antileukemic agents.

**Figure 3:**
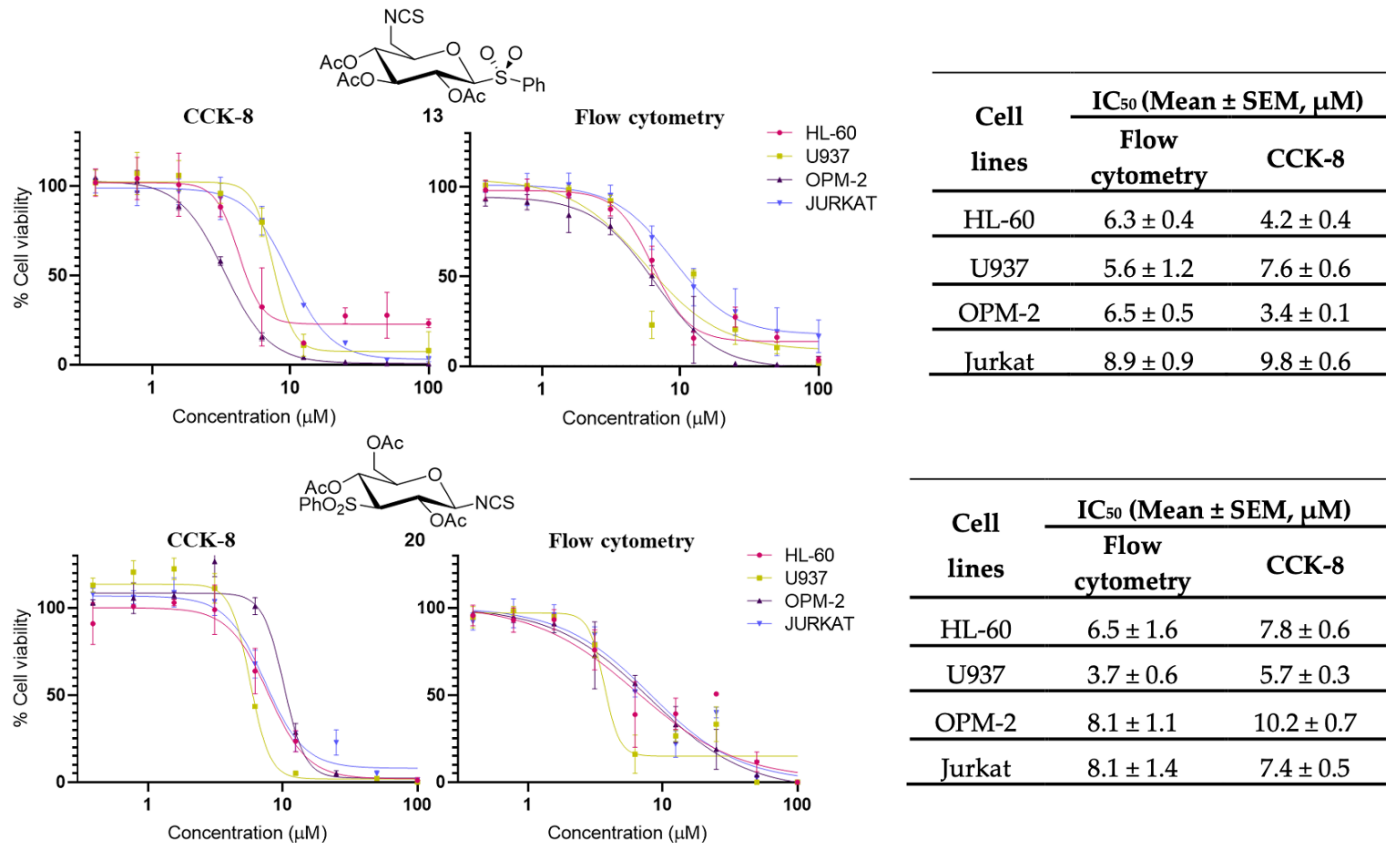
Evaluation of the effects of sulfones 13 and 20 on cell viability by CCK-8 assay and flow cytometry.

Flow cytometry based on apoptosis markers is frequently used to complement CCK-8 assays, as it provides additional insight into mechanisms of cell viability loss. In this study, flow cytometry analyses were carried out on the two most promising compounds from each family, sulfones **13** and **20** (Figure S5, Supporting Information). Notably, both methods yielded IC_50_ values of the same order of magnitude (Figure 3), supporting the robustness of the findings.

These data collectively support the conclusion that relocating the ITC group from C1 to C6 does not compromise and may enhance antileukemic efficacy, particularly when combined with phenylsulfonyl or sulfoxide substitution at C1.

### 3.4. Cytotoxicity Against Solid Tumor Cell Lines

The cytotoxic evaluation of 6-ITCs *S*-glycosyl compounds on solid tumor cell lines revealed key insights into the structure–activity relationships (SAR) governing their anticancer potential. These glucose-based ITC derivatives suggested noteworthy cytotoxicity across multiple solid tumor models, including A549 (lung adenocarcinoma), MeWo (melanoma), and T24 (bladder cancer), Table 2 (see Supplementary Materials).

A particularly significant finding was the performance of the phenylsulfone 13 (entry 10, Table 2), which consistently showed the most pronounced cytotoxic activity across all tested cancer lines, with IC_50_ values as low as 2.1 ± 0.5 µM in T24 bladder cancer. This strongly supports the earlier observation that phenylsulfonyl substituents at the anomeric position are highly favorable for bioactivity. Thioether-containing compounds such as 7 (entry 2, Y = EtS, Table 2) and 8 (entry 3, Y = PhS, Table 3) also exhibited low micromolar IC_50_ values against bladder cancer cells (IC_50_ ≈ 4.5–5.0 µM), indicating that non-oxidized sulfur substituents can retain relevant activity in this model. However, within the phenyl-substituted series, increasing sulfur oxidation state tended to be associated with improved potency across the broader panel, as reflected by the consistently lower IC50 values of the sulfoxide (***S***)-11 (entry 7, Table 2) and the sulfone 13 (entry 10, Table 2) compared with the corresponding thioether analog in A549, MeWo, and T24 cells. Additionally, sulfoxide analogs, especially the diastereomers (***R***)-10 and (***S***)-10 (entries 5 and 6, Table 2), maintained cytotoxic effects in the low micromolar range, though stereochemistry had only a modest influence on activity in this subset. In contrast, the phenyl sulfoxide enantiomers displayed a clearer stereochemical dependence, with (***S***)-11 (entry 7, Table 2) being more potent than (***R***)-11, suggesting that sulfoxide configuration can modulate activity in a cell line–dependent manner.

**Table 2:**
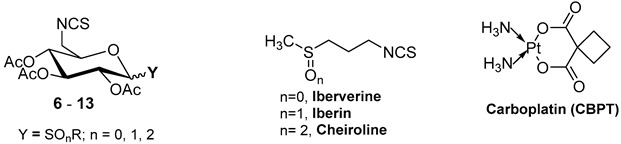
IC_50_ values (mean ± SEM, µM) of 6-ITC *S*-glycosyl derivatives 6–13, natural ITCs (iberverine, iberin, and cheiroline), and carboplatin [70] against solid tumor cell lines.

Entry	Y	Comp. ^a^	IC_50_ (Mean ± SEM, µM) (Selectivity Index) ^b^			
			HaCaT ^c^	A549 ^d^	MeWo ^e^	T24 ^f^
1	MeS	6	19.8 ± 1.4	19.0 ± 1.3 (1.0 ± 0.1)	18.8 ± 0.8 (1.1 ± 0.1)	8.1 ± 0.9 (2.5 ± 0.3)
2	EtS	7	11.1 ± 1.0	12.8 ± 0.7 (0.9 ± 0.1)	12.8 ± 1.1 (0.9 ± 0.1)	5.0 ± 0.4 (2.2 ± 0.2)
3	PhS	8	11.8 ± 1.0	24.2 ± 0.8 (0.5 ± 0.1)	8.4 ± 0.6 (1.4 ± 0.1)	4.5 ± 0.4 (2.7 ± 0.1)
4	PhS	9	6.4 ± 0.6	7.7 ± 1.0 (0.8 ± 0.0)	8.1 ± 1.3 (0.8 ± 0.1)	4.6 ± 0.6 (1.4 ± 0.2)
5	(*R*)-EtSO	(*R*)-10	12.0 ± 1.2	17.0 ± 0.1 (0.7 ± 0.1)	11.3 ± 0.7 (1.1 ± 0.1)	5.2 ± 0.4 (2.3 ± 0.2)
6	(*S*)-EtSO	(*S*)-10	9.1 ± 1.3	13.4 ± 0.8 (0.7 ± 0.1)	11.3 ± 1.1 (0.8 ± 0.1)	4.5 ± 0.9 (2.2 ± 0.5)
7	(*S*)-PhSO	(*S*)-11	3.7 ± 0.4	5.1 ± 0.2 (0.7 ± 0.1)	5.0 ± 0.6 (0.8 ± 0.1)	2.4 ± 0.5 (1.7 ± 0.3)
8	(*R*)-PhSO	(*R*)-11	9.7 ± 1.1	13.2 ± 1.0 (0.8 ± 0.1)	12.8 ± 0.4 (0.8 ± 0.1)	4.6 ± 0.3 (2.1 ± 0.3)
9	EtSO_2_	12	9.8 ± 1.1	14.6 ± 0.7 (0.7 ± 0.1)	12.4 ± 1.1 (0.8 ± 0.1)	4.6 ± 0.9 (2.3 ± 0.4)
10	PhSO_2_	13	3.2 ± 0.6	4.4 ± 0.3 (0.7 ± 0.1)	4.3 ± 0.2 (0.7 ± 0.1)	2.1 ± 0.5 (1.6 ± 0.1)
11	-	Iberverine	28.6 ± 12.0	21.9 ± 7.9 (1.2 ± 0.4)	20.5 ± 6.6 (1.2 ± 0.3)	10.1 ± 2.7 (2.4 ± 0.6)
12	-	Iberin	21.8 ± 9.8	16.6 ± 5.0 (1.2 ± 0.4)	22.1 ± 7.3 (0.9 ± 0.1)	9.2 ± 3.0 (2.1± 0.6)
13	-	Cheiroline	21.9 ± 8.1	16.9 ± 5.4 (1.2 ± 0.3)	19.6 ± 5.6 (1.0 ± 0.2)	7.9 ± 2.0 (2.3 ± 0.5)
14	-	CBPT	43.7 ± 12.7	20.3 ± 2.1 (2.1 ± 0.5)	53.2 ± 6.4 (0.8 ± 0.2)	14.5 ± 1.3 (3.3 ± 1.2)

^a^ β anomeric configuration except compound **9** with α anomeric configuration. ^b^ Data represent mean ± SEM from at least three independent experiments. The selectivity index is the mean of the selectivity indices calculated in each individual experiment. The selectivity index is calculated by dividing the IC_50_ value obtained in the HaCat cell line by that in the cancer cell lines. ^c^ Non-malignant keratinocyte. ^d^ Lung adenocarcinoma. ^e^ Melanoma. ^f^ Bladder cancer. Optimal results obtained are highlighted in grey.

Notably, although several compounds exhibited effective cytotoxicity in cancer cells, they generally lacked good selectivity against non-malignant HaCaT keratinocytes. This was particularly evident in lung and melanoma cancer cell lines, where IC_50_ values in HaCaT cells were comparable to, or even lower than, those observed in tumor cells. In contrast, a more favorable therapeutic window was observed in bladder cancer cells, where most compounds showed both stronger activity and improved selectivity over non-malignant cells, highlighting this as the most promising context for further development. These findings are in line with previous evidence underscoring the pronounced activity of SFN, particularly against bladder cancer [15].

When compared to their *N*-glycosyl ITC regioisomers, this new family of 6-ITC *S*-glycosides demonstrates superior cytotoxic activity overall, with a particularly marked improvement in bladder cancer models. Notably, the phenylsulfonyl derivative emerged as the *lead* compound in both series. However, its potency in the new series (**13**, IC_50_ = 2.1 ± 0.5 µM in T24 bladder cancer, entry 10, Table 2) was significantly higher than that of its *N*-glycosyl counterpart (**20**, IC_50_ = 16.9 ± 0.4 µM) [29], while maintaining a comparable selectivity index (SI = 1.6). From a synthetic standpoint, compound **13** also offers a practical advantage over sulfoxide analogs, as the sulfone moiety lacks sulfur-centered chirality and thus avoids the need for stereocontrol or diastereomeric separation.

In comparative terms, the activity of **13** and related sulfoxide analogs clearly surpassed that of the natural ITCs iberverine, iberin, and cheiroline (entries 11–13, Table 2), which displayed lower potency (IC_50_ ≈ 8–10 µM). It is noteworthy that these 6-ITC *S*-glycosides exhibited greater potency than carboplatin, an anticancer agent used in the clinic. These findings highlight compound **13** as a particularly promising lead, combining strong and consistent potency with practical synthetic accessibility, and underscore the broader potential of 6-ITC *S*-glycosides as a new platform for anticancer drug development.

### 3.5. Nrf2 Activation and Antioxidant Profile

The antioxidant capacity of the synthesized 6-ITC *S*-glycosyl derivatives was evaluated based on their ability to activate the Nrf2 pathway [71,72,73,74]. For this purpose, we determined the CD value, defined as the concentration of compound required to induce a twofold increase in Nrf2-regulated protein levels relative to basal conditions, in the absence of an oxidative stimulus and the values are collected in Table 3. Accordingly, Nrf2 activation in this study is supported by a functional reporter readout, whereas downstream mechanistic validation (e.g., induction of canonical Nrf2 target genes/proteins such as NQO1 and HO-1) was beyond the scope of the present work. In addition, intracellular ROS levels were not directly measured; therefore, the extent to which the observed cytotoxic effects are ROS-dependent cannot be determined and warrants further investigation. Cytotoxicity was assessed in non-malignant HaCaT keratinocytes and all compounds showed EC_50_ values above 30 µM, confirming that Nrf2 activation occurred under non-cytotoxic conditions.

**Table 3:**
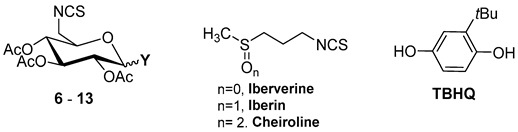
CD (µM ± st desv) values for Nrf2 activation by selected ITC derivatives **6–13** and natural ITCs (iberverine, iberin, and cheiroline).

Entry	Compound ^a^	Y	CD ± st Desv (μM)
1	6	MeS	2.64 ± 1.14
2	7	EtS	1.04 ± 0.30
3	8	PhS	0.96 ± 0.03
4	9	PhS	5.03 ± 1.66
5	(*R*)-10	(*R*)-EtSO	7.91 ± 1.88
6	(*S*)-10	(*S*)-EtSO	6.37 ± 1.61
7	(*S*)-11	(*S*)-PhSO	3.34 ± 0.86
8	(*R*)-11	(*R*)-PhSO	2.33 ± 0.25
9	12	EtSO_2_	4.33 ± 0.03
10	13	PhSO_2_	1.90 ± 0.70
11	Iberverine	-	2.94 ± 1.51
12	Iberin	-	3.12 ± 0.65
13	Cheiroline	-	3.22 ± 1.14
14	TBHQ ^b^	-	1.68 ± 0.30

^a^ β anomeric configuration, except compound **9** with α anomeric configuration. ^b^ *tert*-Butylhydroquinone (TBHQ) was used as a positive control for Nrf2 induction [75]. Optimal results obtained are highlighted in grey.

When comparing antioxidant activity across both regioisomeric series, these new 6-ITC *S*-glycosyl derivatives exhibited superior Nrf2 activation relative to their *N*-glycosyl counterparts. This difference was particularly evident in the thioether-containing compounds, which were inactive or poorly active in the *N*-glycosyl series, whereas they emerged as the most potent sulfur-based derivatives **7** and **8** among the *S*-glycosides (entries 2 and 3, Table 3). Although the phenylsulfonyl derivatives from both families displayed CD values of similar magnitude, 2.09 ± 0.13 µM for the *N*-glycoside **20** [29] and 1.90 ± 0.70 µM for the *S*-glycoside **13** (entry 10, Table 3), the overall trend indicates enhanced antioxidant potential in the sulphenyl compounds.

Anomeric configuration also influenced Nrf2 activation, particularly in the phenylthioether derivatives, where the β-anomer **8** exhibited markedly higher activity (CD = 0.96 ± 0.03 µM, entry 3, Table 3) compared to the α-anomer **9** (CD = 5.03 ± 1.66 µM, entry 4, Table 3), indicating a stereochemical preference for the β-anomeric center. In contrast, the configuration at the sulfur atom in ethyl and phenyl sulfoxide derivatives (**(*****R*****)-10** and **(*****S*****)-10**, entries 5 and 6; **(*****S*****)-11** and **(*****R*****)-11**, entries 7 and 8, Table 3) had minimal impact on antioxidant response, as both diastereomers exhibited CD values of the same order of magnitude, suggesting that sulfur-centered chirality does not significantly affect Nrf2 activation under these conditions.

When benchmarked against natural ITCs, such as iberverine, iberin, and cheiroline (entries 11–13, Table 3), which showed CD values in the range of 2.9–3.2 µM, the synthetic *S*-glycosyl derivatives, particularly **8** and **13**, demonstrated stronger Nrf2 activation, in some cases with more than a threefold improvement. Moreover, their performance also compared favorably with TBHQ (entry 14, Table 3), a well-established Nrf2 activator (CD = 1.68 ± 0.30 µM). The fact that several of the new ITCs approached or even surpassed the potency of TBHQ highlights the strong potential of this chemical series as antioxidant response modulators, while also offering structural novelty and tunable properties not present in the natural scaffolds.

### 3.6. Structure–Activity Relationship (SAR) Analysis

**Figure 4:**
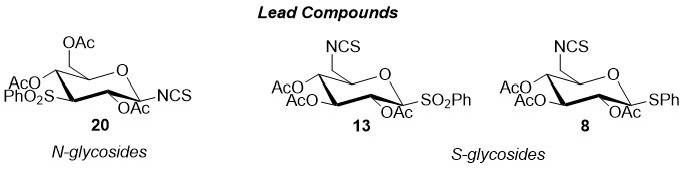
Representative structures of *lead* compounds from both regioisomeric families: *N*-glicosides [29] and *S*-glycosides.

The comparative analysis of *N*- and *S*-glycosyl ITC derivatives across leukemia and solid tumor models, along with their antioxidant properties, revealed clear structure–activity relationships that guided the selection of lead candidates (Figure 4). In the *N*-glycosyl series, cytotoxic activity was primarily influenced by the oxidation state and nature of the sulfur substituent at C3, with phenylsulfonyl derivatives showing the broadest and most potent effects. This trend was maintained and even amplified in the *S*-glycosyl analogs, where relocation of the ITC group to position C6 generally resulted in enhanced biological activity. This pattern aligns with prior in silico findings suggesting favorable SH2-domain binding affinity in such regioisomers.

Notably, as antioxidants, thioether-containing compounds, which were inactive or weakly active in the *N*-glycoside series, became the most potent Nrf2 activators in the *S*-glycoside series. Although the phenylsulfonyl derivatives from both families exhibited CD values of similar magnitude (1.90 ± 0.70 µM for **13** and 2.09 ± 0.13 µM for its *N*-glycosyl counterpart **20**), the overall antioxidant performance was superior in the *S*-glycosides. In terms of stereochemical effects, the β-anomer of the phenylthioether **8** demonstrated significantly higher Nrf2 activation than its α-anomer **9**, while sulfur-centered chirality in the sulfoxide derivatives had minimal influence, as both (*R*)- and (*S*)-diastereomers exhibited comparable CD values.

Among the *S*-glycosyl compounds, the phenylsulphone **13** emerged as the most promising multifunctional lead, combining potent cytotoxicity across leukemia and bladder cancer models (IC_50_ = 2.1 ± 0.5 µM in T24) with favorable Nrf2 activation (CD = 1.90 ± 0.70 µM) and a selectivity index (SI = 1.6) comparable to its *N*-glycosyl counterpart **20**, yet with higher potency. In addition to its biological profile, **13** offers synthetic advantages over sulfoxide analogs due to the absence of sulfur-centered chirality, simplifying its preparation and eliminating the need for stereoselective control. Another advantage is that this compound is a solid and, unlike the other regioisomer, it remains stable in water for at least 24 h without degradation (see the Supporting Information). These findings position **13** as a lead candidate for further development.

## 4. Conclusions

This study highlights the critical role of structural features, including ITC positioning, sulfur substituent identity, and glycosidic configuration, in modulating the anticancer and antioxidant activities of glycosyl isothiocyanates. Relocating the ITC group from the anomeric position to C6 in *S*-glycosyl derivatives enhanced cytotoxic potency and Nrf2 activation, especially in bladder cancer and leukemia models. Computational analyses suggest a possible interaction with STAT3, while experimental data support the activation of the Nrf2 pathway. Further studies will be required to experimentally validate STAT3 engagement. The phenylsulfone **13** emerged as a *lead* candidate, offering an optimal combination of biological activity, stability, and synthetic accessibility. These findings support the further development of 6-ITC *S*-thioglycosides as dual-action agents targeting both oncogenic and oxidative stress pathways. Future chemical optimization of this lead series will focus on maximizing potency and selectivity. For the most promising candidates, we plan to conduct mechanistic studies to confirm the role of oxidative stress in their cytotoxicity. It is well-documented that ITCs often exert cytotoxicity by modulating redox homeostasis, typically through glutathione depletion and subsequent ROS elevation [76,77,78,79]. We hypothesize that our compounds could follow a similar mechanistic pathway, which will be a primary focus of our upcoming research.

Furthermore, while the selectivity indices derived from the HaCaT model offer a valuable preliminary safety assessment for epithelial tissues, definitive validation of the systemic toxicity and therapeutic efficacy will be performed in metastatic cancer mouse models. These in vivo studies will be crucial to confirm the translational potential of these glycosyl isothiocyanates as candidate chemotherapeutic agents.

## Figures and Tables

**Table 1:**
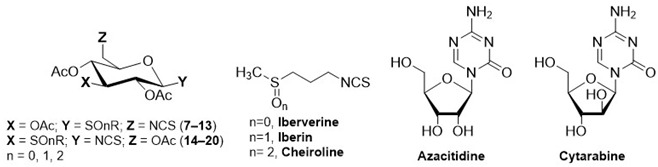
IC_50_ values (µM) of glucose-derived β-*S*-glycosyl (**7, 8 and 11**–**13**), β-*N*-glycosyl (**14**–**20**), natural isothiocyanates (iberverine, iberin, and cheiroline), and reference antileukemic drugs, azacitidine and cytarabine, against leukemia cell lines.

Entry	X	Y	Z	Comp ^a^	IC_50_ (Mean ± SEM, µM) ^b^			
					HL60 ^c^	U937 ^d^	OPM-2 ^e^	Jurkat ^f^
1	OAc	EtS	NCS	**7**	7.2 ± 0.5	22.2 ± 3.9	7.4 ± 0.3	13.4 ± 0.6
2	OAc	PhS	NCS	**8**	10.2 ± 1.2	9.9 ± 0.9	7.6 ± 0.7	20.7 ± 2.8
3	OAc	(*S*)-PhSO	NCS	**(** ***S*** **)-11**	7.6 ± 1.4	9.6 ± 0.7	8.1 ± 0.3	>25
4	OAc	(*R*)-PhSO	NCS	**(** ***R*** **)-11**	5.3 ± 0.4	11.2 ± 1.2	5.7 ± 0.4	16.2 ± 1.0
5	OAc	EtSO_2_	NCS	**12**	>25	>25	>25	>25
6	OAc	PhSO_2_	NCS	**13**	4.2 ± 0.4	7.6 ± 0.6	3.4 ± 0.1	9.8 ± 0.6
7	OAc	NCS	OAc	**14**	>25	>25	>25	>25
8	MeS	NCS	OAc	**15**	16.4 ± 2.8	15.0 ± 5.0	>25	>25
9	PhS	NCS	OAc	**16**	7.2 ± 1.0	11.4	>25	>25
10	(*S*)-PhSO	NCS	OAc	**(** ***S*** **)-** **17**	>25	>25	>25	>25
11	(*R*)-PhSO	NCS	OAc	**(** ***R*** **)-** **17**	>25	>25	> 25	>25
12	MeSO_2_	NCS	OAc	**18**	13.8 ± 1.3	> 25	> 25	>25
13	EtSO_2_	NCS	OAc	**19**	>25	>25	>25	21.9 ± 1.5
14	PhSO_2_	NCS	OAc	**20**	7.8 ± 0.6	5.7 ± 0.3	10.2 ± 0.7	7.4 ± 0.5
15	-	-	-	**Iberverine**	10.3 ± 0.5	6.7 ± 0.6	8.8 ± 0.4	9.7 ± 0.5
16	-	-	-	**Iberin**	12.7 ± 0.7	8.6 ± 1.2	14.0 ± 0.9	11.4 ± 0.7
17	-	-	-	**Cheiroline**	10.2 ± 1.5	8.3 ± 1.4	10.3 ± 0.5	9.1 ± 0.4
18	-	-	-	**Azacitidine**	>25	1.6 ± 0.4	>25	3.2 ± 1.6
19	-	-	-	**Cytarabine**	>25	0.3 ± 0.2	>25	0.1 ± 0.0

^a^ β anomeric configuration. ^b^ IC_50_ determined by CCK-8 assay. ^c^ Acute myeloid leukemia. ^d^ Acute myeloid leukemia. ^e^ Multiple myeloma. ^f^ Acute lymphoblastic leukemia.

## Data Availability

The original contributions presented in this study are included in the article/Supplementary Materials. Further inquiries can be directed to the corresponding authors.

## Supplementary Materials

The following supporting information can be downloaded at: https://www.mdpi.com/article/10.3390/antiox15010123/s1 [80], Figure S1. Time-dependent ^1^H NMR spectra (300 MHz) of the compound **13** in (CD_3_)_2_SO:D_2_O 4:1 showing its stability profile at 15 min, 1 h, 2 h, 5 h, 10 h, and 24 h.; Figure S2. Time-dependent 1H NMR spectra (300 MHz) of the compound **20** in (CD3)2SO:D2O 4:1 showing its stability profile at 15 min, 1 h, 2 h, 5 h, 10 h, and 24 h.; Figure S3. Graphical representations for the calculation of the IC_50_ values of 6-ITC *S*-glycosyl derivatives (compounds **6**–**13**), natural ITCs (iberverine, iberin and cheiroline), and carboplatin against solid tumor cell lines; Figure S4. Graphical representations for the calculation of the IC_50_ values of glucose-derived b-*N*-glycosyl (**7**, **8** and **11**–**13**), b-*S*-glycosyl (**14**–**20**), natural isothiocyanates (iberverine, iberin, and chieroline), and reference antileukemic drugs, azacitidine and cytarabine, against leukemia cell lines; Figure S5. Cell viability analysis of HL60, U937, JURKAT, and OPM-2 cell lines. Cells were analyzed after 18 h incubation with vehicle or compounds 13 and 20 (6 µM and 50 µM doses). Apoptosis and viability were assessed by Annexin V/7AAD staining using FACS analysis; Figure S6. Radar chart representation of the oral drug-likeness profile of 6-ITC glucose-based derivatives (**6–13**), natural isothiocyanates (iberverine, iberin, and chieroline) and reference drugs (azacitidine, cytarabine, and tert-Butylhydroquinone). Parameters include lipophilicity (LIPO), size (SIZE), polarity (POLAR), solubility (INSOLU), flexibility (FLEX), and saturation (INSATU); Figure S7. Schematic representation of the SH2 domain of the STAT3 protein highlighting the three predicted binding pockets within the domain structure; Table S1. Solubility parameters and Lipinski’s rule-of-five properties of 6-ITC glucose-based derivatives. The data summarize the predicted solubility profiles and key physicochemical parameters relevant to oral bioavailability, including molecular weight, hydrogen bond acceptors and donors, logP values, and compliance with Lipinski’s guidelines; Table S2. Binding free energy between the β-*S*-glycoside derivatives **7**–**13** and the most favorable binding pocket (1–3) of STAT3; Table S3. Binding free energy between each iberin derivative and the main amino acids that characterize each binding pocket; Figure S8. Color map of the binding free energies obtained for the interaction STAT3-ligand in each of the determined pockets from a previous study [8]; Figure S9. Pairwise residue decomposition of the binding free energy for every compound with the closest amino acids of the SH2 domain. The values are relative to the total binding free energy (% form): green and purple account for positive and negative contributions to the pairwise binding free energy. Total binding free energy is decomposed in four different contributions: van der Waals (vdW), electrostatic (elect), polar solvation (pol), and non-polar solvation (np).
