# Supplementary material for: Potent Nrf2-Inducing C6-Isothiocyanate Glucose Derivatives with Dual Antioxidant and Antitumor Activity
Source: Antioxidants (Basel). 2026 Jan 18;15(1):123. doi: 10.3390/antiox15010123 (PMC12837300; doi:10.3390/antiox15010123)
Supplement: Supplementary file 1 [file antioxidants-15-00123-s001.zip › antioxidants-4065744-supplementary.pdf]

Potent Nrf2-Inducing C6-Isothiocyanate Glucose Derivatives with Dual Antioxidant and Antitumor Activity.

Luis Alberto Prieto[a], Nora Khiar-Fernández[b], Rocío Calderón-Ruiz[c], Emelyne Giraud[a], José Manuel Calderón-Montaño[d], Jesús Lucia-Tamudo[e], Rafael León[f], José Antonio Pérez-Simón[c,g], Miguel López-Lázaro[d], Rocío Recio[a], Elena de la Torre[c], Victoria Valdivia[a,*], Inmaculada Fernández[a,**]

[a] *Departamento de Química Orgánica y Farmacéutica, Facultad de Farmacia, Universidad de Sevilla, 41012, Sevilla, Spain.*
[b] *Department of Organic Chemistry, School of Chemistry, Universidad Complutense de Madrid, Plaza de las Ciencias s/n, 28040, Madrid, Spain.*
[c] *Instituto de Biomedicina de Sevilla, IBiS/Hospital Universitario Virgen del Rocío/CSIC/Universidad de Sevilla, 41013, Seville, Spain*
[d] *Department of Pharmacology, Faculty of Pharmacy, University of Seville, 41012, Seville, Spain.*
[e] *Faculty of Chemistry and Pharmacy, Institute of Physical and Theoretical Chemistry, University of Regensburg, 93040, Regensburg, Germany.*
[f] *Instituto de Química Médica, Consejo Superior de Investigaciones Científicas (IQM-CSIC), C/Juan de la Cierva 3, Madrid, 28006, Spain.*
[g] *Department of Hematology, University Hospital Virgen del Rocío, Universidad de Sevilla, 41013, Seville, Spain.*

vvaldivia@us.es
inmaff@us.es

SUPPORTING INFORMATION

Table of contents

Chemical synthesis.......S2

^{1}H-NMR, ^{13}C-NMR, and HRMS of selected compounds.......S5

Stability studies.......S50

Cytotoxic activity against solid tumor cell lines of selected compounds.......S52

Cytotoxicity activity against leukemia cell lines of selected compounds.......S55

Antioxidant activity (Nrf2 induction activity).......S60

Solubility values and Lipinski rules of 6-ITC glucose-based derivatives.......S61

Binding free energies of computational studies.......S64

Bibliography.......S69

Chemical synthesis

Experimental procedures and analytical techniques

For reactions conducted under an inert atmosphere, pre-dried glassware and anhydrous solvents were consistently utilized. Solvents underwent drying with 4 Å molecular sieves, which had been previously activated by microwave irradiation and vacuum. Thin-layer chromatography (TLC) was performed using Silica Gel GF254 (Merck Life Science S.L.U., Madrid, Spain) and detected via charring with phosphomolybdic acid/EtOH or 5% H2SO4 solution in EtOH. Flash column chromatography employed Silica Gel (Merck Life Science S.L.U., Madrid, Spain; 230-400 mesh) with positive air pressure for elution. Chromatographic eluents are presented as volume/volume (v/v) ratios. Nuclear magnetic resonance (NMR) spectra were recorded on Bruker DRX-500 spectrometers (Bruker Española S.A., Madrid, Spain) at the University of Seville's nuclear magnetic resonance service. Chemical shifts are reported in parts per million (ppm) and coupling constants in hertz (Hz). Routine spectra were internally referenced to the residual proton or carbon signals of the deuterated solvent. High-resolution mass spectra were acquired on a Kratos EM-80RFA 241 MC spectrometer (Kratos Analytical Ltd., Manchester, United Kingdom), employing either electron impact (EI) or fast atom bombardment (FAB) techniques, through the University of Seville's mass spectrometry service. Optical rotations were determined using a Perkin-Elmer 341 polarimeter (PerkinElmer España S.L., Madrid, Spain).

1,2,3,4-tetra-*O*-acetyl-6-azido-6-deoxy-α, β-D-glucopyranoside, 1 [1]

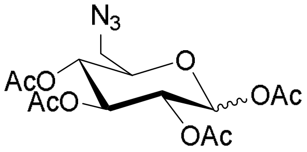

To a solution of glucose (1.00 g, 5.60 mmol) in pyridine (25 mL) at 0 °C under an argon atmosphere, a solution of *p*-toluenesulfonyl chloride (1.27 g, 6.65 mmol) in pyridine (5 mL) is added. After stirring for 18 hours, the pyridine is removed under reduced pressure. The resulting crude residue is dissolved in DMF (40 mL), and NaN_3 (1.80 g, 27.50 mmol) is added. The mixture is stirred for 3 days at 50 °C. After this time, the DMF is removed under reduced pressure. The crude is then dissolved in pyridine (50 mL) and acetic anhydride (13.00 mL, 137.50 mmol), and the mixture is stirred at room temperature for 16 hours. After this time, an ice-water mixture is added and extracted with CH_2Cl_2 (3 x 50 mL). The organic phase is washed successively with 2 M H_2SO_4 solution (3 x 50 mL), saturated $NaHCO_3$ solution (3 x 50 mL), and saturated NaCl solution, dried over anhydrous Na_2SO_4, and the solvent is evaporated under reduced pressure. The crude is purified by silica gel column chromatography (hexane/AcOEt 7:1) to obtain compound **1** (1.44 g, 3.86 mmol) as a yellow syrup. Yield: 70%. ^{1}H-NMR 500 MHz, $CDCl_3$: δ 6.35 (d, *J* = 3.67 Hz, 1Hα, H1), 5.72 (d, *J* = 8.3 Hz, 1Hβ, H1), 5 .46 (t, *J* = 9.9 Hz, 1Hα, H3), 5.24 (t, *J* = 9.4 Hz, 1Hβ, H3), 5.15 – 5.06 (m, 2Hα, H2 and H4, 1Hβ, H2 and H4), 4.08 (ddd, *J* = 10.1, 5.5 and 2.8 Hz, 1Hα, H5), 3.81 (ddd, *J* = 9.9, 5, 3 and 3.4 Hz, 1Hβ, H5), 3.41 – 3.35 (m, 1Hα, H6, 2Hβ, H6 and H6'), 3.30 (dd, *J* = 13.5 and 5, 6 Hz, 1Hα, H6'), 2.18 (s, 3Hα, -OCOC$\underline{H}_3$), 2.12 (s, 3Hβ, -OCOC$\underline{H}_3$), 2.06 (s, 3Hα, -OCOC$\underline{H}_3$), 2.05 (s, 3Hβ, -OCOC$\underline{H}_3$), 2.04 (s, 3Hβ, -OCOC$\underline{H}_3$), 2.03 (s, 3Hα, -OCOC$\underline{H}_3$), 2.02 (s, 3Hα, -OCOC$\underline{H}_3$), 2.02 (s, 3Hβ, -OCOC$\underline{H}_3$) ppm. ^{13}C-NMR 125 MHz, $CDCl_3$: δ 170.4, 170.2, 169.7, 169.5 (2), 169.3, 169.1, 168.8, 91.7, 89.0, 74.0, 72.8, 71.0, 70.3, 69.8, 69.3, 69.2, 69.1, 50.9, 50.8, 21.0,

20.9, 20 .8, 20.7 (3), 20.6 ppm. HRMS: Calculated for $C_{14}H_{19}O_9N_3Na$ $[M+Na]^+$: 396.1014; found 396.1007 (-1.7 ppm).

General procedure for the synthesis of ethyl and phenylthioglycosides

To a solution of **1** (100 mol%) and the corresponding thiol (200 mol%) in CH_2Cl_2, under an argon atmosphere and at room temperature, boron trifluoride etherate (400 mol%) is added dropwise. The reaction is stirred until the starting product is consumed (20 h). After this time, the mixture is treated with saturated $NaHCO_3$ solution. The aqueous phase is extracted with CH_2Cl_2 (3 x 25 mL) and the combined organic phases are washed with saturated NaCl solution (1 x 25 mL), dried over anhydrous Na_2SO_4, and the solvent is evaporated under reduced pressure. The crude is purified by silica gel column chromatography.

Ethyl 2,3,4-tri-*O*-acetyl-6-azido-1,6-dideoxy-1-thio-β-D-glucopyranoside, 3 [2]

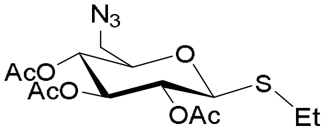

It is synthesized following the general procedure starting from compound **1** (275 mg, 0.74 mmol), ethanethiol (0.11 mL, 1.48 mmol) in CH_2Cl_2 (5 mL), and boron trifluoride etherate (0.35 mL, 2.96 mmol). The crude is purified by silica gel column chromatography (hexane/AcOEt 5:1) to obtain compound **3** (79 mg, 0.21 mmol) as a yellow syrup.

Yield: 29%. ^{1}H-NMR 500 MHz, $CDCl_3$: δ 5.23 (t, *J* = 9.4 Hz, 1H, H3), 5.05 (t, *J* = 9.7 Hz, 1H, H2), 5.02 (t, *J* = 9.7 Hz, 1H, H4), 4.54 (d, *J* = 10.1 Hz, 1H, H1), 3.69 (ddd, *J* = 9.9, 6.7 and 2.8 Hz, 1H, H5), 3.37 (dd, *J* = 13.5 and 6.6 Hz, 1H, H6), 3.30 (dd, *J* = 13.5 and 2.8 Hz, 1H, H6'), 2.80 – 2.68 (m, 2H, -SC$\underline{H}_2$CH$_3$), 2.07 (s, 3H, C$\underline{H}_3$COO-), 2.04 (s, 3H, C$\underline{H}_3$COO-), 2.02 (s , 3H, C$\underline{H}_3$COO-), 1.29 (t, *J* = 7.5 Hz, 3H, -SCH$_2$C$\underline{H}_3$) ppm. ^{13}C-NMR 125 MHz, $CDCl_3$: δ 170.3, 169.6, 169.5, 83.3, 77.5, 73.9, 70.0, 69.6, 51.3, 23, 9, 20.8, 20.7 (2), 14.8 ppm. HRMS: Calculated for $C_{14}H_{21}O_7N_3NaS$ $[M+Na]^+$: 398.0992; found 398.0983 (-2.3 ppm).

Phenyl 2,3,4-tri-*O*-acetyl-6-azido-1,6-dideoxy-1-thio-α and β-D-glucopyranoside, 4 and 5 [3]

It is carried out following the general procedure starting from compound **1** (500 mg, 1.34 mmol), thiophenol (0.27 mL, 2.68 mmol) in CH_2Cl_2 (4 mL), and boron trifluoride etherate (0.63 mL, 5.36 mmol). In this way, an orange solid is obtained as a mixture of the two α:β anomers in a 0.2:1 ratio. After purification by silica gel column chromatography (hexane/AcOEt 7:1), the α anomer (40 mg, 0.94 mmol) is obtained as a brown syrup and the β anomer (340 g, 0. 80 mmol) as a yellow syrup, with an overall yield of 60% (7% α and 53% β).

Phenyl 2,3,4-tri-*O*-acetyl-6-azido-1,6-dideoxy-1-thio-β-D-glucopyranoside, 4

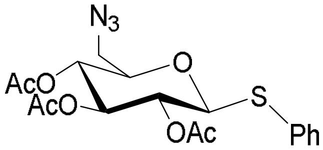

Yield: 53%. ^{1}H-NMR 500 MHz, $CDCl_3$: δ 7.51 – 7.50 (m, 2H, -SC$_6\underline{H}_5$), 7.33 – 7.32 (m, 3H, -SC$_6\underline{H}_5$), 5.20 (t, *J* = 9.4 Hz, 1H, H3), 4.95 (dd, *J* = 9.4 and 3.2 Hz, 1H, H2), 4.93 (dd, *J* = 9.5 and 3.4 Hz, 1H, H4), 4.70 (d, *J* = 10.1 Hz, 1H, H1), 3.64 (ddd, *J* = 9.9, 6.5 and 3.0 Hz, 1H, H5), 3.34 (dd, *J* = 13.4 and 6.5 Hz, 1H, H6), 3.29 (dd, *J* = 13.4 and 2.9 Hz, 1H, H6'), 2.08 (s , 3H, C$\underline{H}_3$COO-), 2.00 (s, 3H, C$\underline{H}_3$COO-), 1.97 (s, 3H, C$\underline{H}_3$COO-) ppm. ^{13}C-NMR 125 MHz, $CDCl_3$: δ 170.3, 169.6, 169.4, 133.9 (2),

131.1, 129.2 (2), 128.9, 85.9, 77.2, 74.0, 70.1, 69.4, 51.5, 20.9, 20.7 (2) ppm. HRMS: Calculated for $C_{18}H_{21}O_7N_3NaS$ $[M+Na]^+$: 446.0992; found 446.0981 (-2.5 ppm).

Phenyl 2,3,4-tri-*O*-acetyl-6-azido-1,6-dideoxy-1-thio-α-D-glucopyranoside, 5

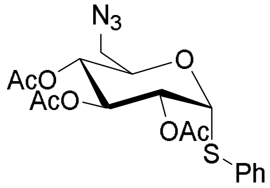

Yield: 7%. ^{1}H-NMR 500 MHz, $CDCl_3$: δ 7.47 – 7.45 (m, 2H, -$SC_6\underline{H}_5$), 7.34 – 7.28 (m, 3H, -$SC_6\underline{H}_5$), 5.92 (d, *J* = 5.7 Hz, 1H, H1), 5.42 (t, *J* = 9.8 Hz, 1H, H3), 5.08 (dd, *J* = 10.4 and 5.8 Hz, 1H, H2), 5.01 (t, *J* = 9.7 Hz, 1H, H4), 4.50 (ddd, *J* = 10.0, 6.5 and 2.8 Hz, 1H, H5), 3.34 (dd, *J* = 13.4 and 6.4 Hz, 1H, H6), 3.27 (dd, *J* = 13.4 and 2.7 Hz, 1H, H6'), 2.10 (s, 3H, $C\underline{H}_3COO$ -), 2.06 (s, 3H, $C\underline{H}_3COO$-), 2.04 (s, 3H, $C\underline{H}_3COO$-) ppm. ^{13}C-NMR 125 MHz, $CDCl_3$: δ 170.1, 170.0, 169.8, 132.5, 132.0, 129.4, 128.0, 85.2, 70.4, 69.9, 69.7, 51.2, 20.8 (2) ppm. HRMS: Calculated for $C_{18}H_{21}O_7N_3NaS$ $[M+Na]^+$: 446.0992; found 446.0995 (0.5 ppm).

^{1}H-NMR, ^{13}C-NMR, and HRMS of selected compounds

1,2,3,4-tetra-*O*-acetyl-6-azido-6-deoxy-α,β-D-glucopyranoside, (1)

^{1}H-NMR (500 MHz, $CDCl_3$)

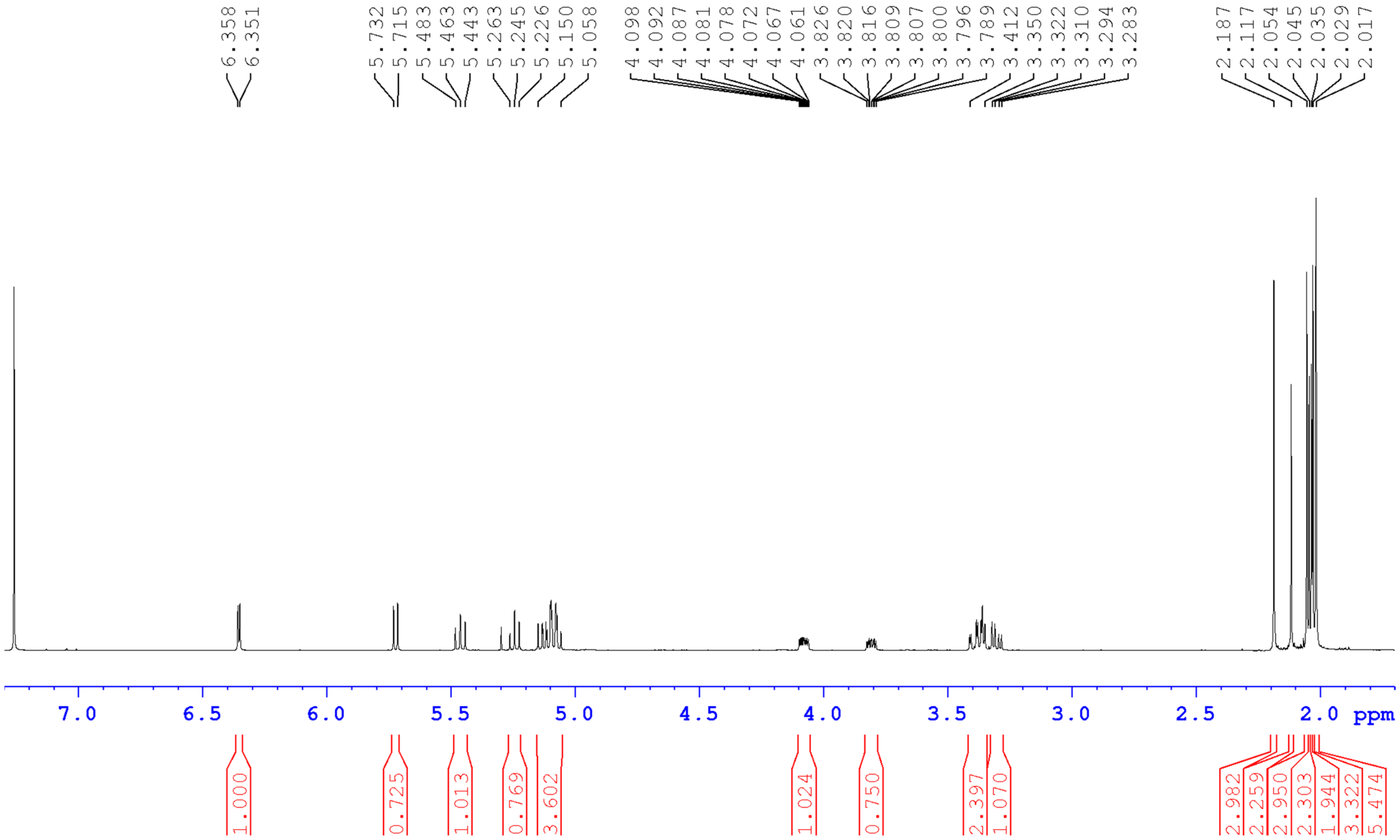

^{13}C-NMR (125 MHz, $CDCl_3$)

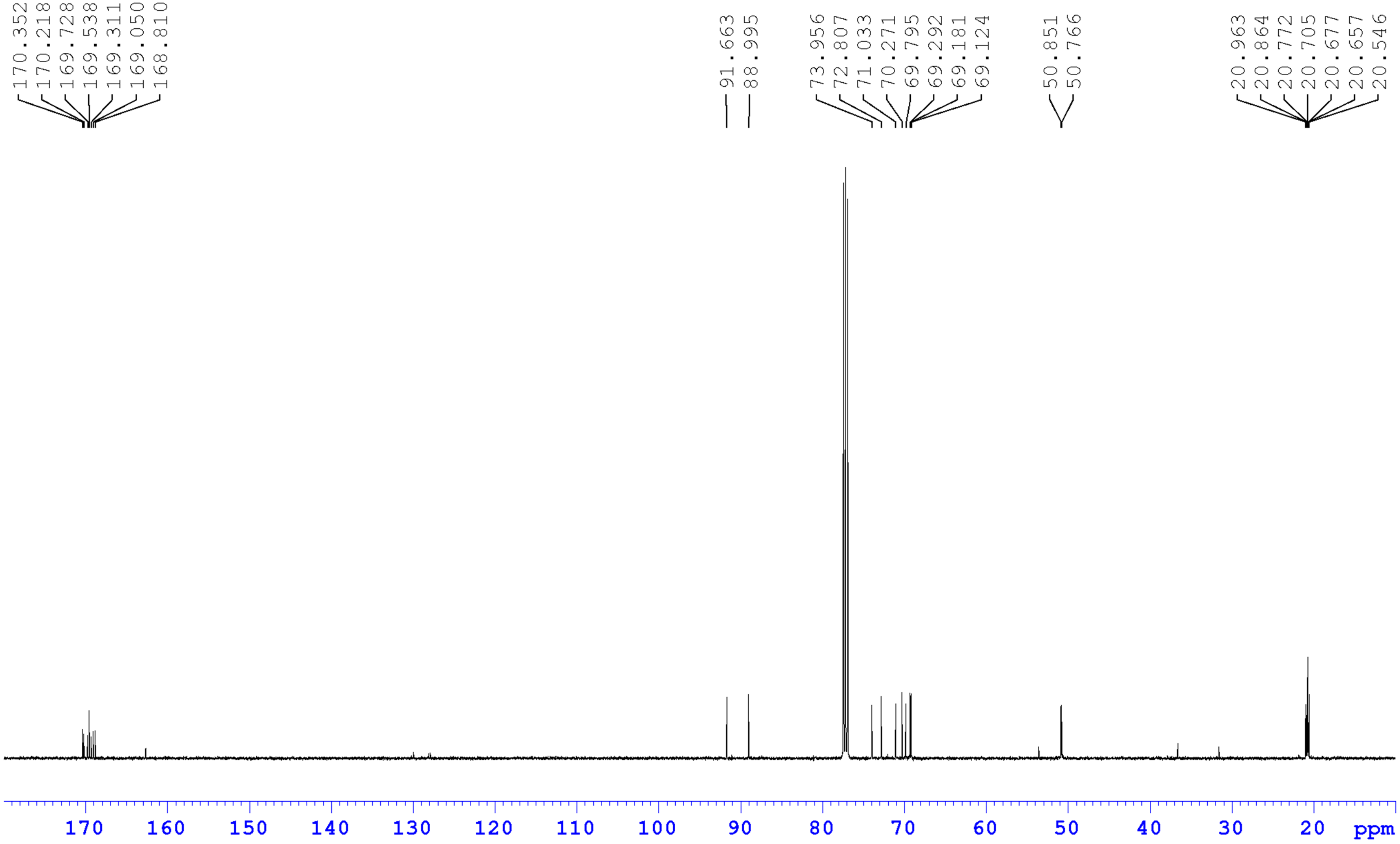

HRMS

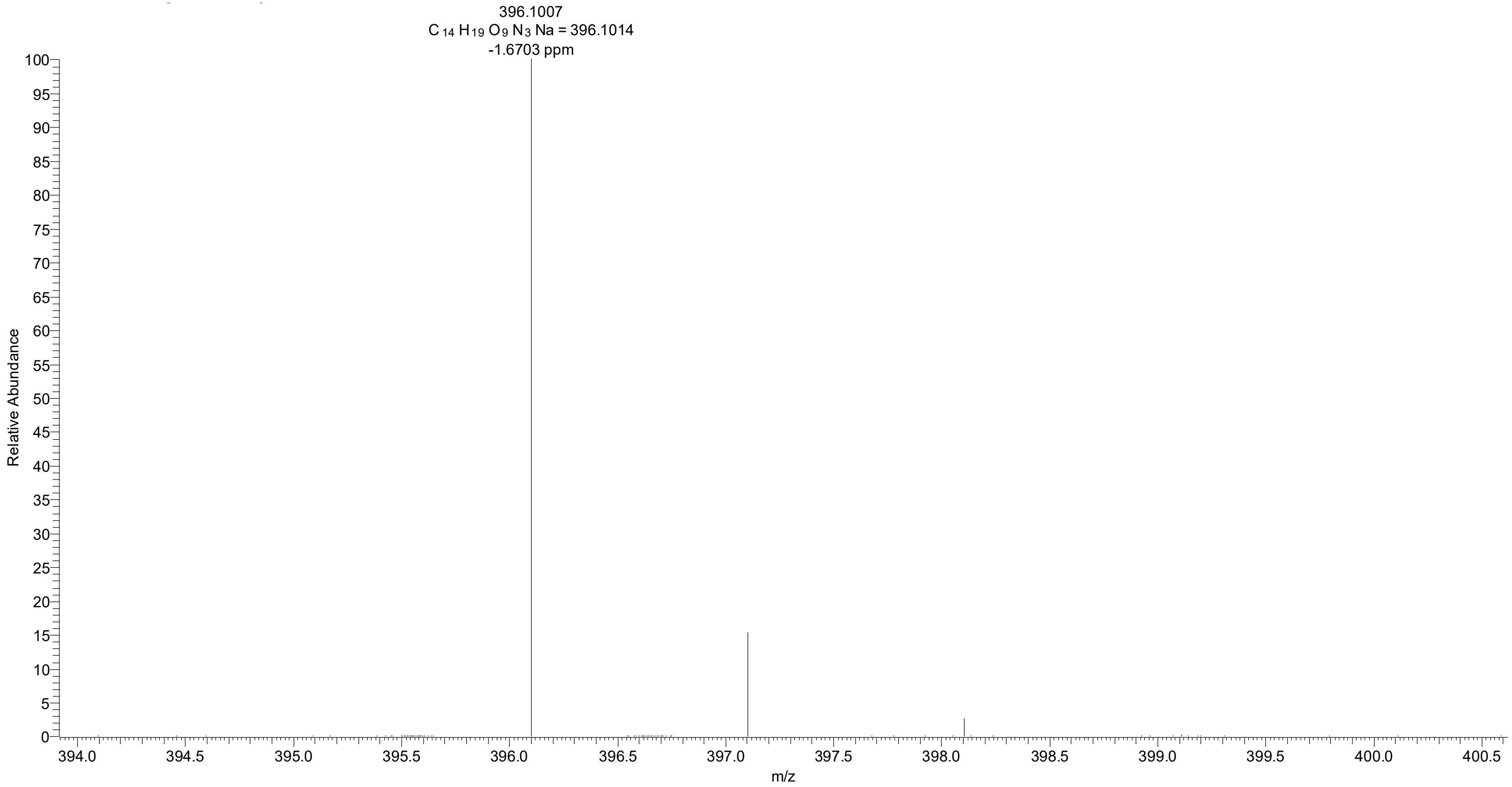
396.1007
C 14 H 19 O 9 N 3 Na = 396.1014
-1.6703 ppm
Relative Abundance
m/z

Methyl 2,3,4-tri-*O*-acetyl-6-azido-1,6-dideoxy-1-thio-β-D-glucopyranoside (2)

^{1}H-NMR (500 MHz, $CDCl_3$)

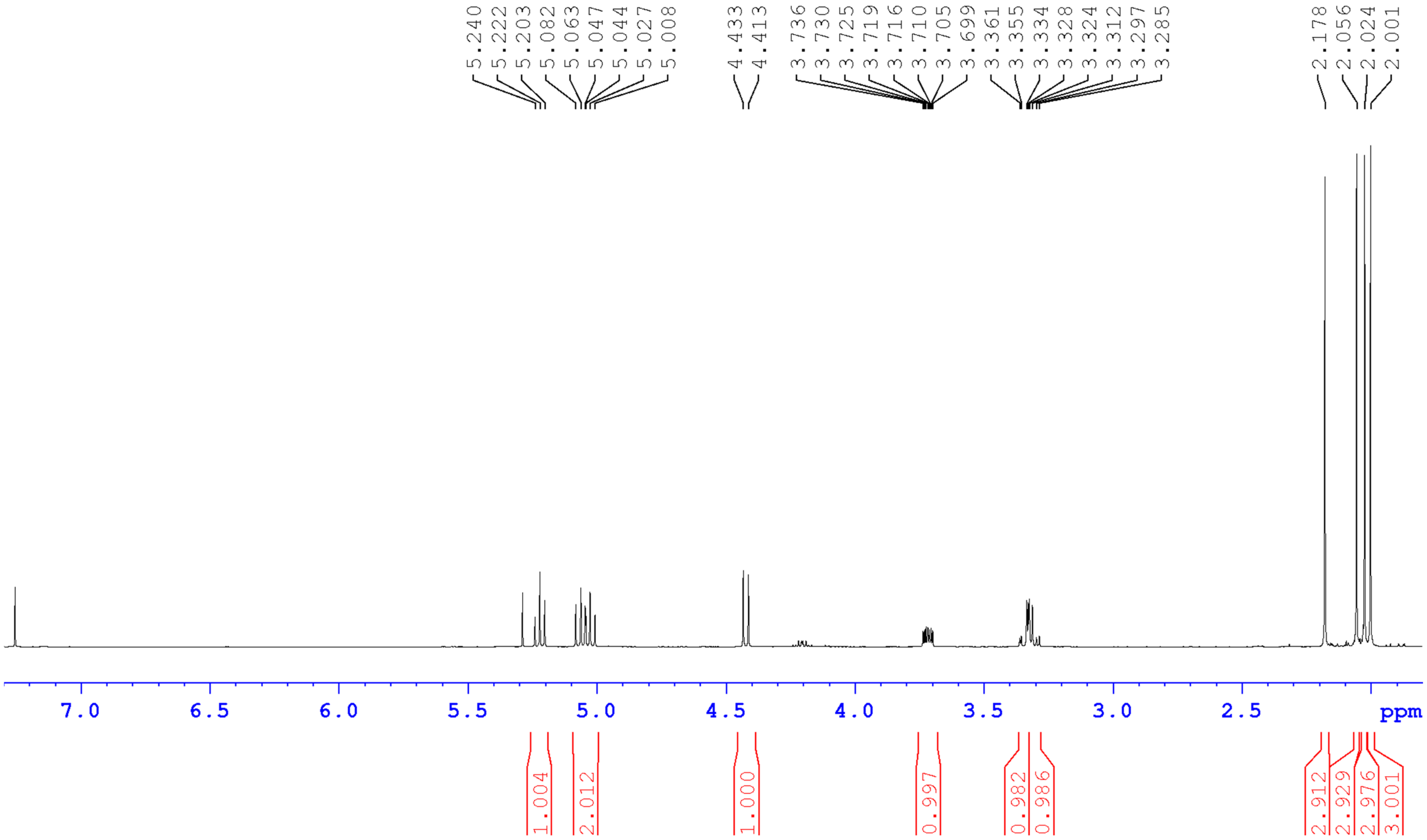

^{13}C-NMR (125 MHz, $CDCl_3$)

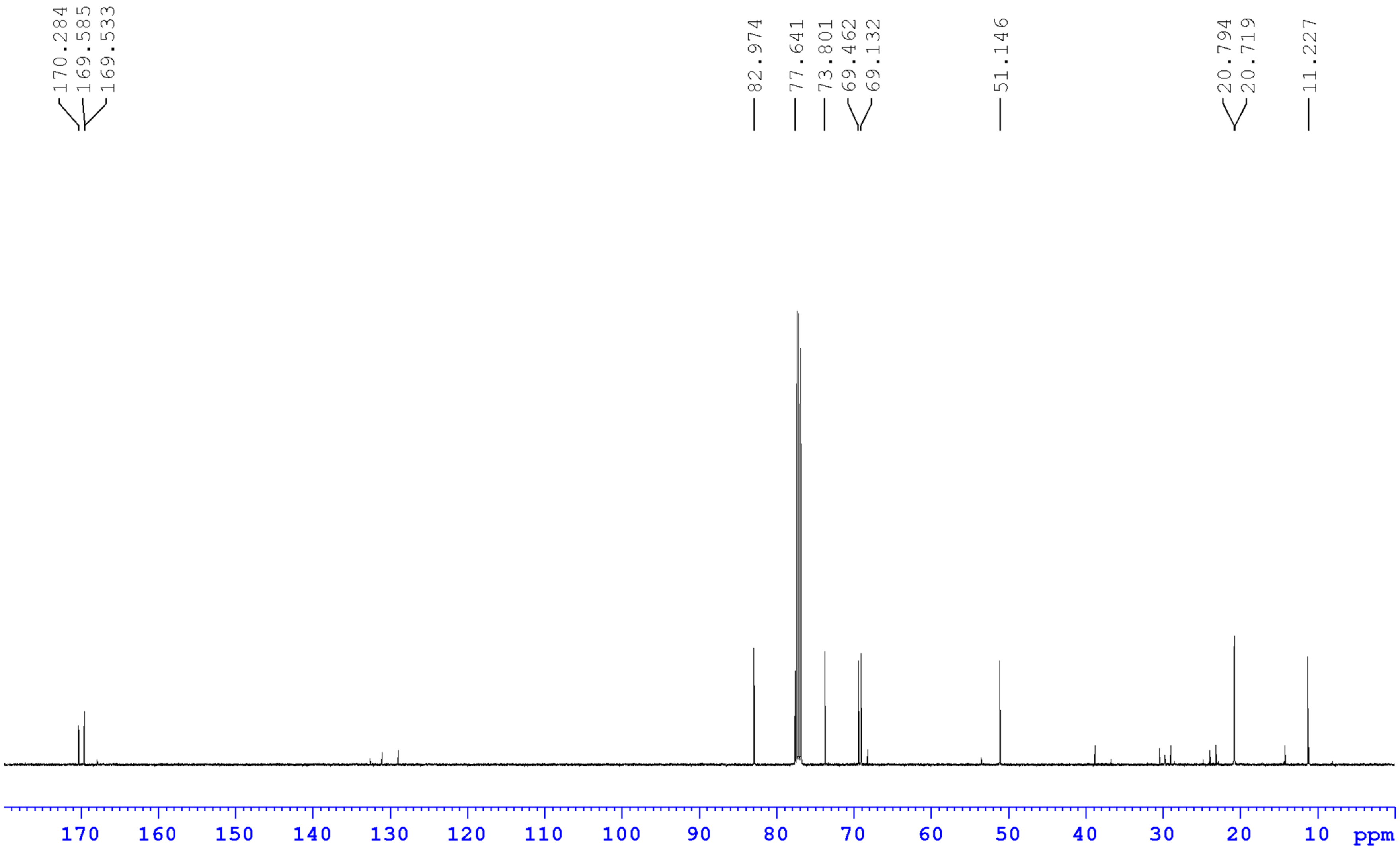

HRMS

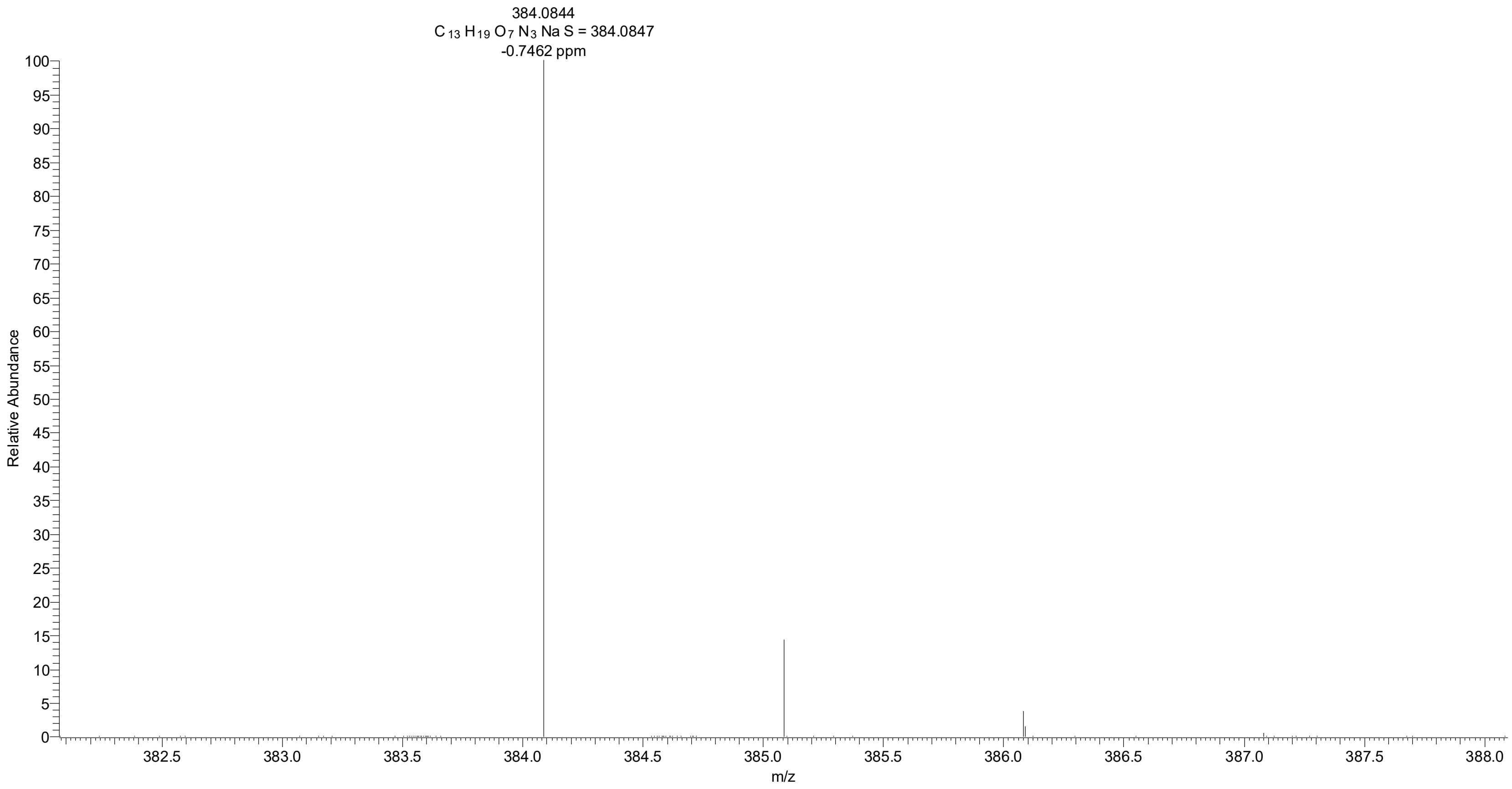
384.0844
C 13 H 19 O 7 N 3 Na S = 384.0847
-0.7462 ppm
Relative Abundance
m/z

Ethyl 2,3,4-tri-*O*-acetyl-6-azido-1,6-dideoxy-1-thio-β-D-glucopyranoside (3)

^{1}H-NMR (500 MHz, $CDCl_3$)

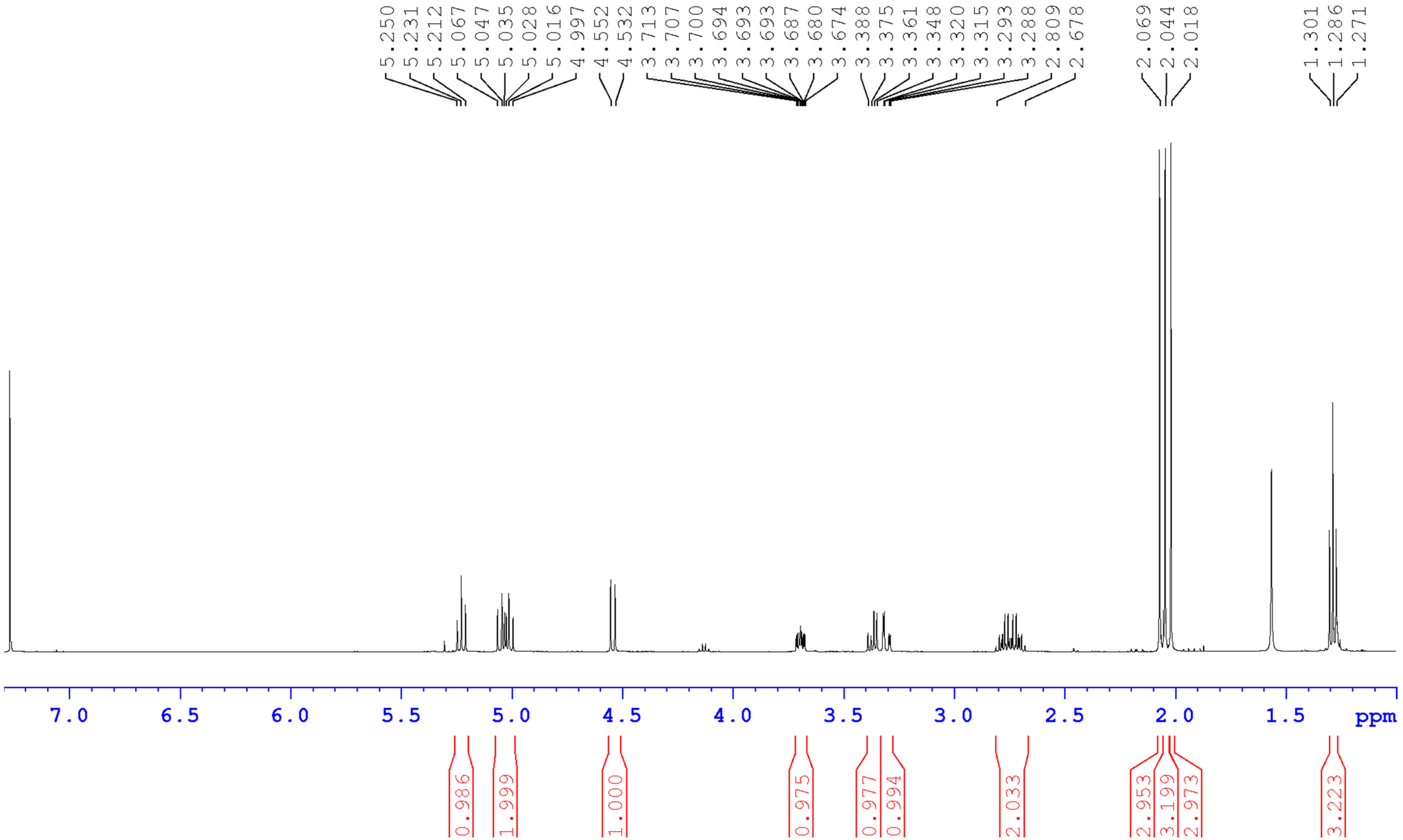

^{13}C-NMR (125 MHz, $CDCl_3$)

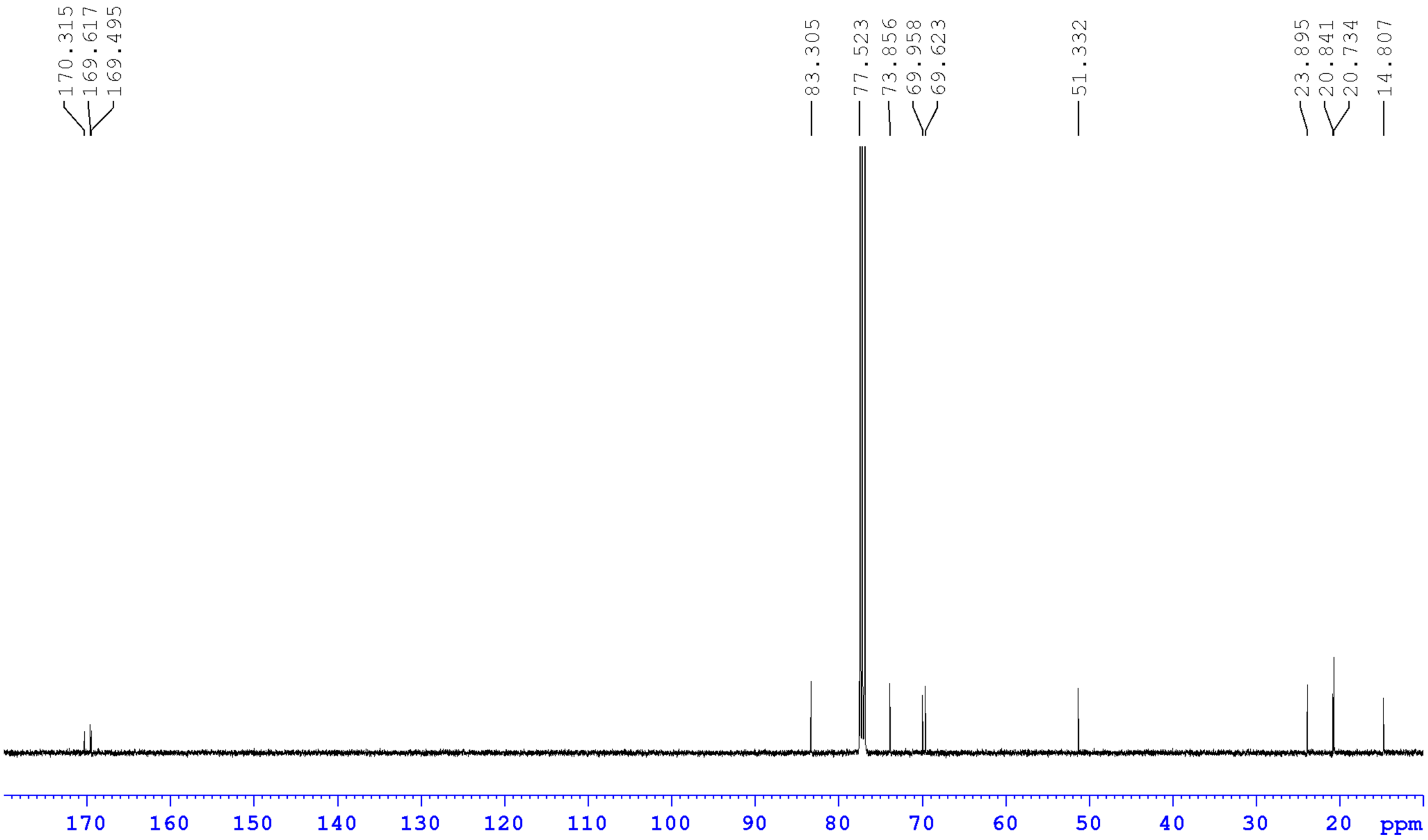

HRMS

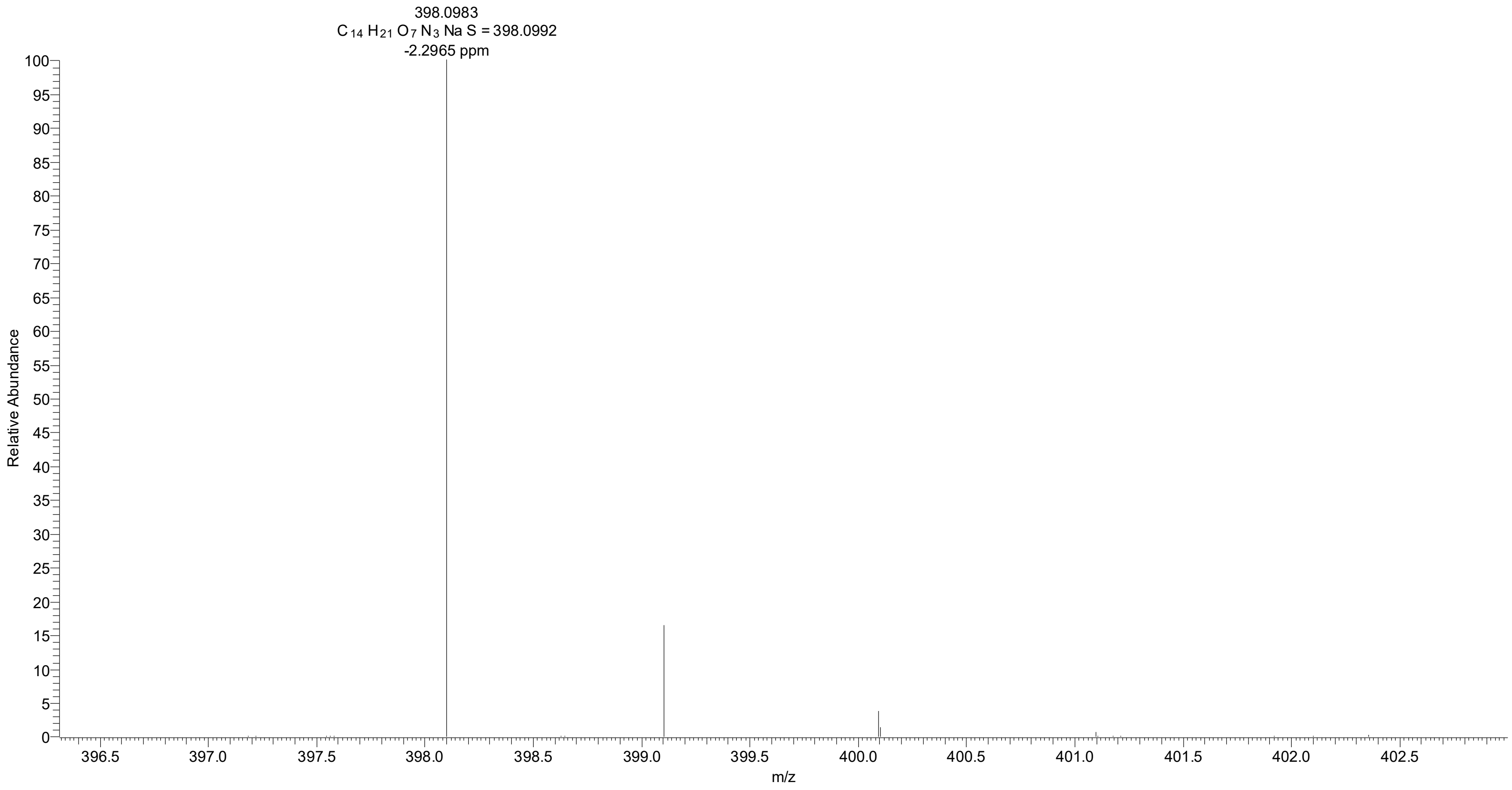

398.0983
C 14 H 21 O 7 N 3 Na S = 398.0992
-2.2965 ppm
Relative Abundance
m/z

Phenyl 2,3,4-tri-*O*-acetyl-6-azido-1,6-dideoxy-1-thio-β-D-glucopyranoside (4)

^{1}H-NMR (500 MHz, $CDCl_3$)

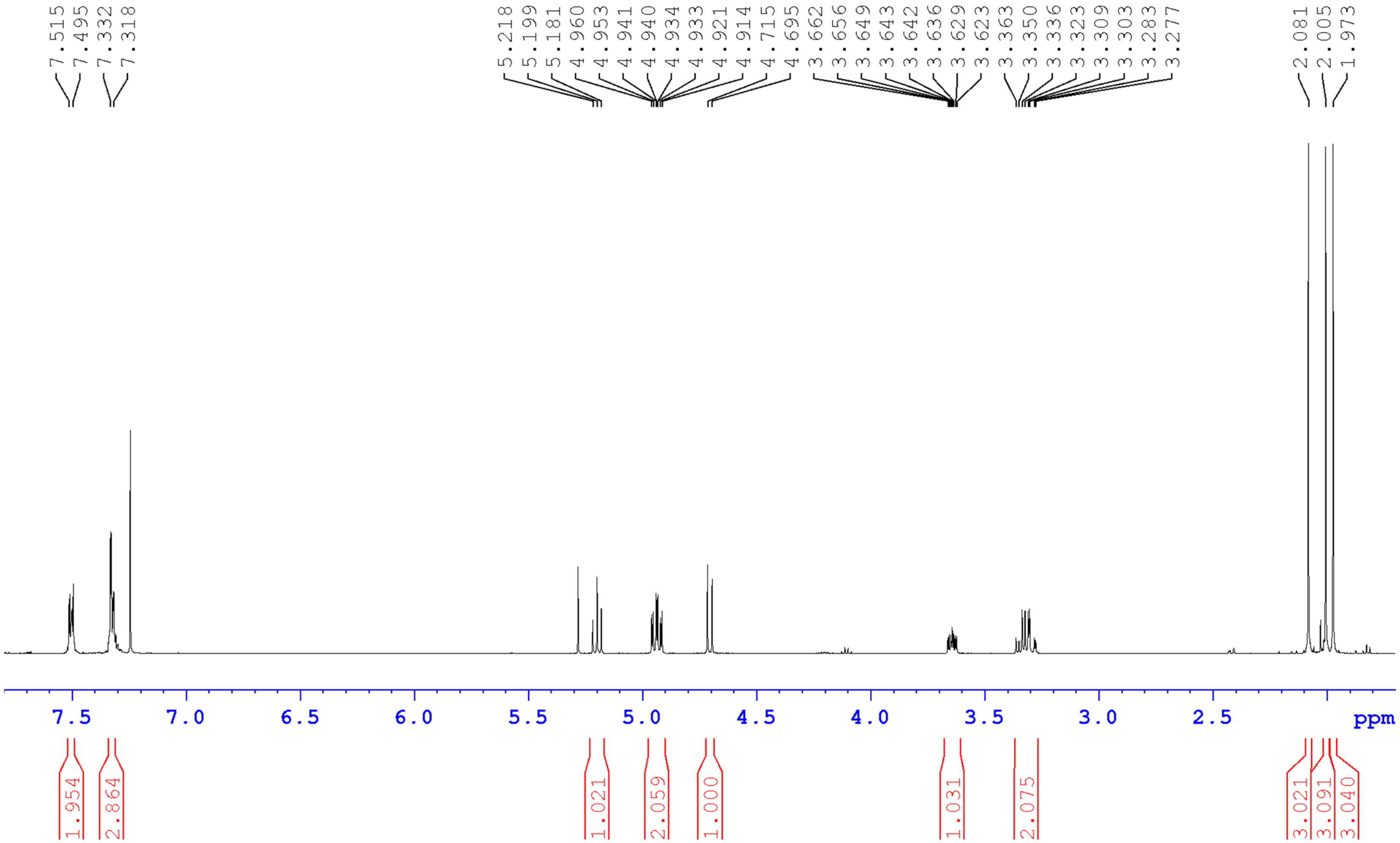

^{13}C-NMR (125 MHz, $CDCl_3$)

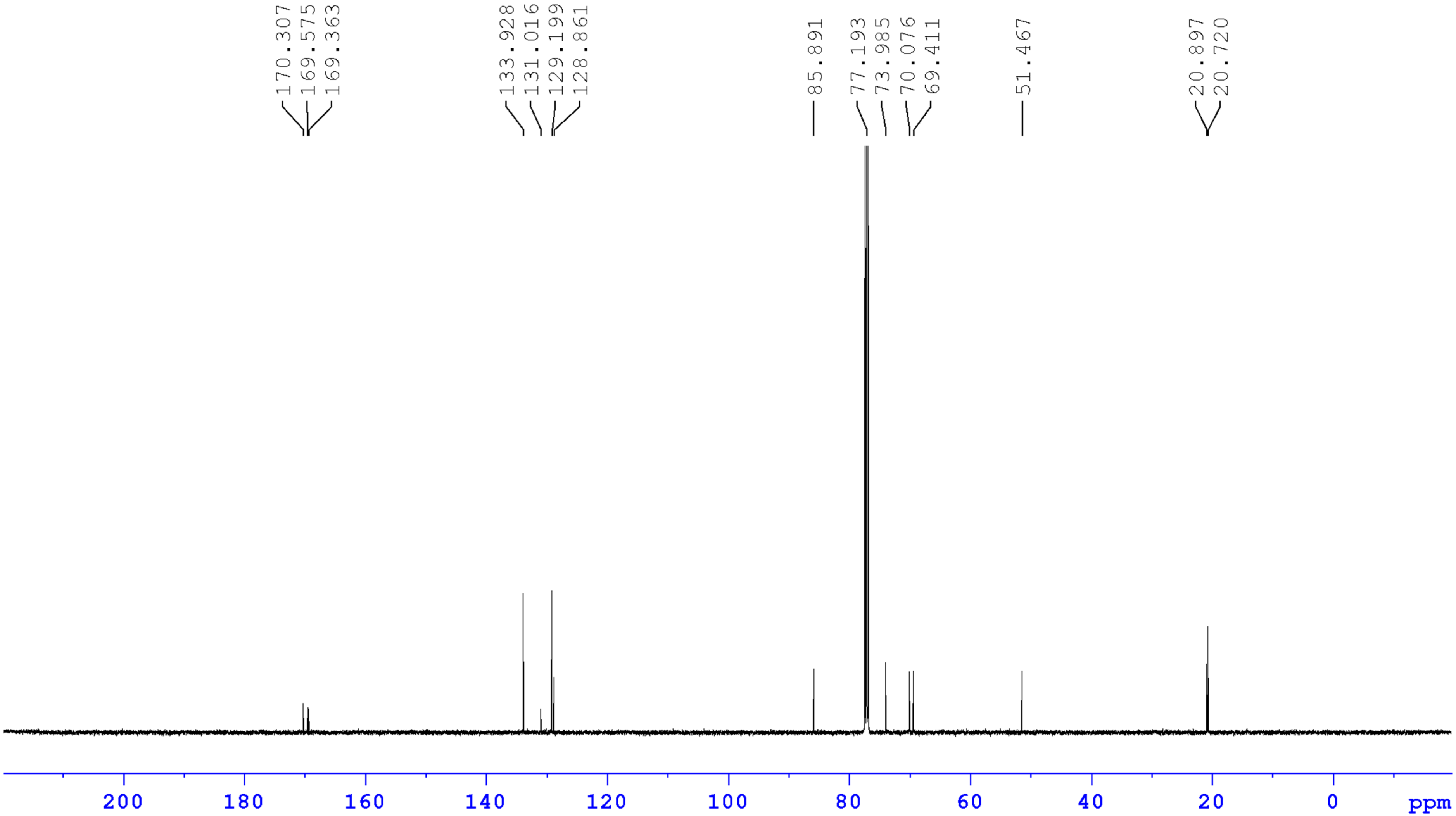

HRMS

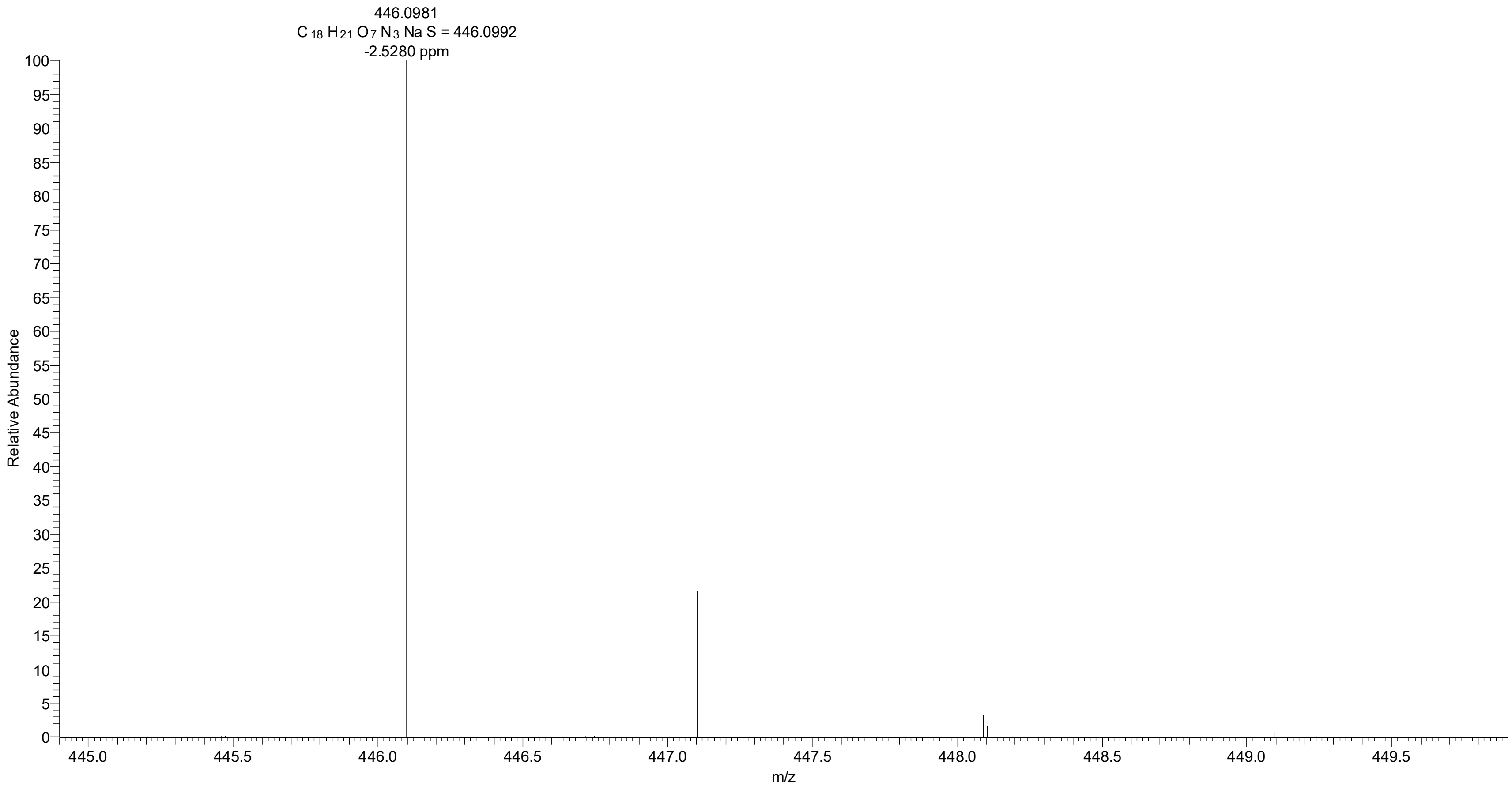
446.0981
C 18 H 21 O 7 N 3 Na S = 446.0992
-2.5280 ppm
Relative Abundance
m/z
0
5
10
15
20
25
30
35
40
45
50
55
60
65
70
75
80
85
90
95
100
445.0
445.5
446.0
446.5
447.0
447.5
448.0
448.5
449.0
449.5

Phenyl 2,3,4-tri-*O*-acetyl-6-azido-1,6-dideoxy-1-thio-α-D-glucopyranoside (5)

^{1}H-NMR (500 MHz, $CDCl_3$)

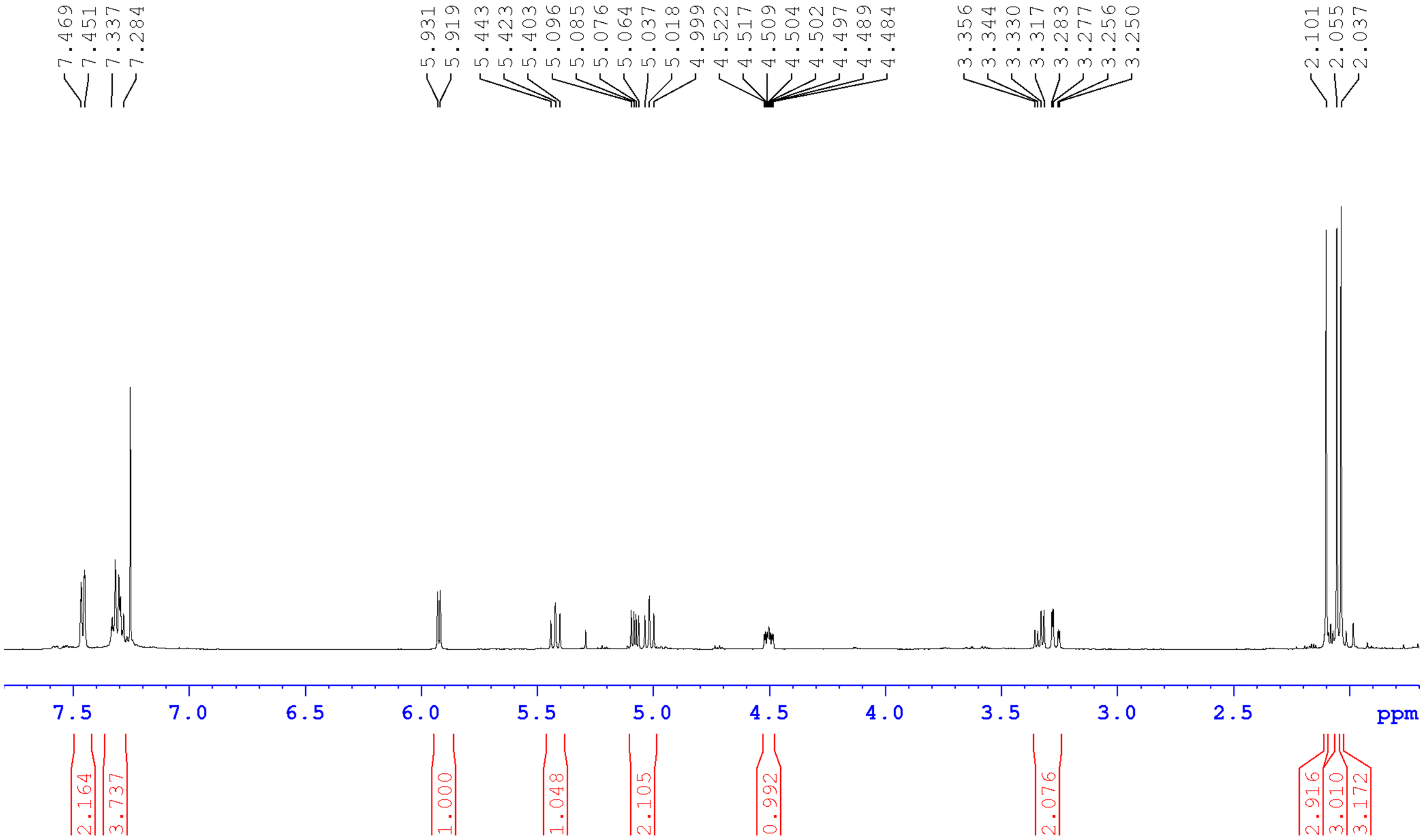

^{13}C-NMR (125 MHz, $CDCl_3$)

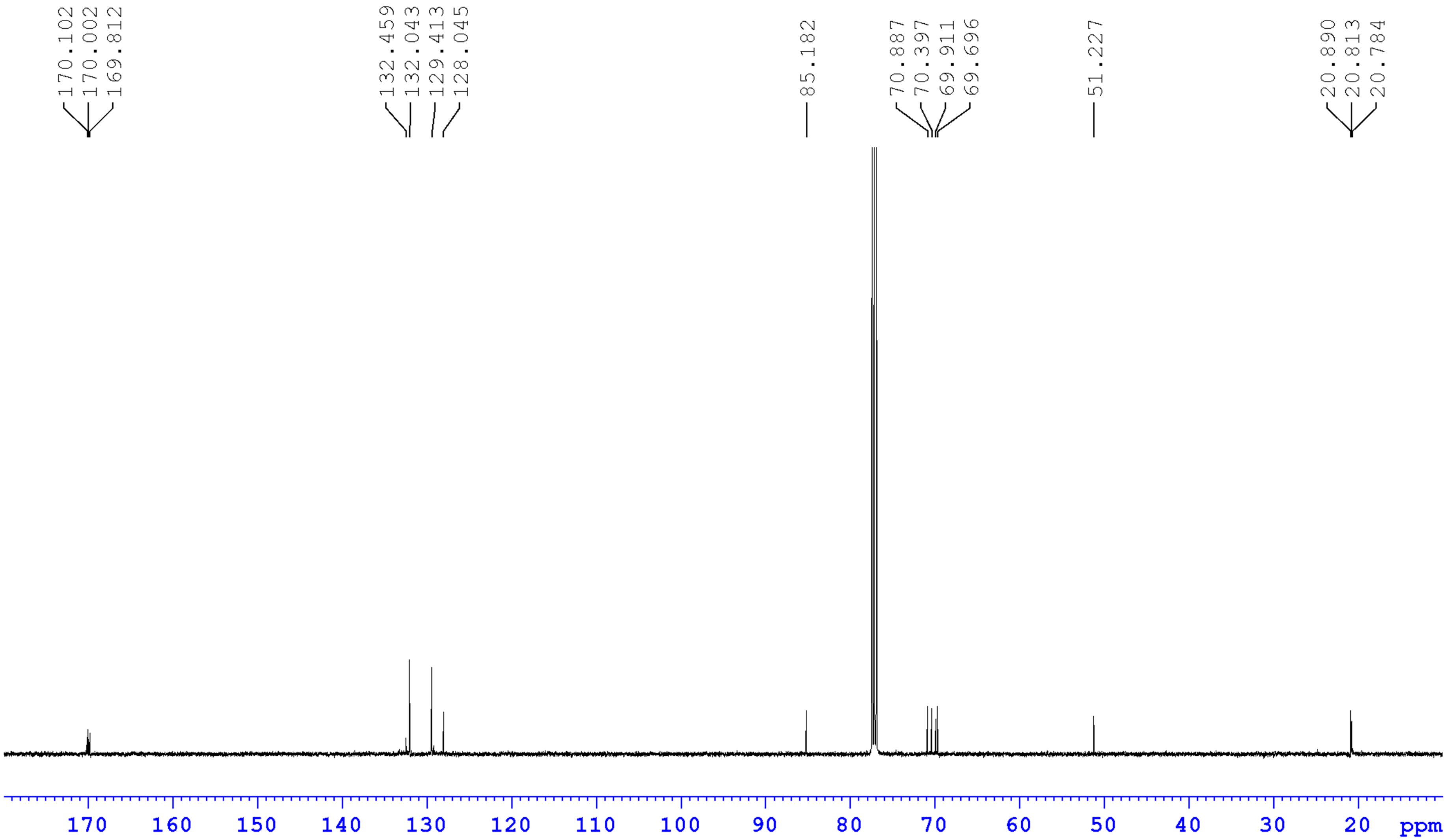

HRMS

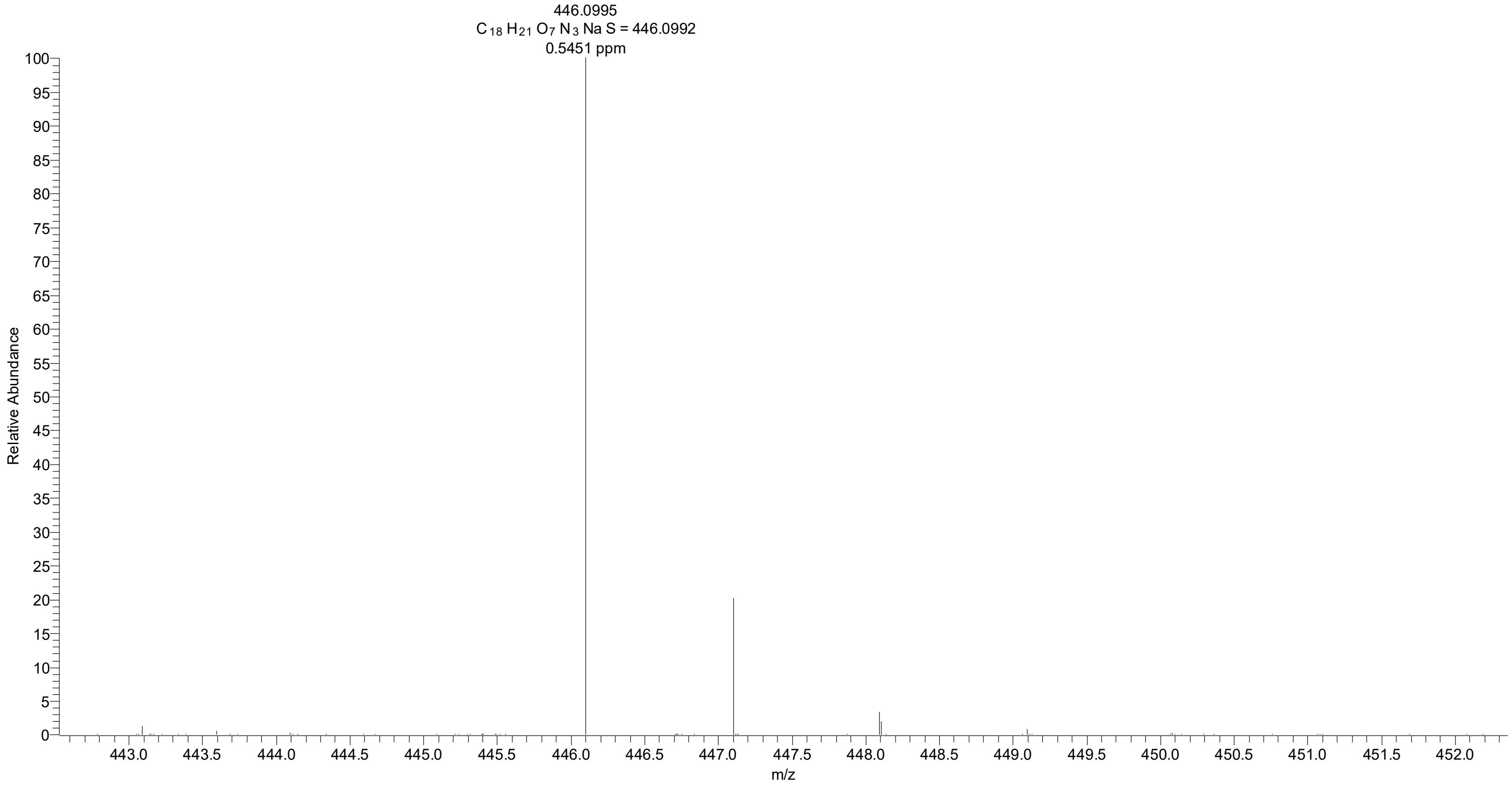
446.0995
C 18 H 21 O 7 N 3 Na S = 446.0992
0.5451 ppm
Relative Abundance
m/z

Methyl 2,3,4-tri-*O*-acetyl-6-isothiocyanato-1,6-dideoxy-1-thio-β-D-glucopyranoside (6)

^{1}H-NMR (500 MHz, $CDCl_3$)

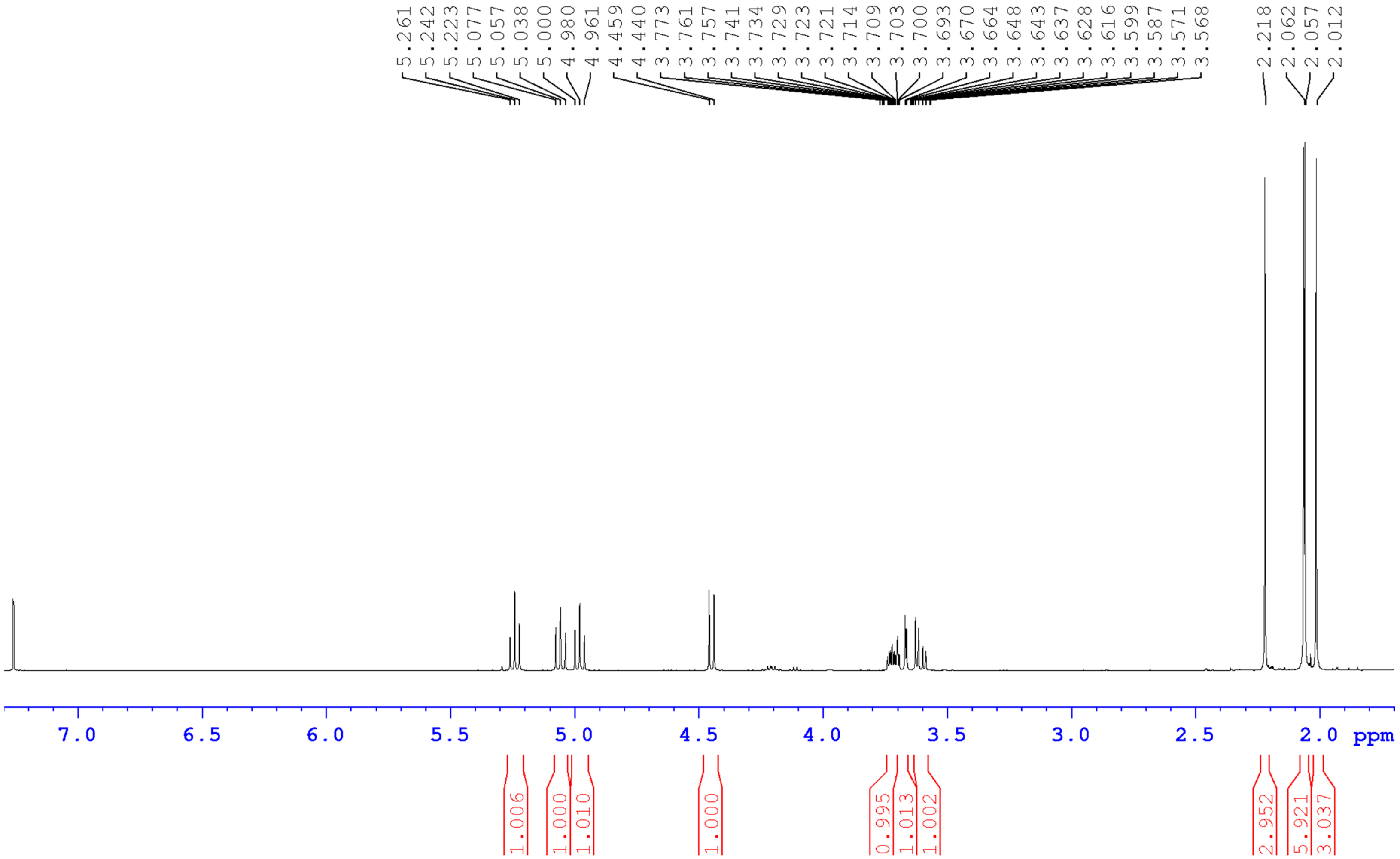

^{13}C-NMR (125 MHz, $CDCl_3$)

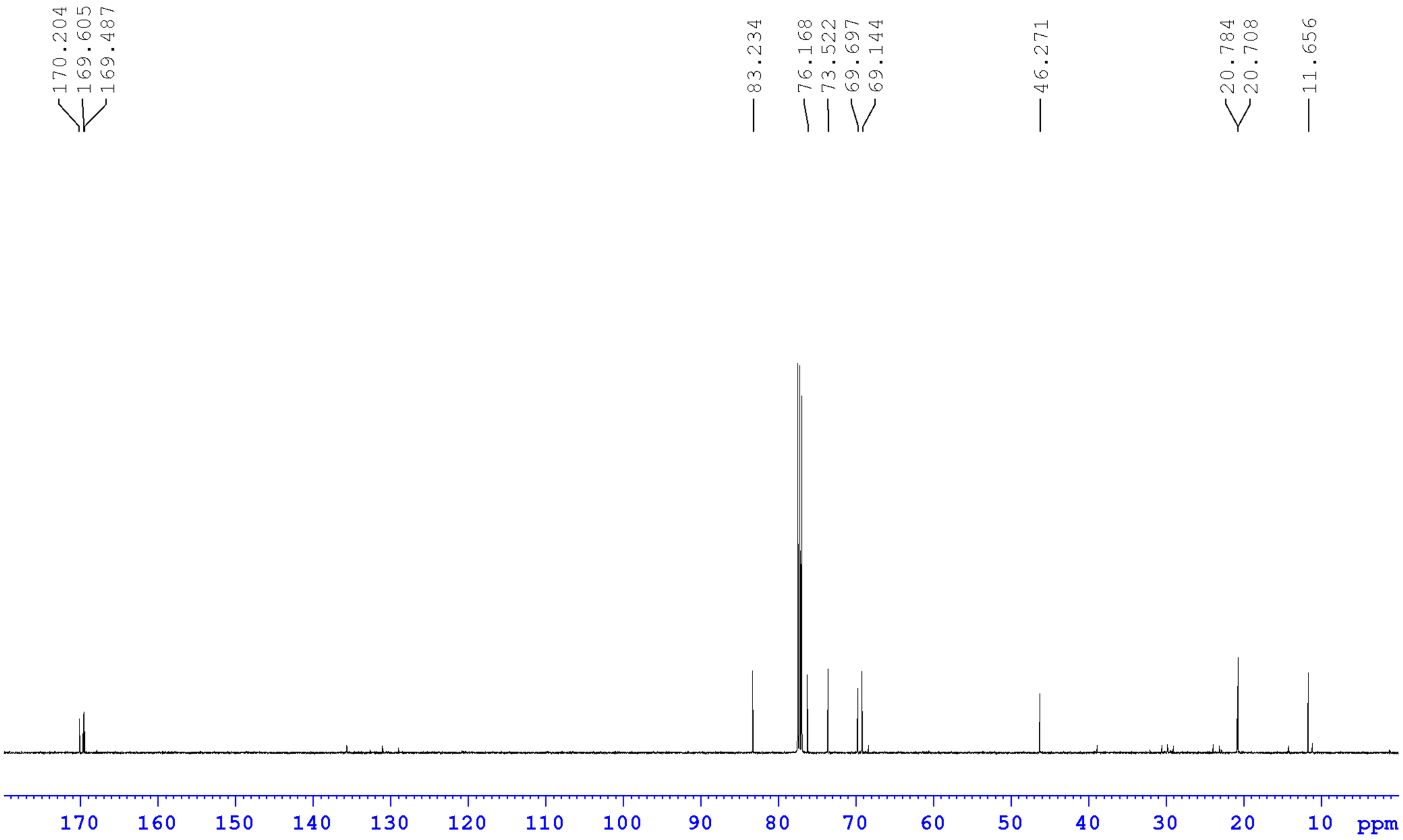

HRMS

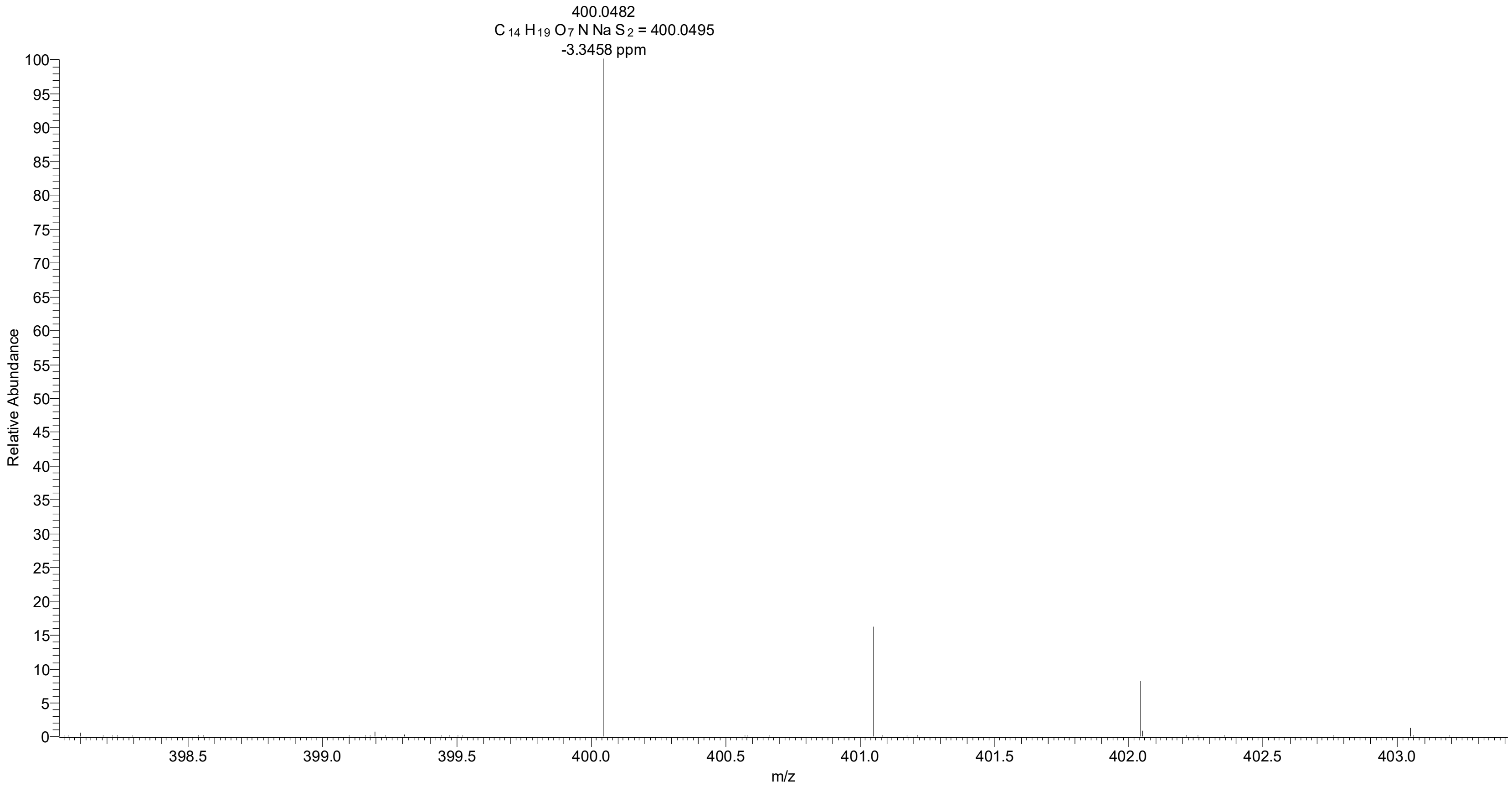
400.0482
C 14 H 19 O 7 N Na S 2 = 400.0495
-3.3458 ppm
Relative Abundance
m/z

Ethyl 2,3,4-tri-*O*-acetyl-6-isothiocyanato-1,6-dideoxy-1-thio-β-D-glucopyranoside (7)

^{1}H-NMR (500 MHz, $CDCl_3$)

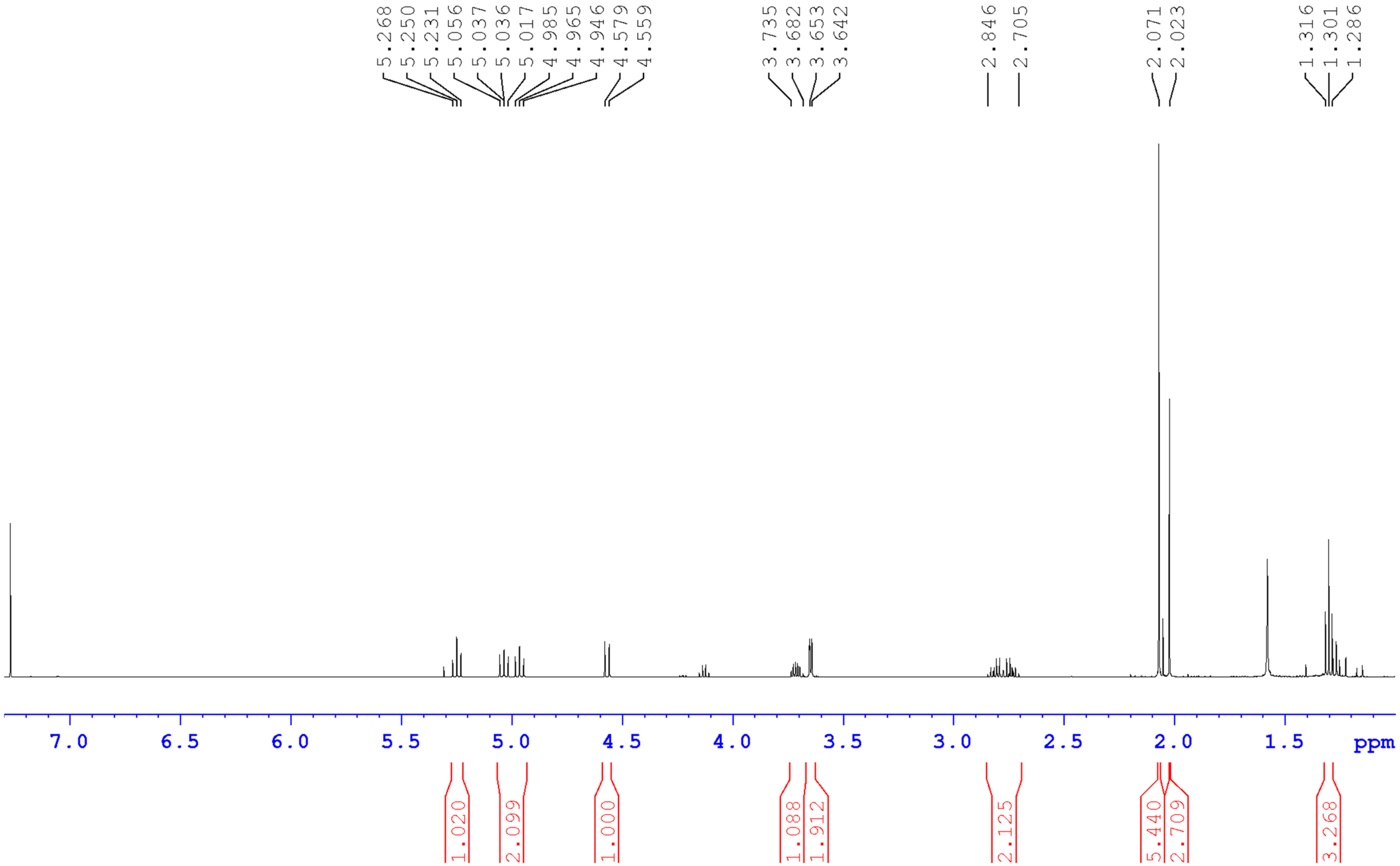

^{13}C-NMR (125 MHz, $CDCl_3$)

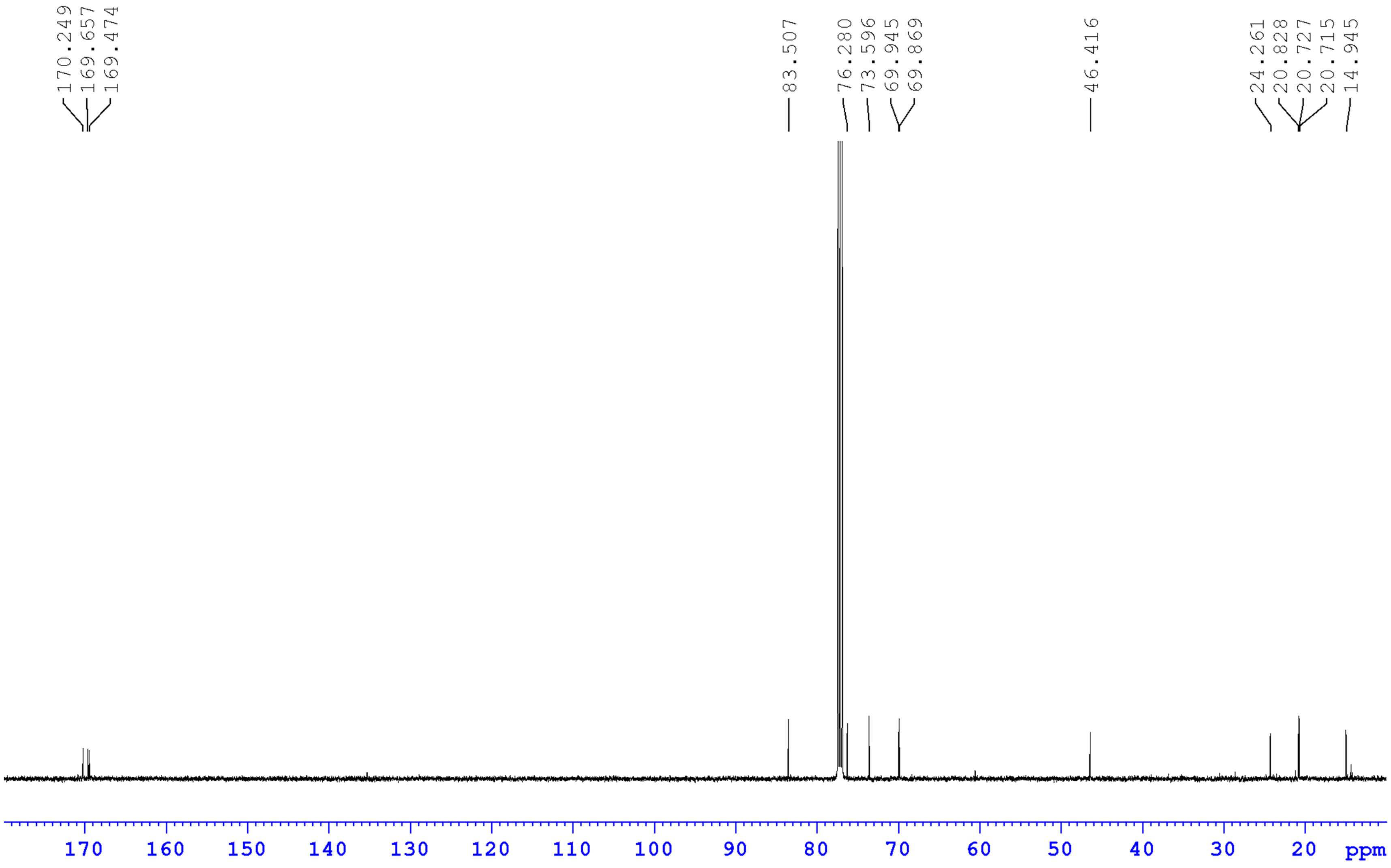

HRMS

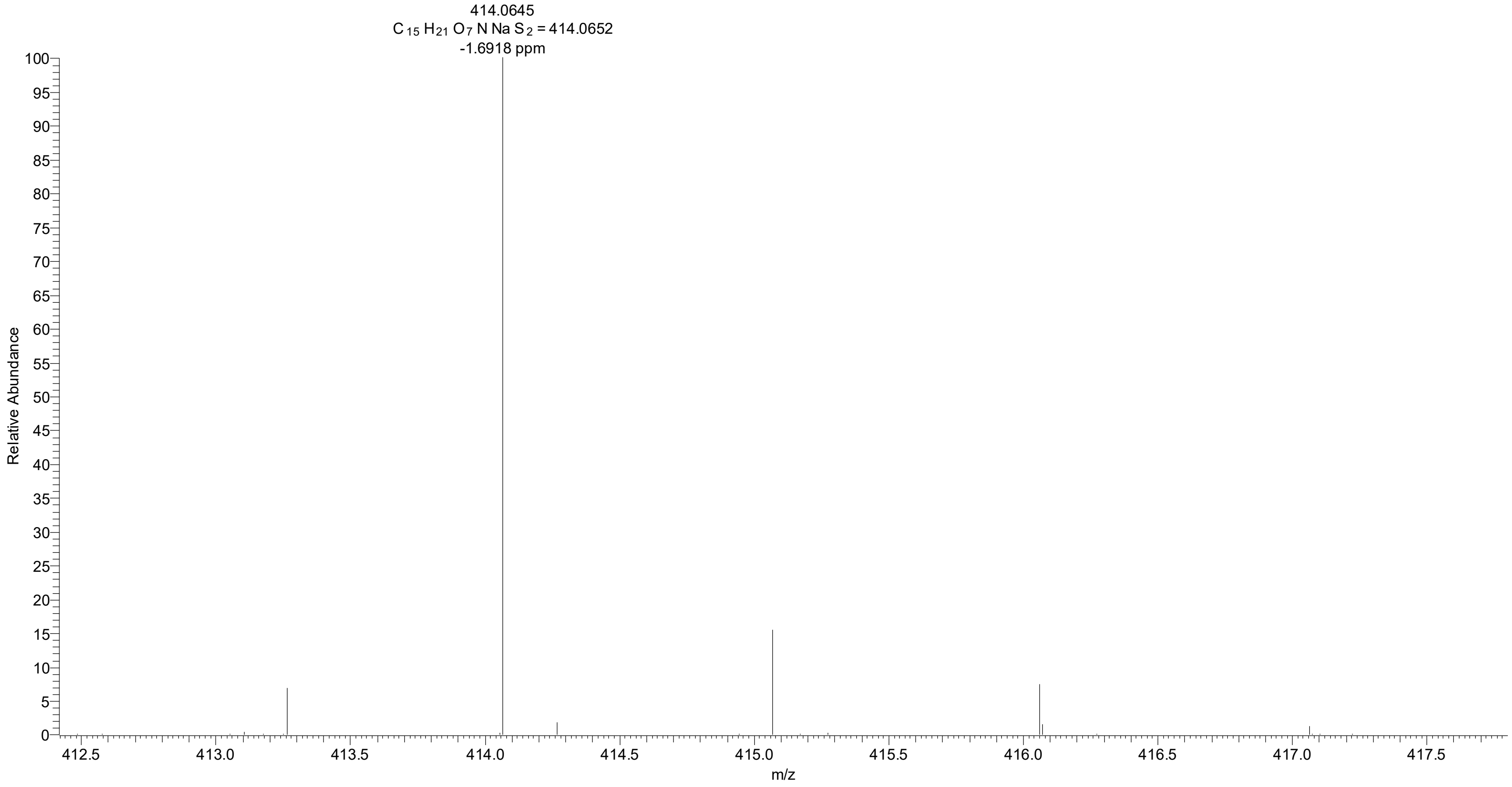

414.0645
C 15 H21 O7 N Na S 2 = 414.0652
-1.6918 ppm
Relative Abundance
m/z

Phenyl 2,3,4-tri-*O*-acetyl-6-isothiocyanato-1,6-dideoxy-1-thio-β-D-glucopyranoside (8)

^{1}H-NMR (500 MHz, $CDCl_3$)

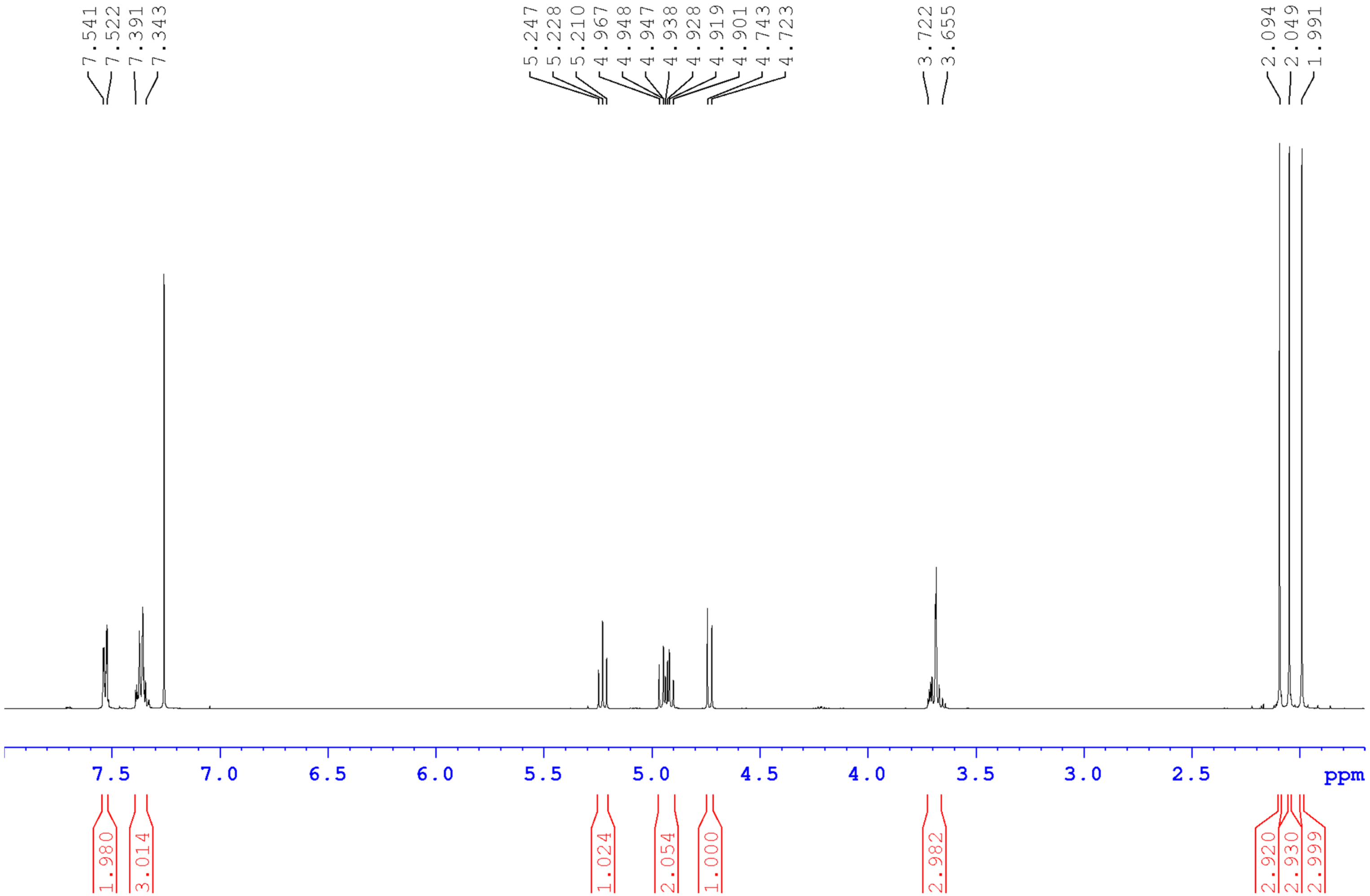

^{13}C-NMR (125 MHz, $CDCl_3$)

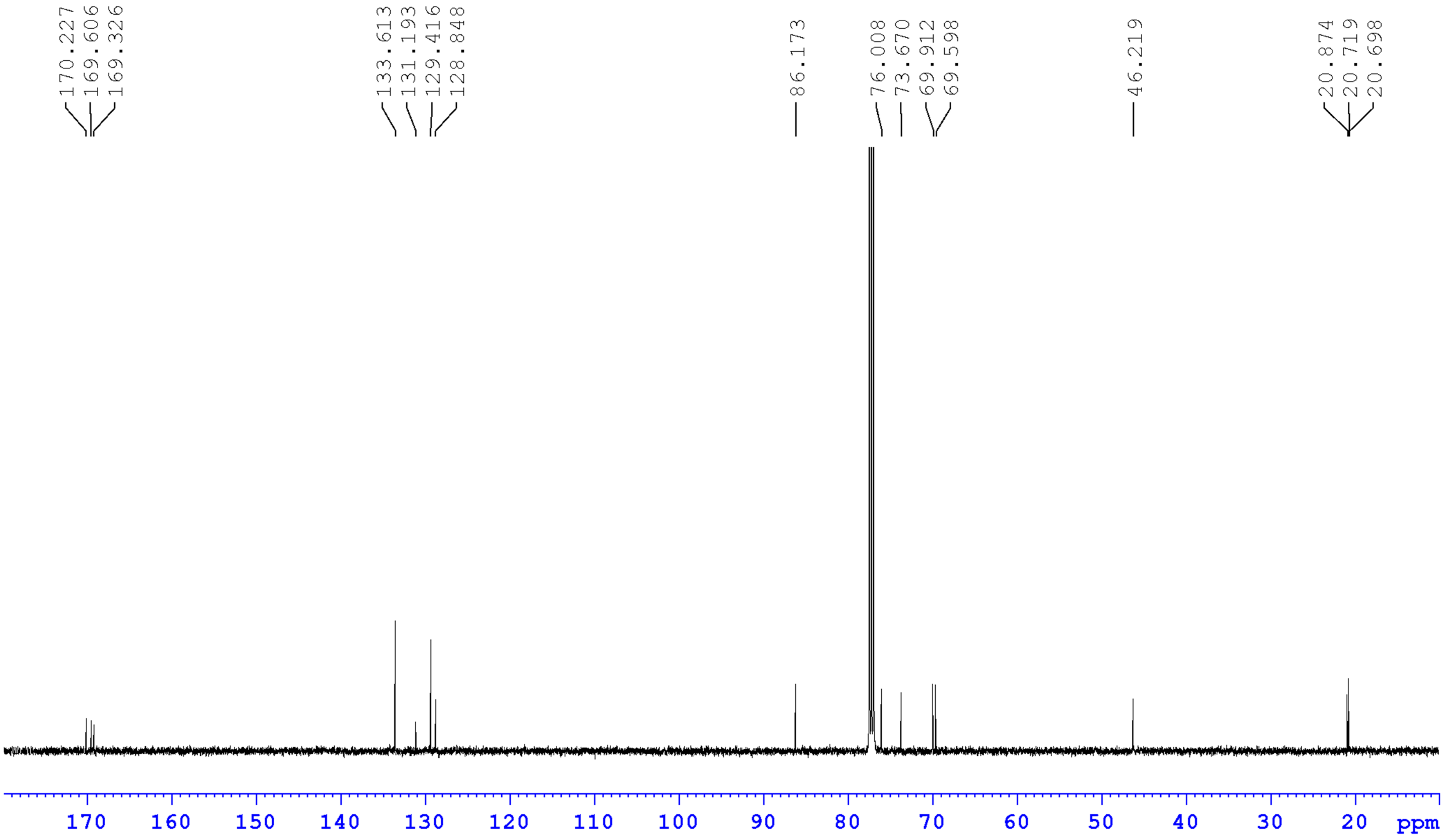

HRMS

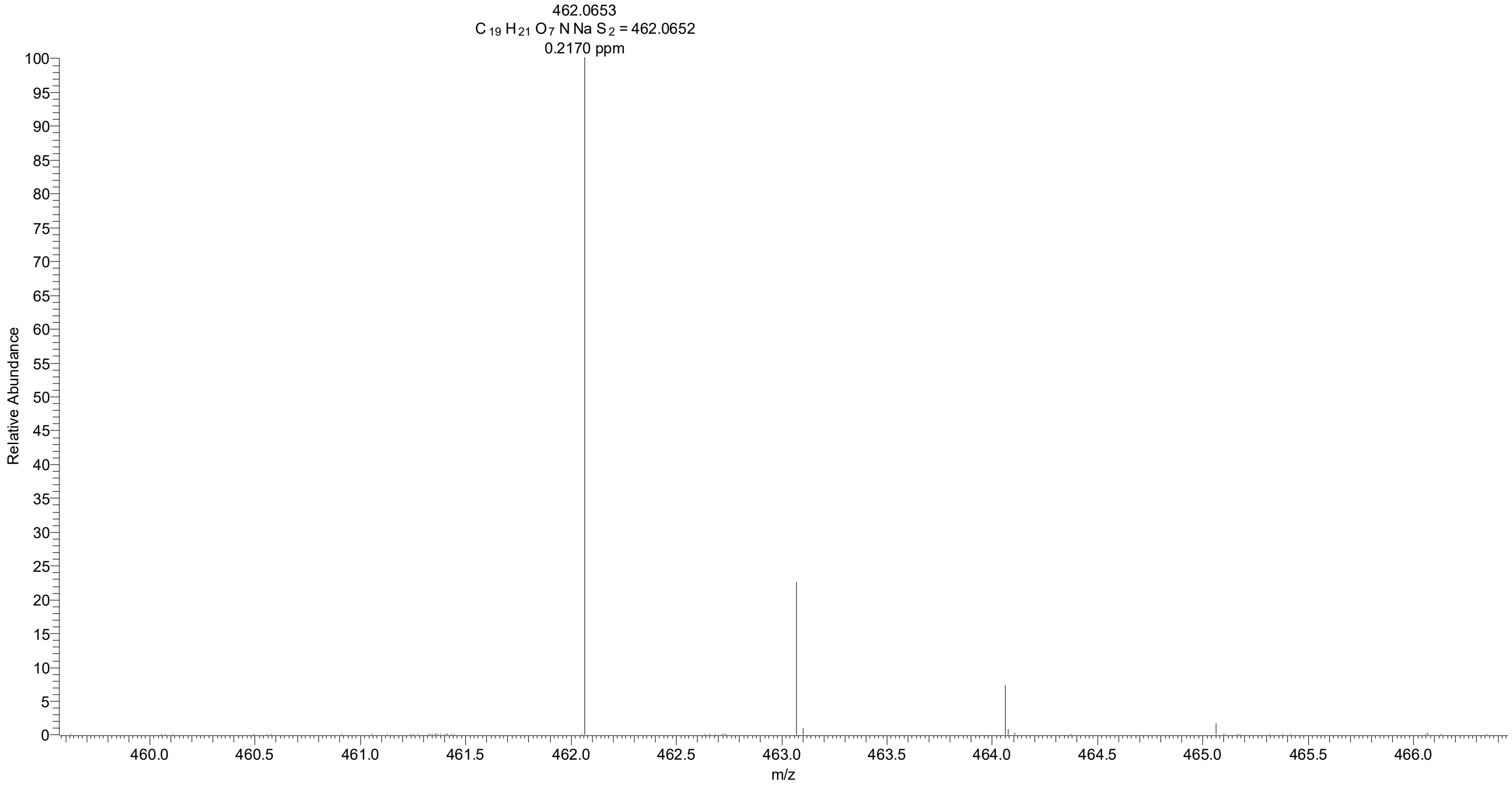
462.0653
C 19 H 21 O 7 N Na S 2 = 462.0652
0.2170 ppm
Relative Abundance
m/z

Phenyl 2,3,4-tri-*O*-acetyl-6-isothiocyanato-1,6-dideoxy-1-thio-α-D-glucopyranoside (9)

^{1}H-NMR (500 MHz, $CDCl_3$)

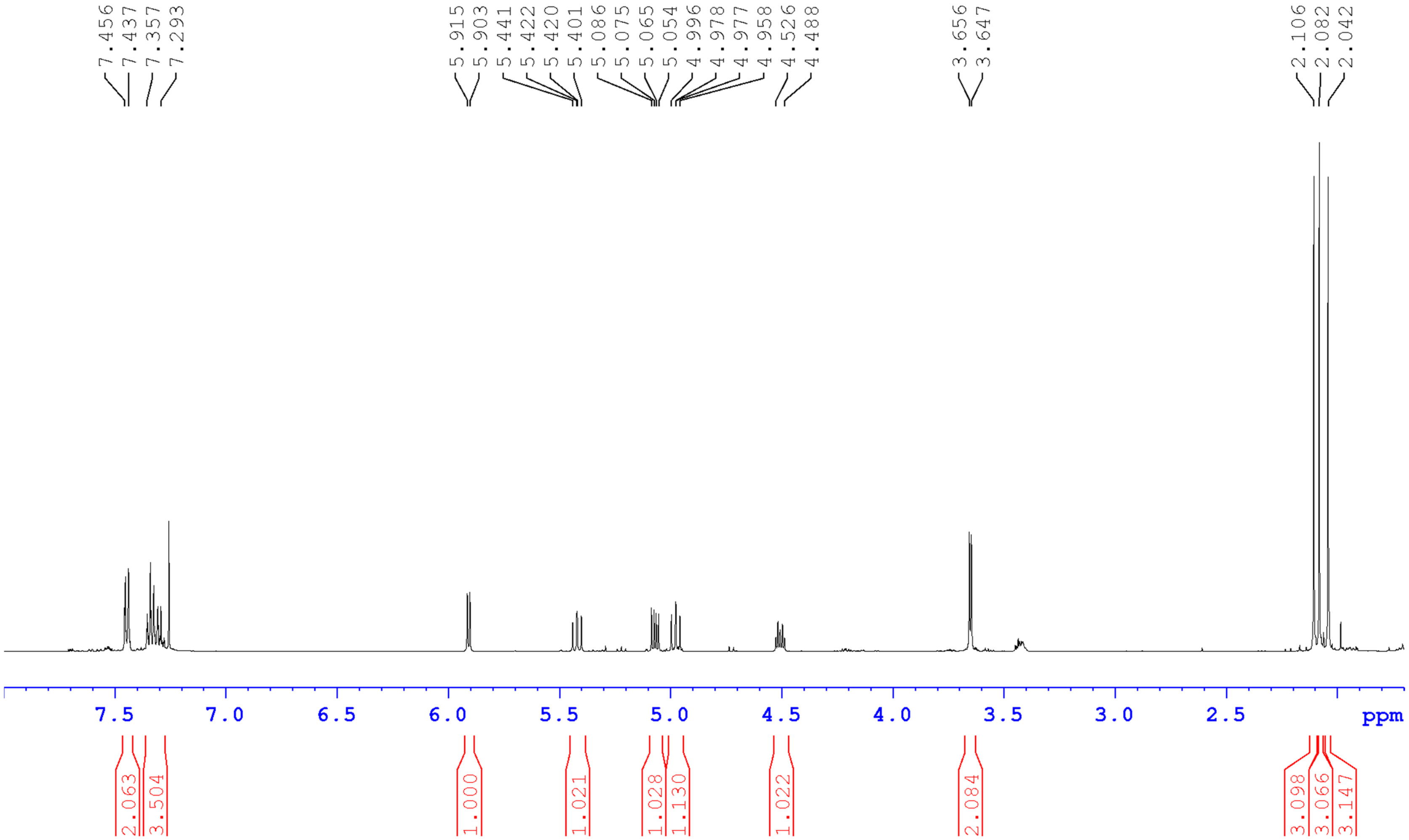

^{13}C-NMR (125 MHz, $CDCl_3$)

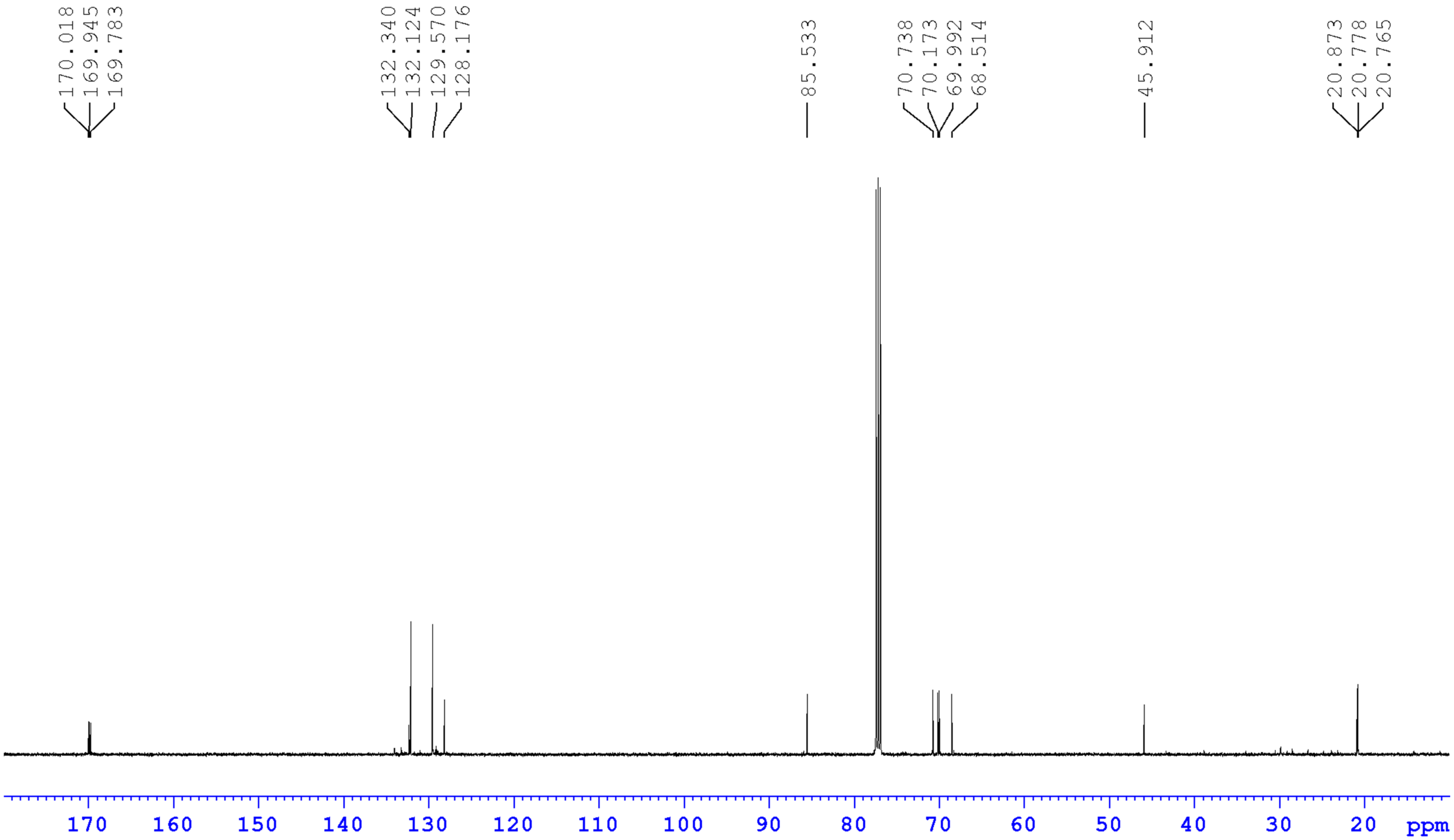

HRMS

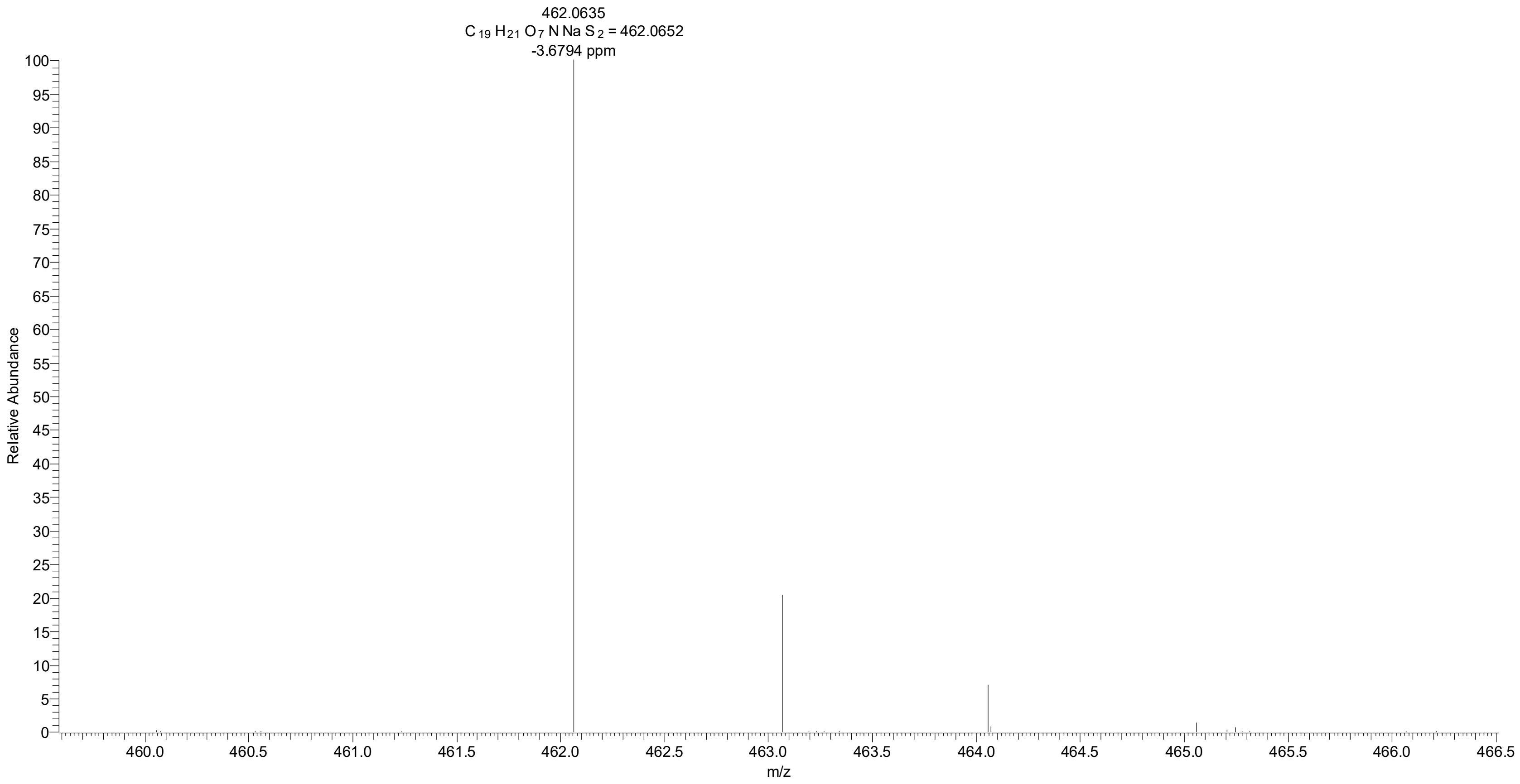
462.0635
C 19 H 21 O 7 N Na S 2 = 462.0652
-3.6794 ppm
Relative Abundance
m/z
100
95
90
85
80
75
70
65
60
55
50
45
40
35
30
25
20
15
10
5
0
460.0
460.5
461.0
461.5
462.0
462.5
463.0
463.5
464.0
464.5
465.0
465.5
466.0
466.5

2,3,4-tri-*O*-acetyl-6-isothiocyanato-1,6-dideoxy-1[(*S*)-ethylsulfinyl]-β-D-glucopyranose ((*S*)-10)

^{1}H-NMR (500 MHz, $CDCl_3$)

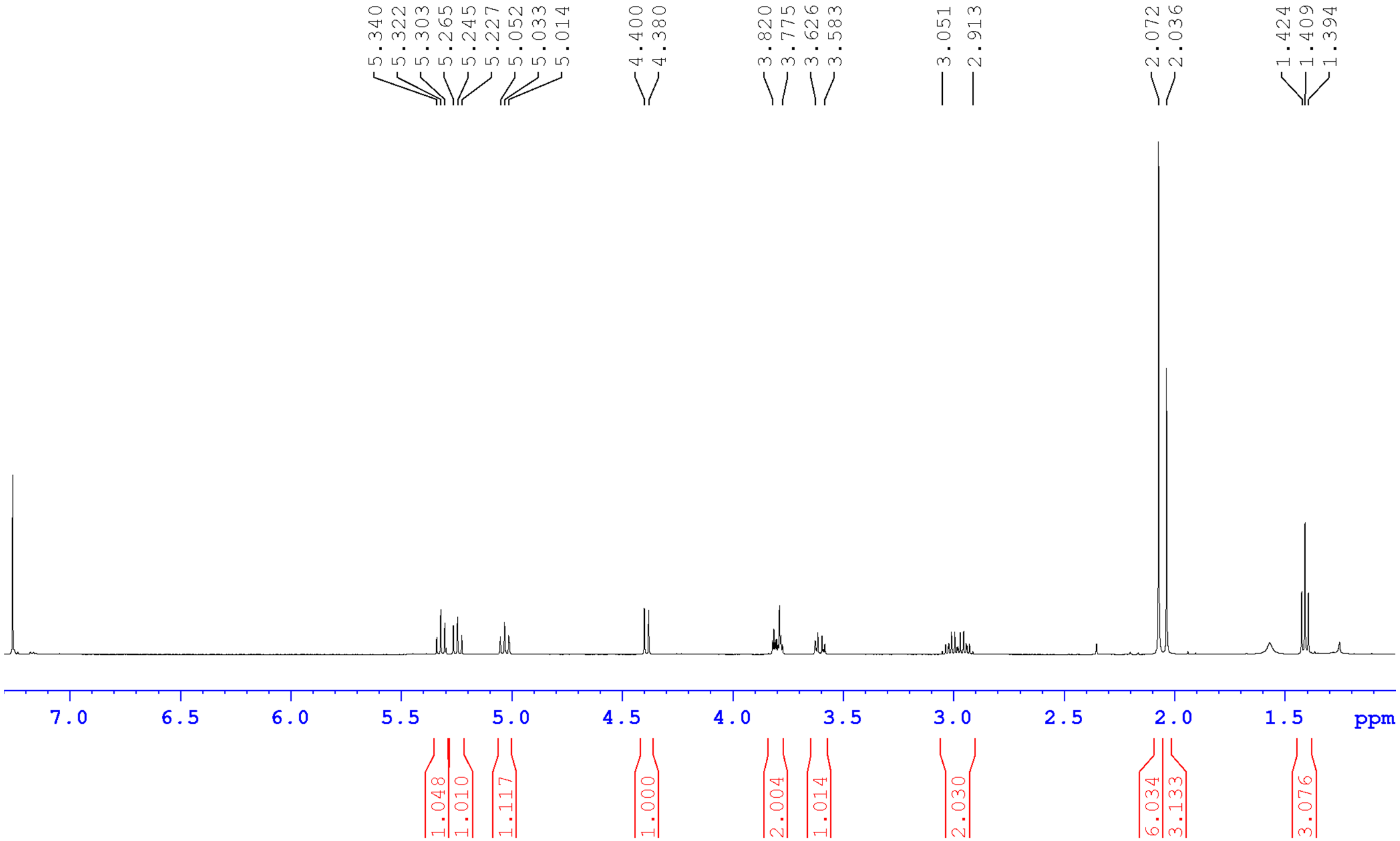

^{13}C-NMR (125 MHz, $CDCl_3$)

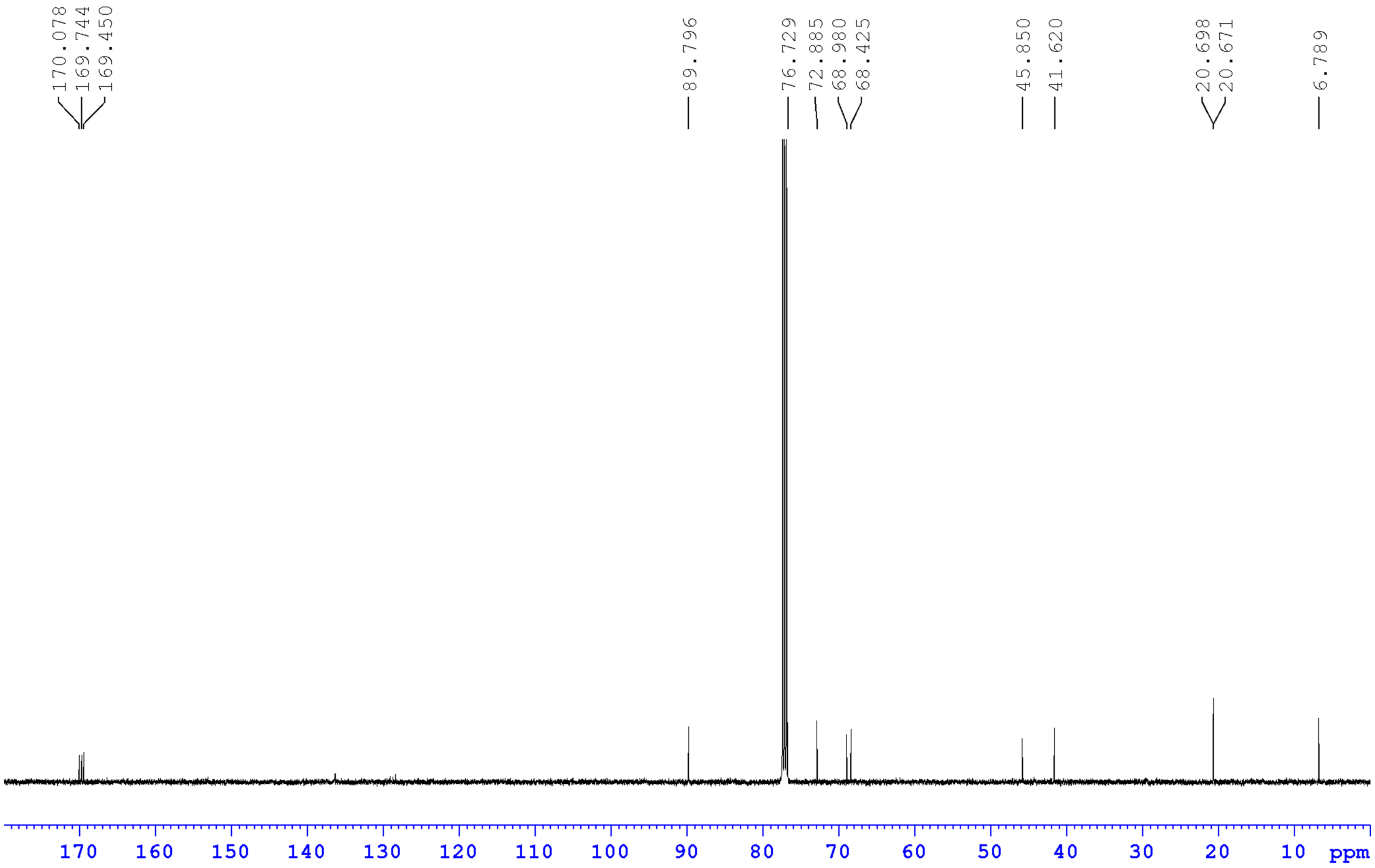

HRMS

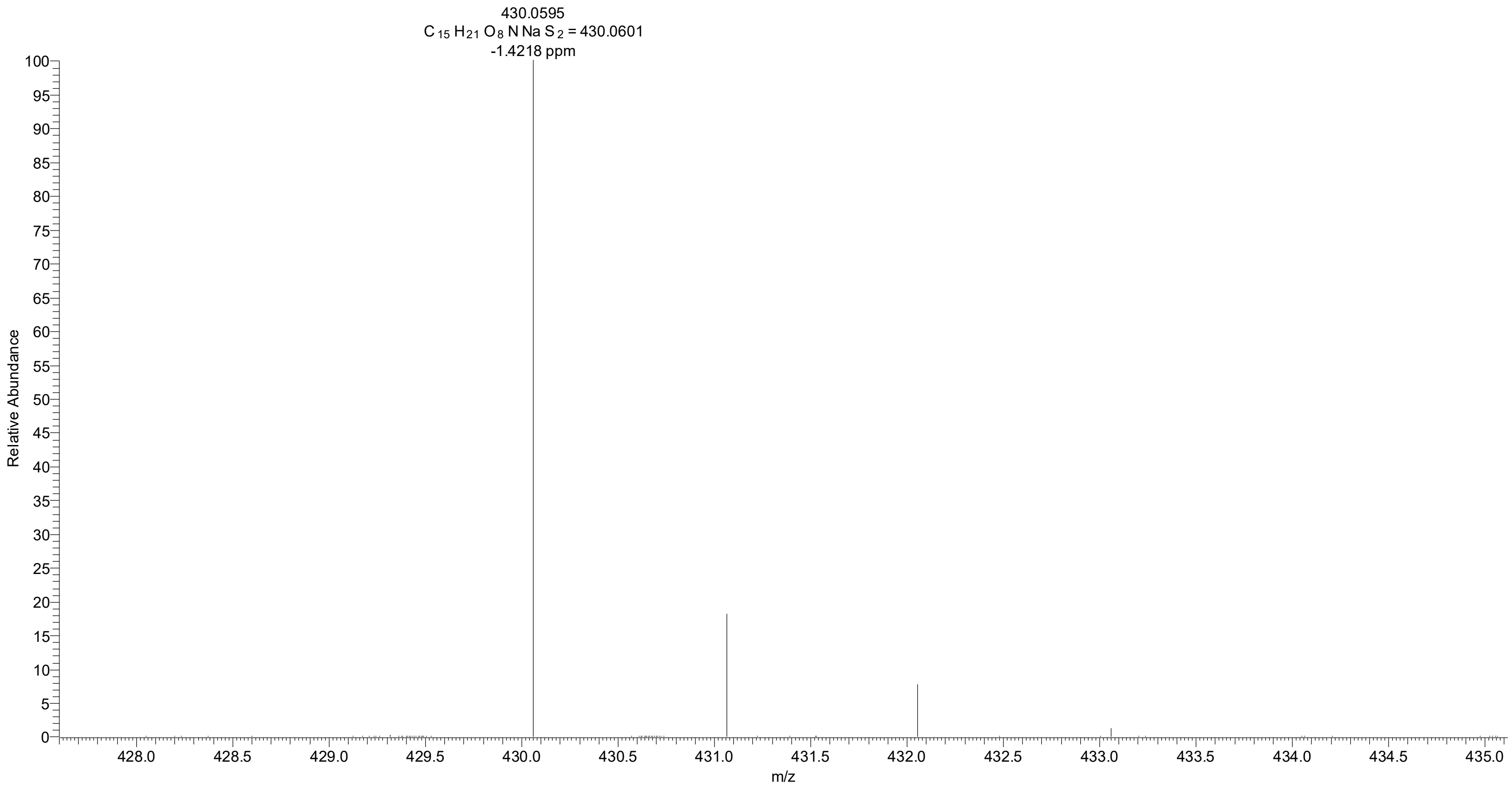
430.0595
C 15 H 21 O 8 N Na S 2 = 430.0601
-1.4218 ppm
Relative Abundance
m/z

2,3,4-tri-*O*-acetyl-6-isothiocyanato-1,6-dideoxy-1[(*R*)-ethylsulfinyl]-β-D-glucopyranose ((*R*)-10)

^{1}H-NMR (500 MHz, $CDCl_3$)

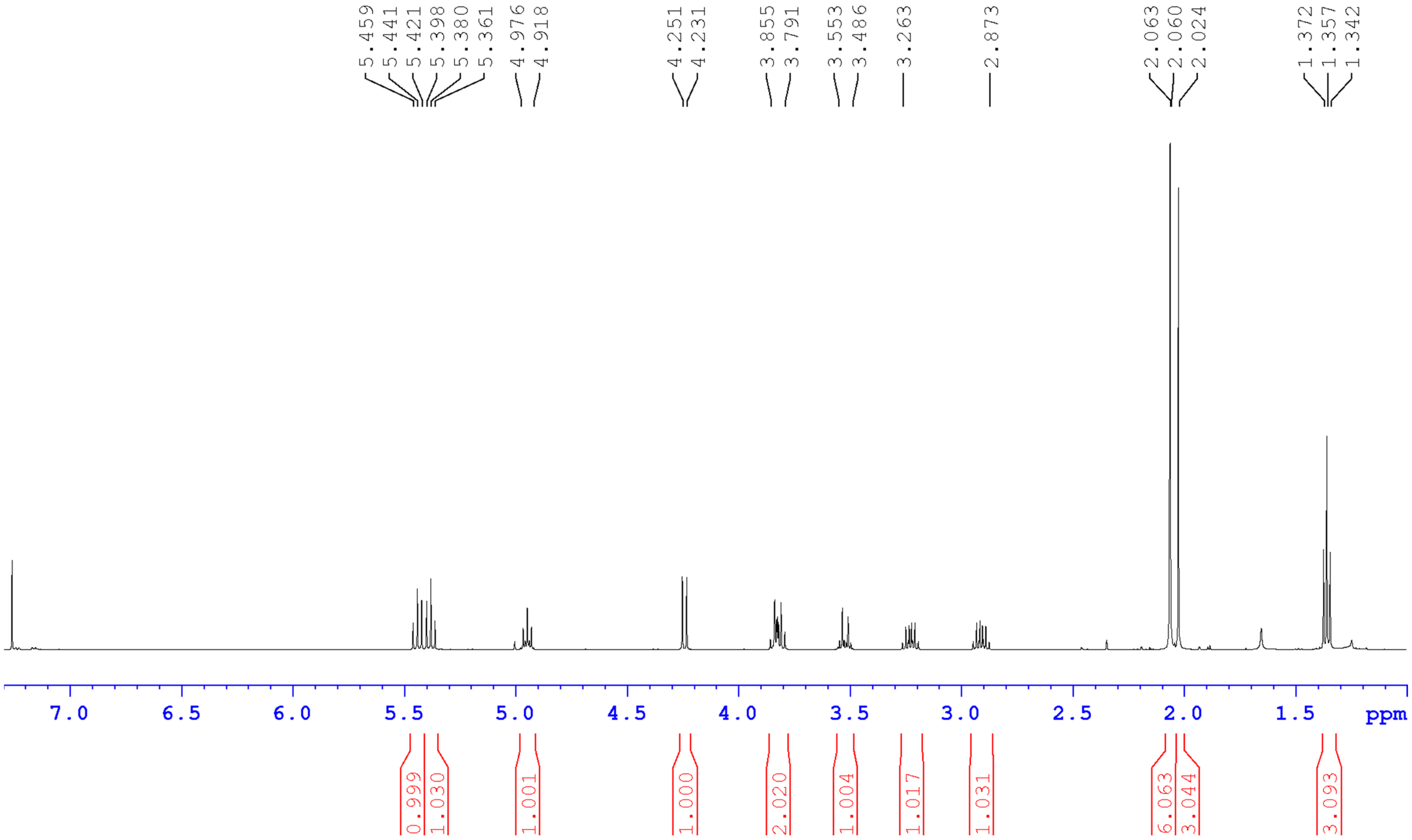

^{13}C-NMR (125 MHz, $CDCl_3$)

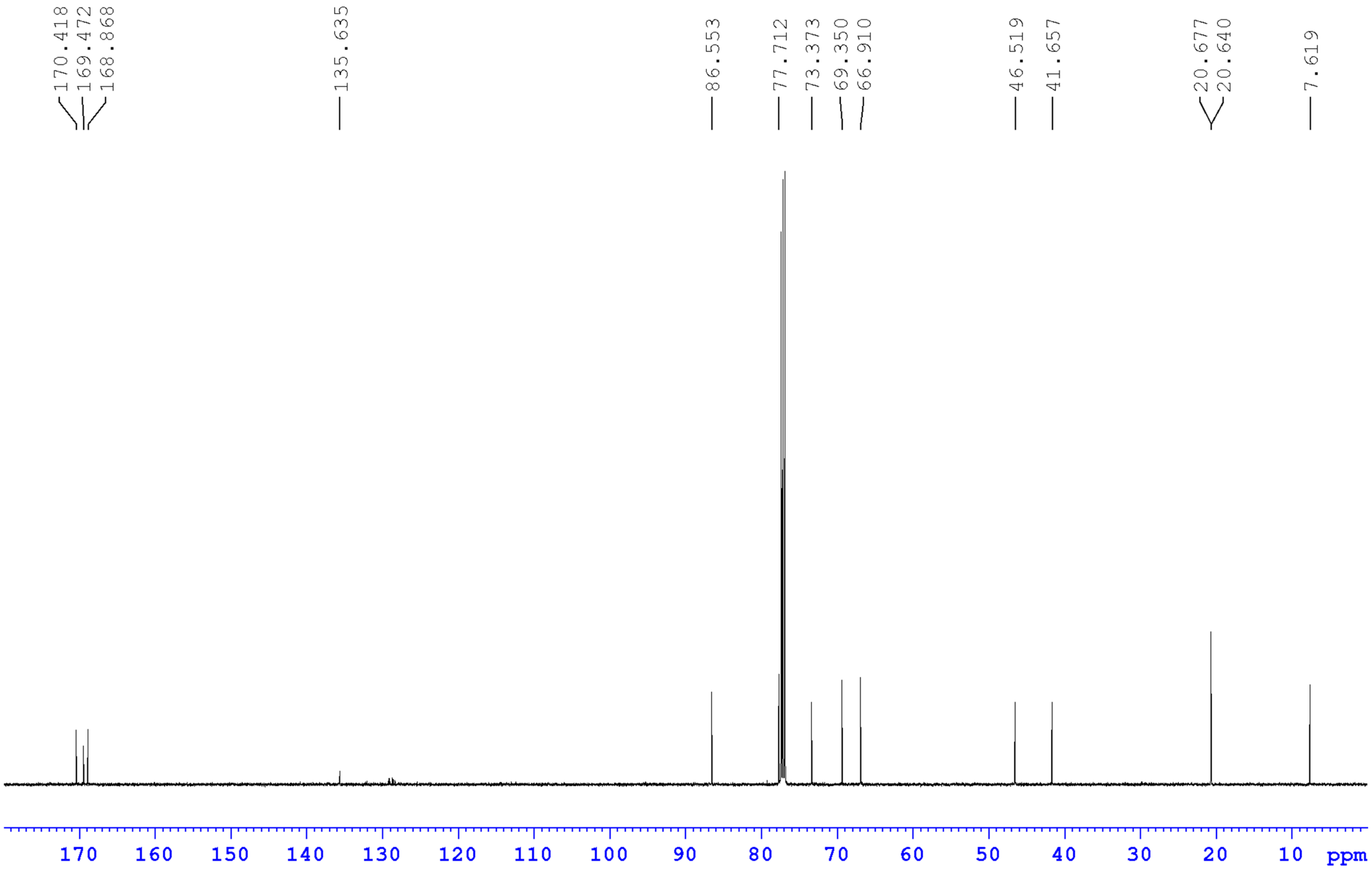

HRMS

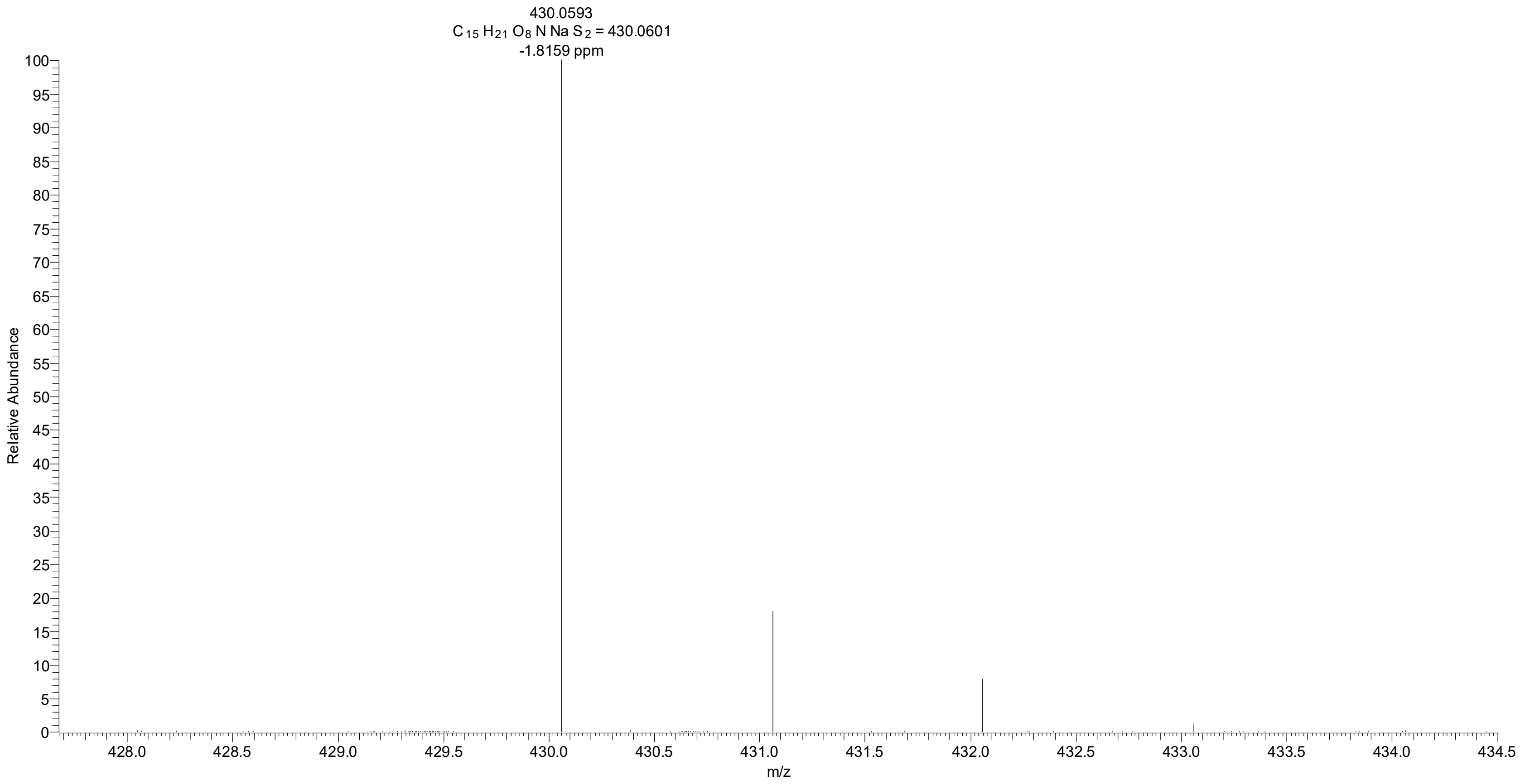
430.0593
C 15 H 21 O 8 N Na S 2 = 430.0601
-1.8159 ppm
Relative Abundance
m/z

2,3,4-tri-*O*-acetyl-6-isothiocyanato-1,6-dideoxy-1[(*S*)-phenylsulfinyl]-β-D-glucopyranose ((*S*)-11)

^{1}H-NMR (500 MHz, $CDCl_3$)

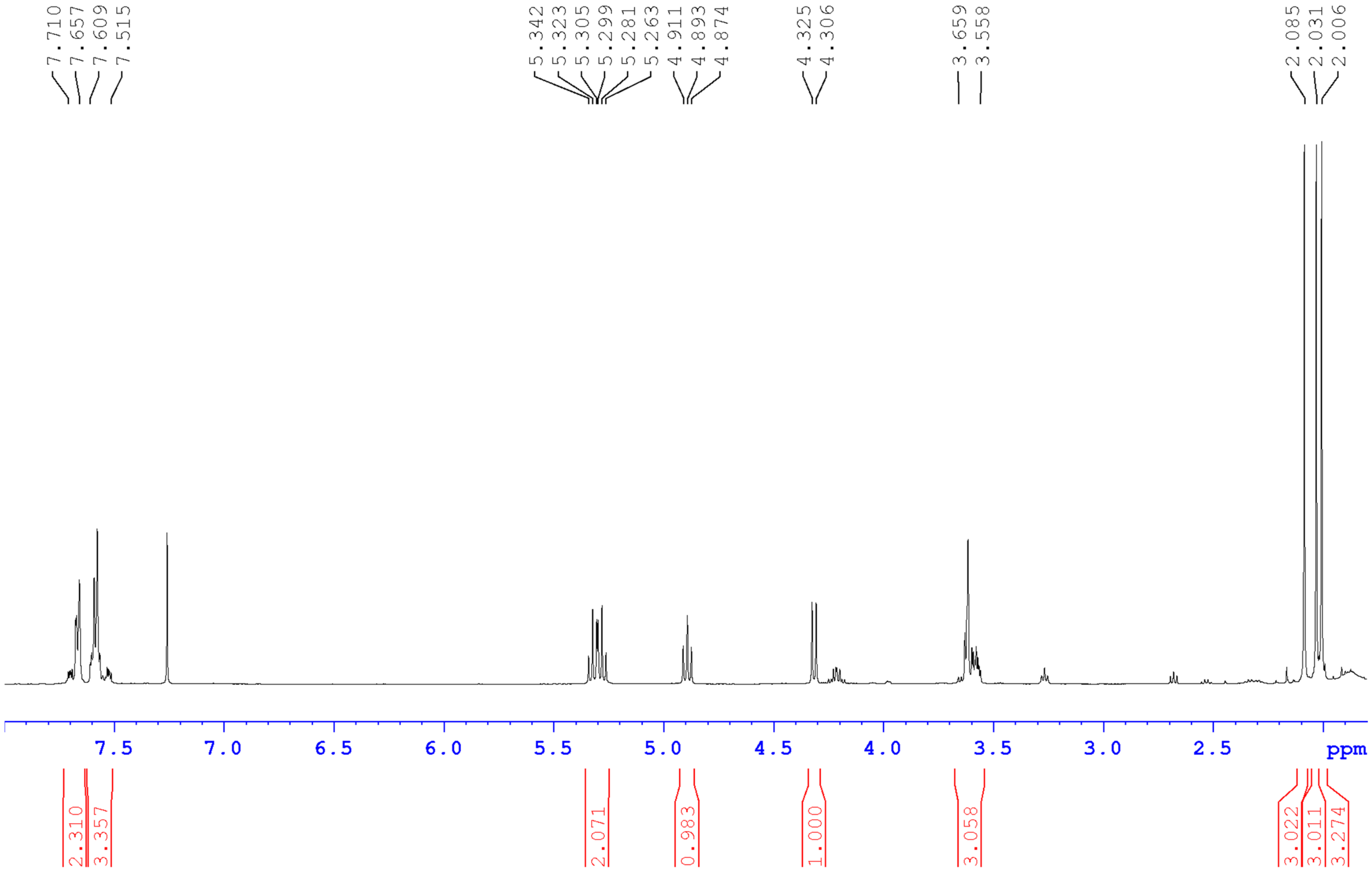

^{13}C-NMR (125 MHz, $CDCl_3$)

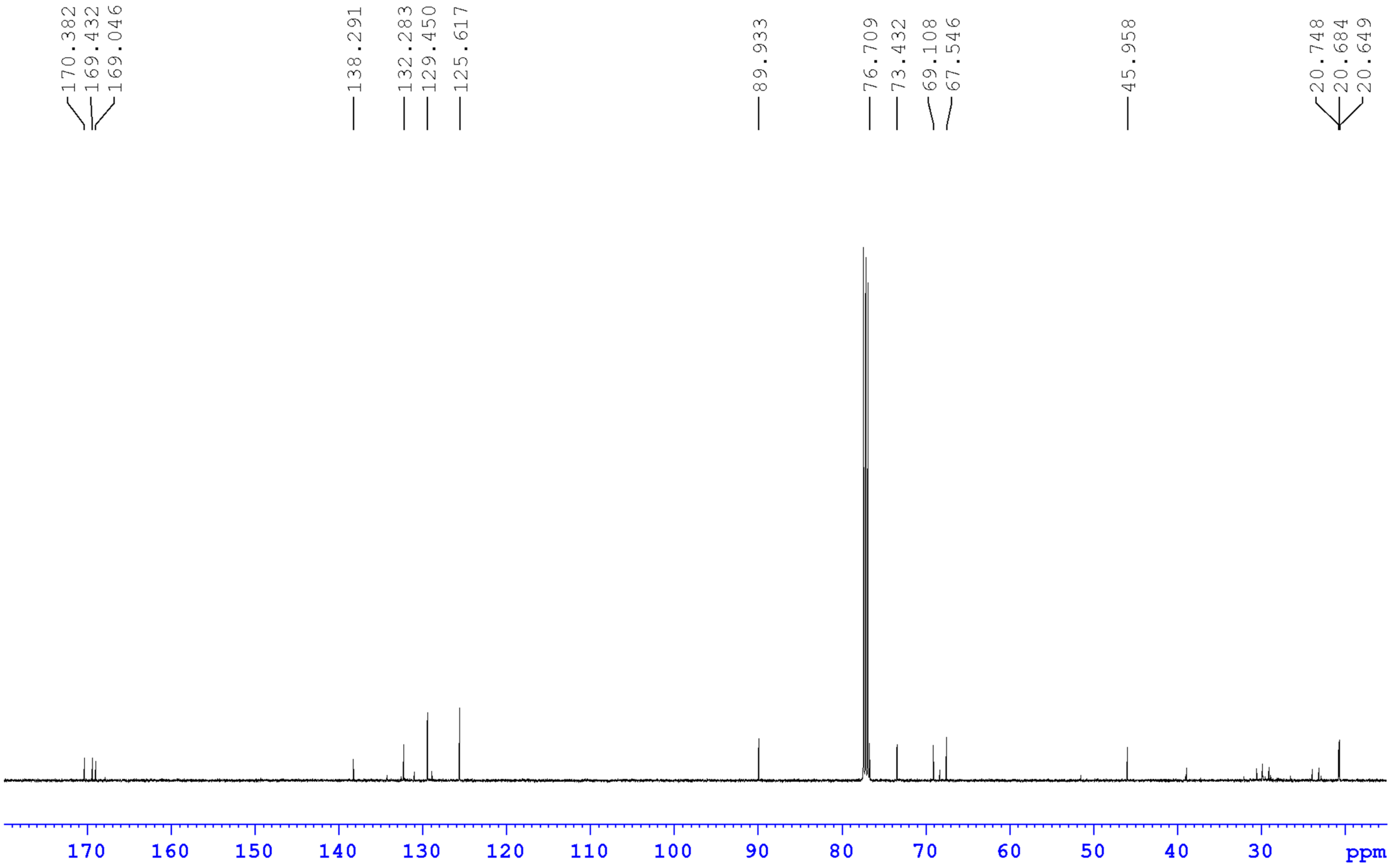

HRMS

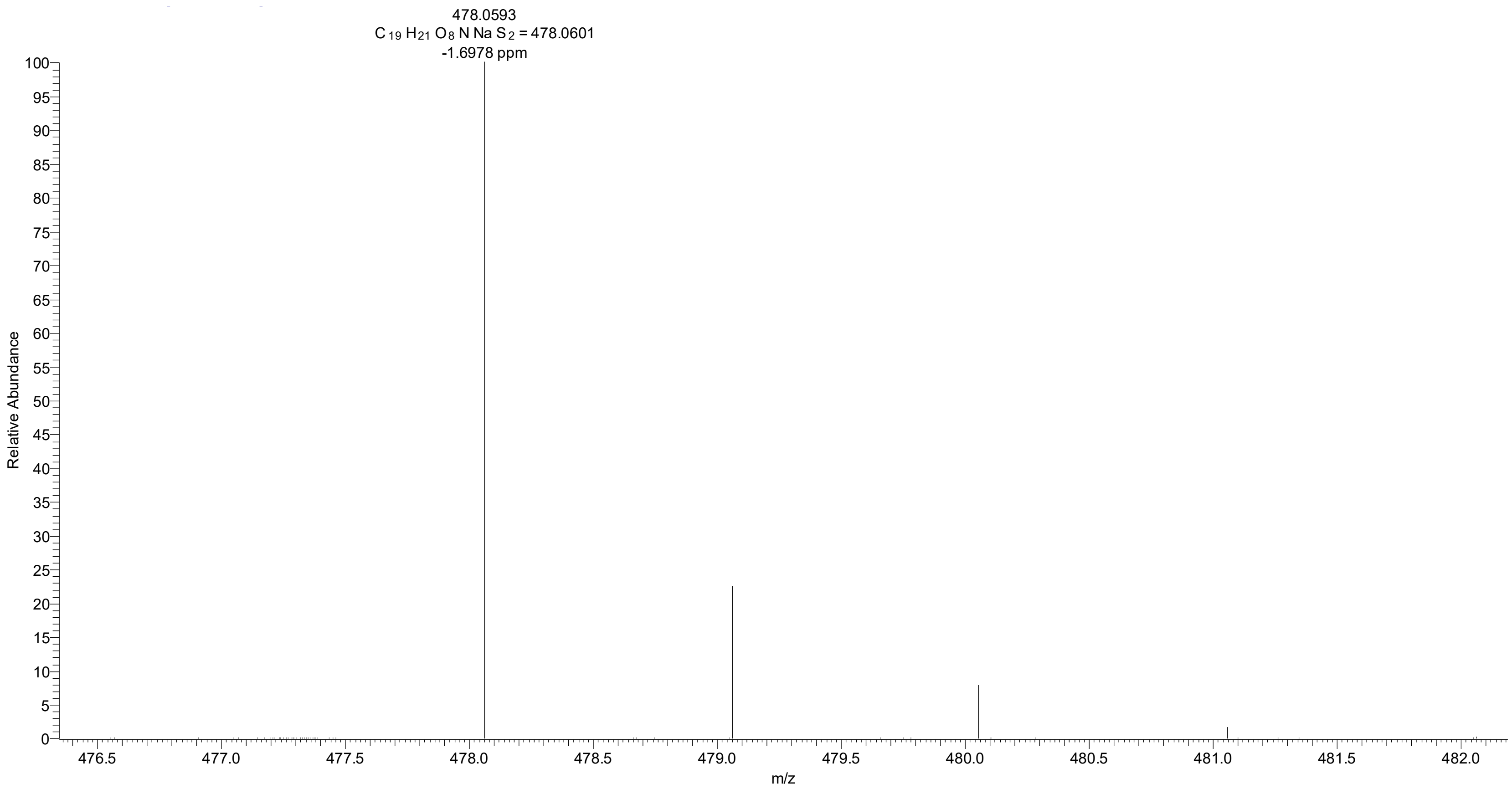
478.0593
C 19 H 21 O 8 N Na S 2 = 478.0601
-1.6978 ppm
Relative Abundance
m/z
0
5
10
15
20
25
30
35
40
45
50
55
60
65
70
75
80
85
90
95
100
476.5
477.0
477.5
478.0
478.5
479.0
479.5
480.0
480.5
481.0
481.5
482.0

2,3,4-tri-*O*-acetyl-6-isothiocyanato-1,6-dideoxy-1[(*R*)-phenylsulfinyl]-β-D-glucopyranose ((*R*)-11)

^{1}H-NMR (500 MHz, $CDCl_3$)

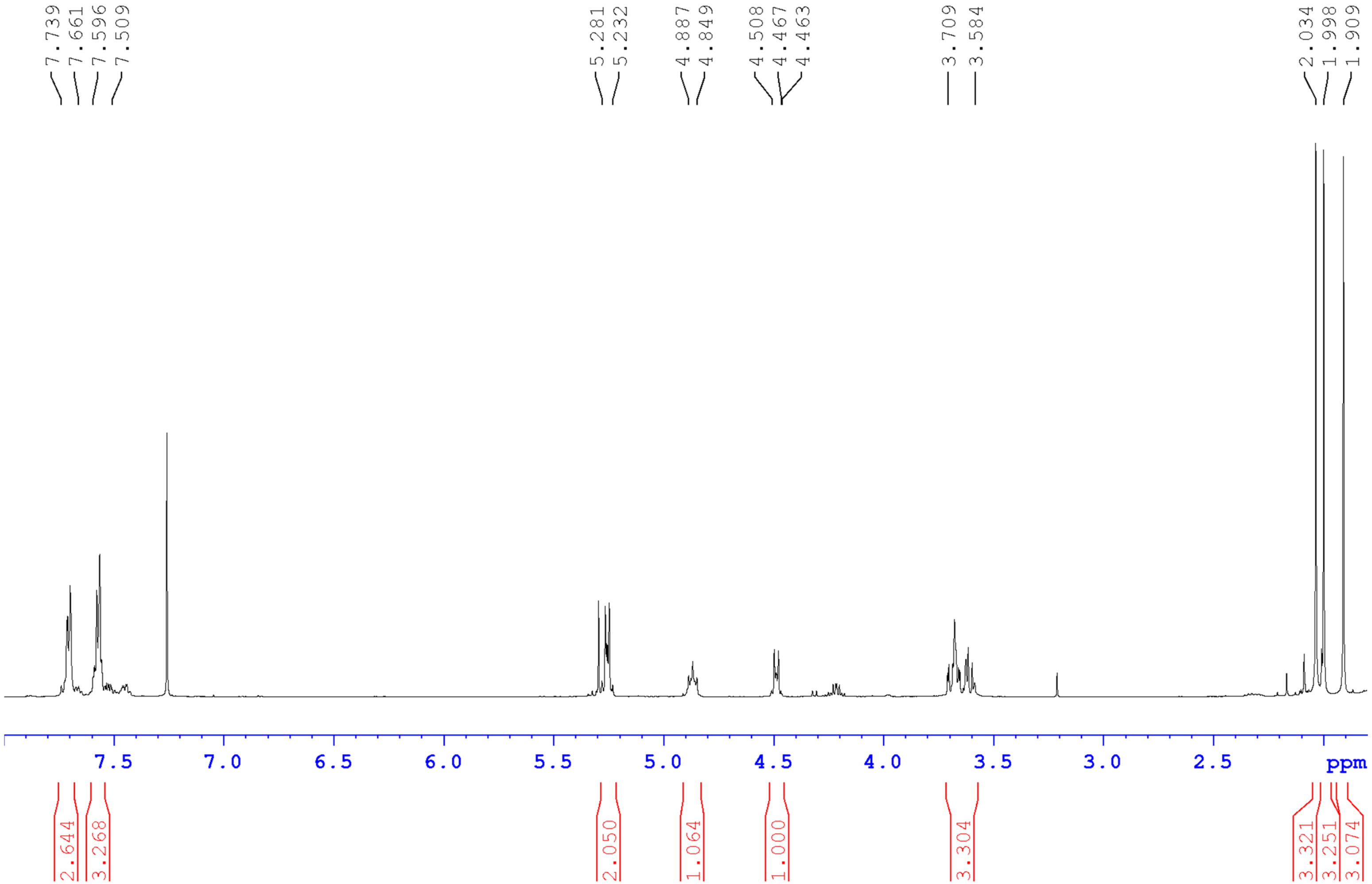

^{13}C-NMR (125 MHz, $CDCl_3$)

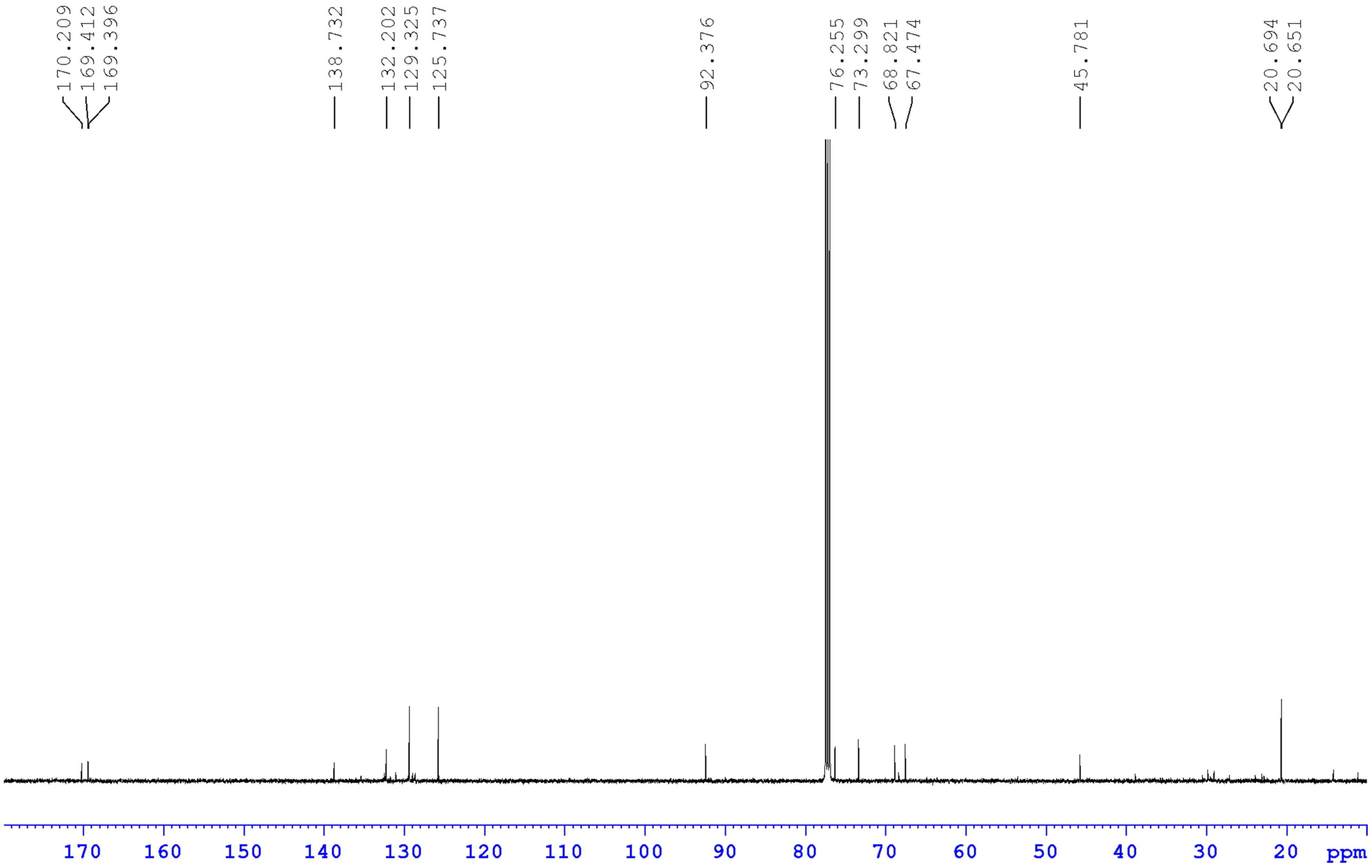

HRMS

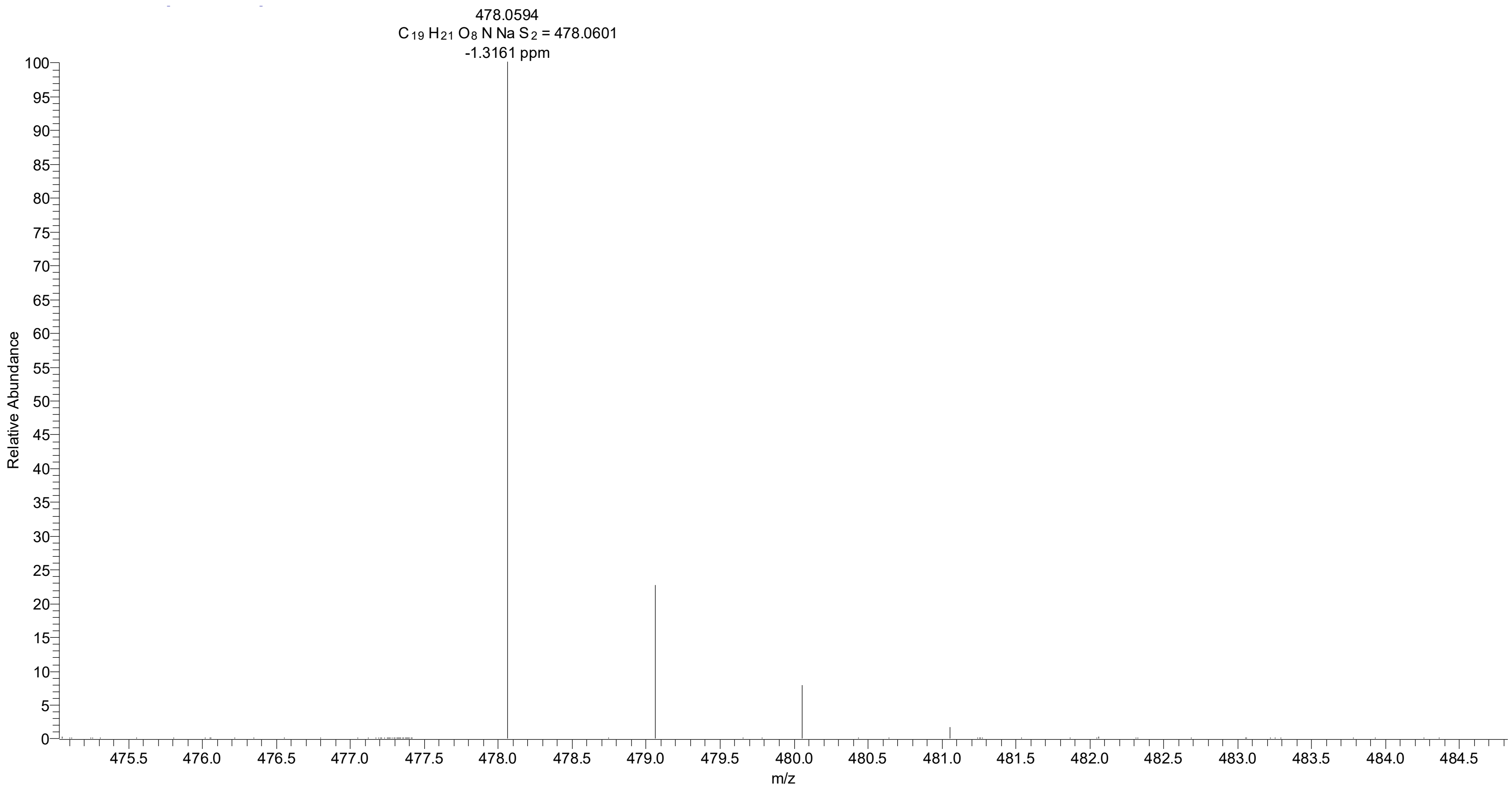

478.0594
C 19 H 21 O 8 N Na S 2 = 478.0601
-1.3161 ppm
Relative Abundance
m/z

2,3,4-tri-*O*-acetyl-6-isothiocyanato-1,6-dideoxy-1-ethylsulfonyl-β-D-glucopyranose (12)

^{1}H-NMR (500 MHz, $CDCl_3$)

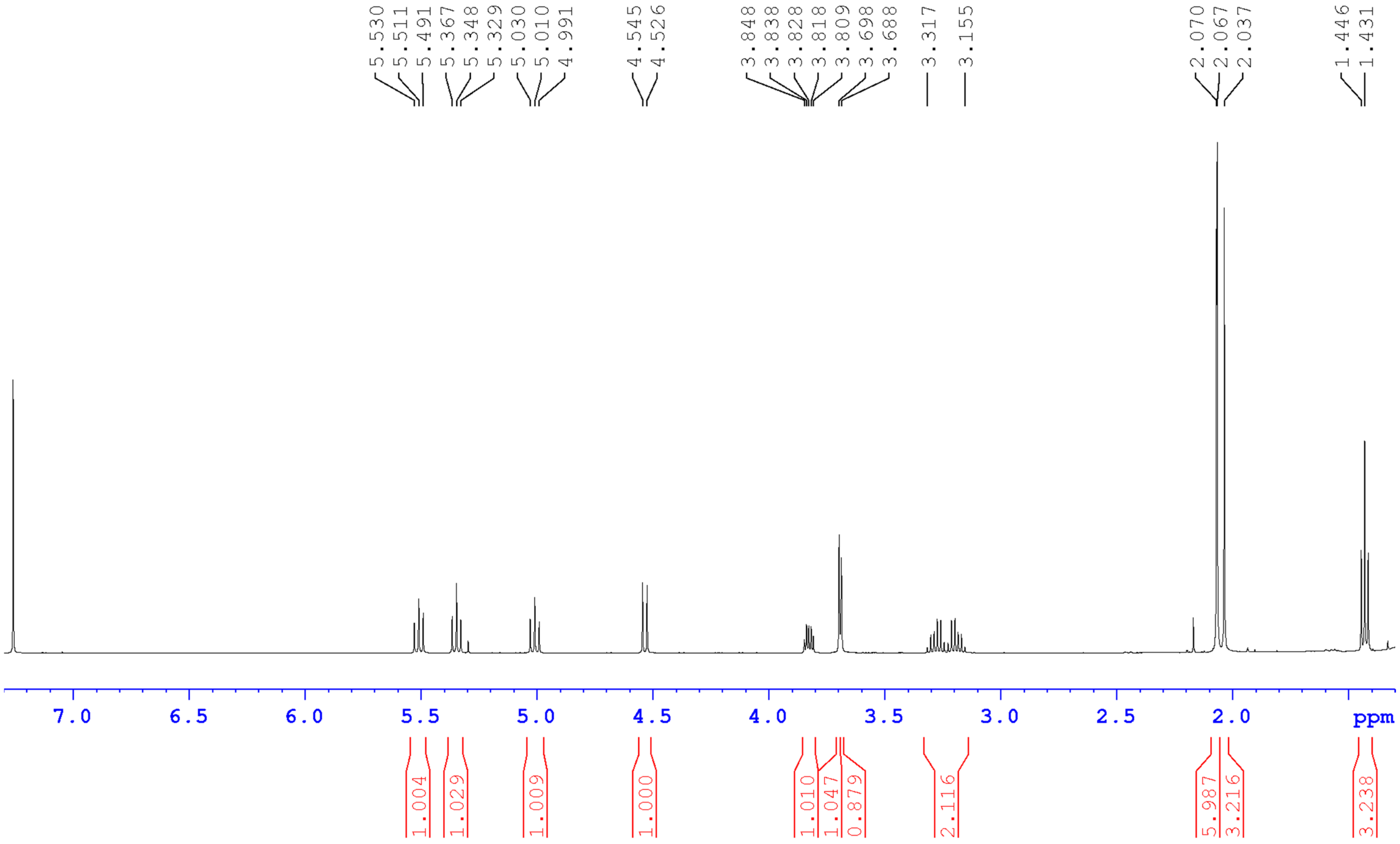

^{13}C-NMR (125 MHz, $CDCl_3$)

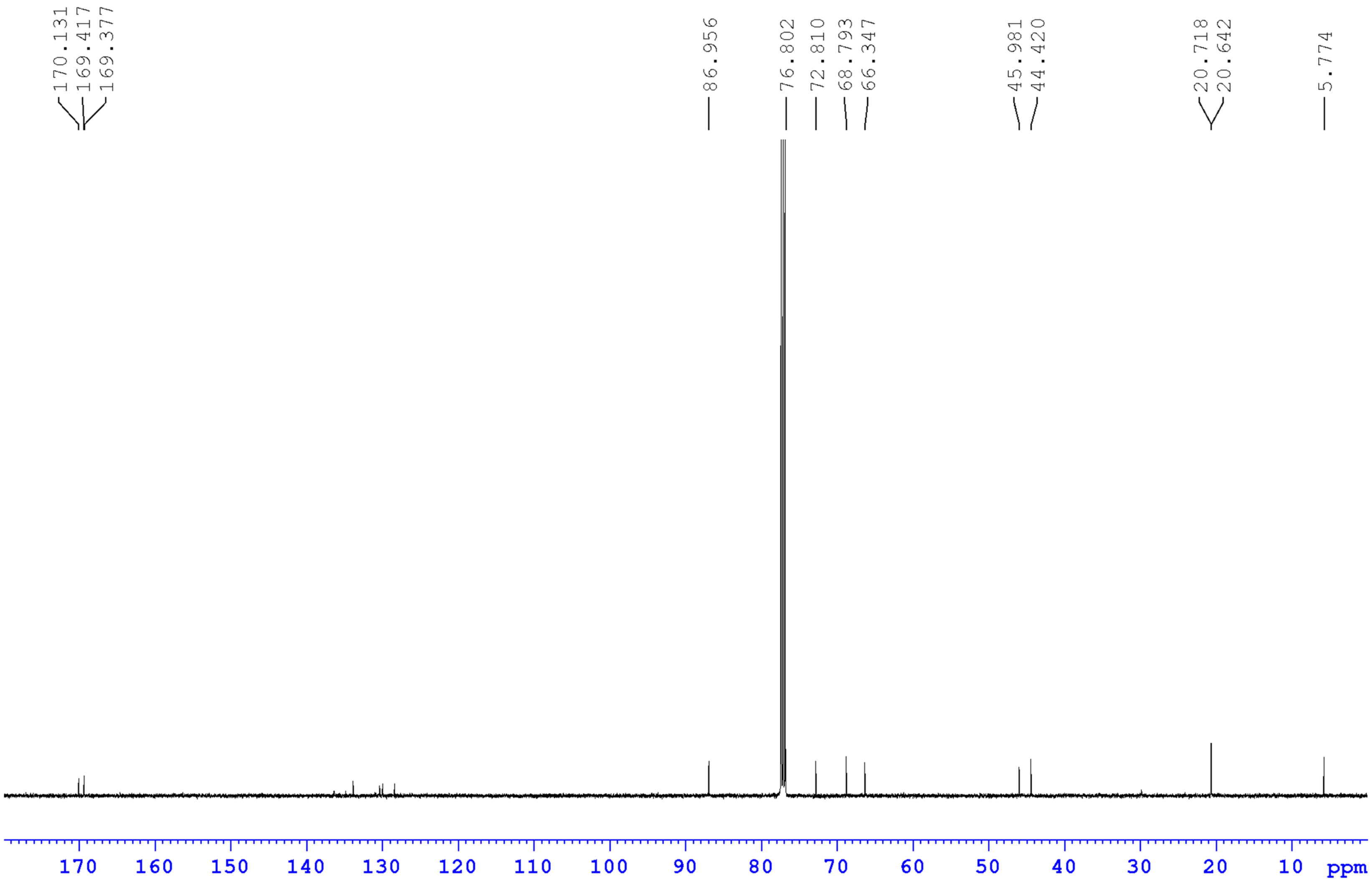

HRMS

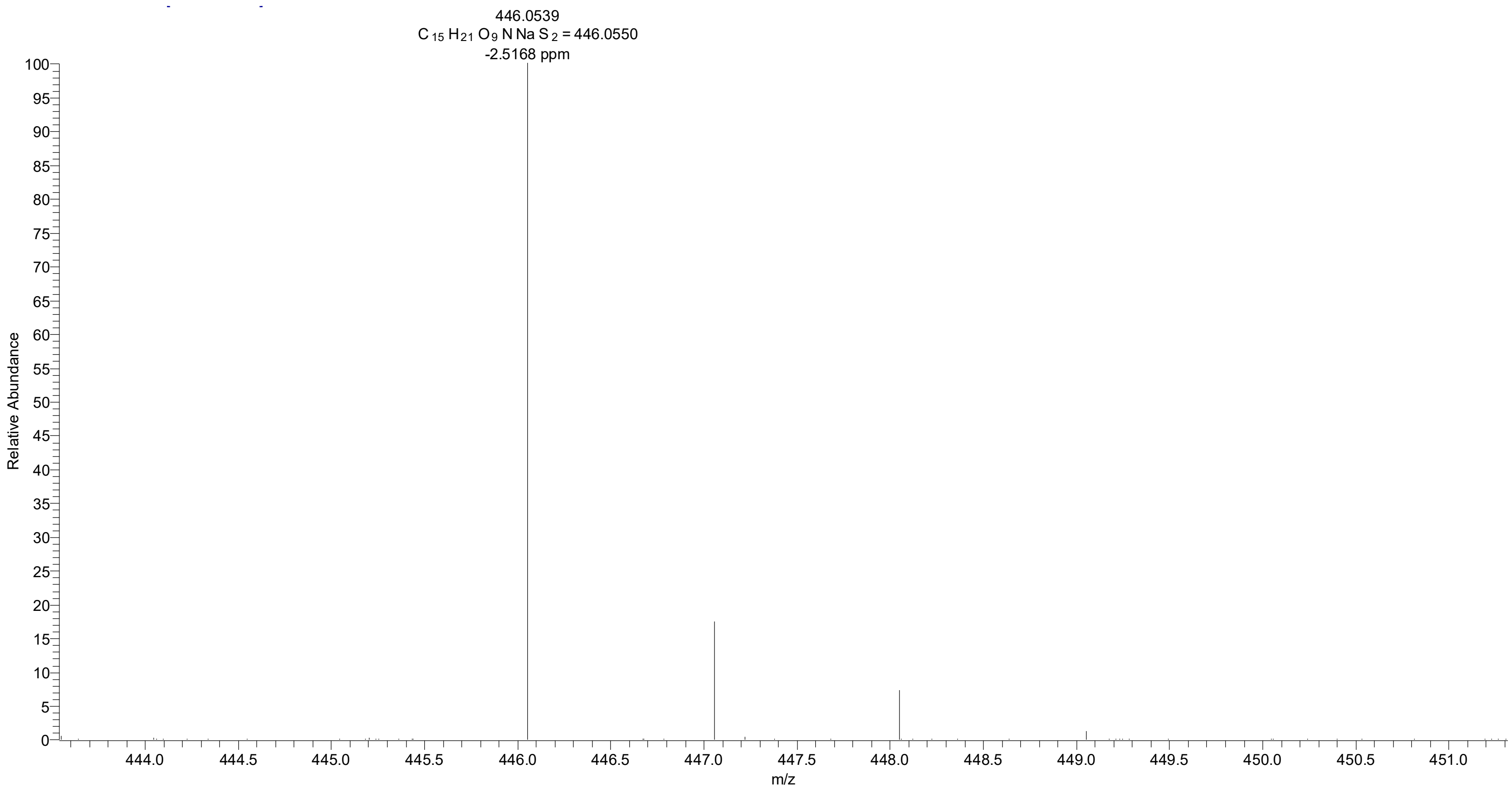
446.0539
C 15 H 21 O 9 N Na S 2 = 446.0550
-2.5168 ppm
Relative Abundance
m/z

2,3,4-tri-*O*-acetyl-6-isothiocyanato-1,6-dideoxy-1-phenylsulfonyl-β-D-glucopyranose (13)

^{1}H-NMR (500 MHz, $CDCl_3$)

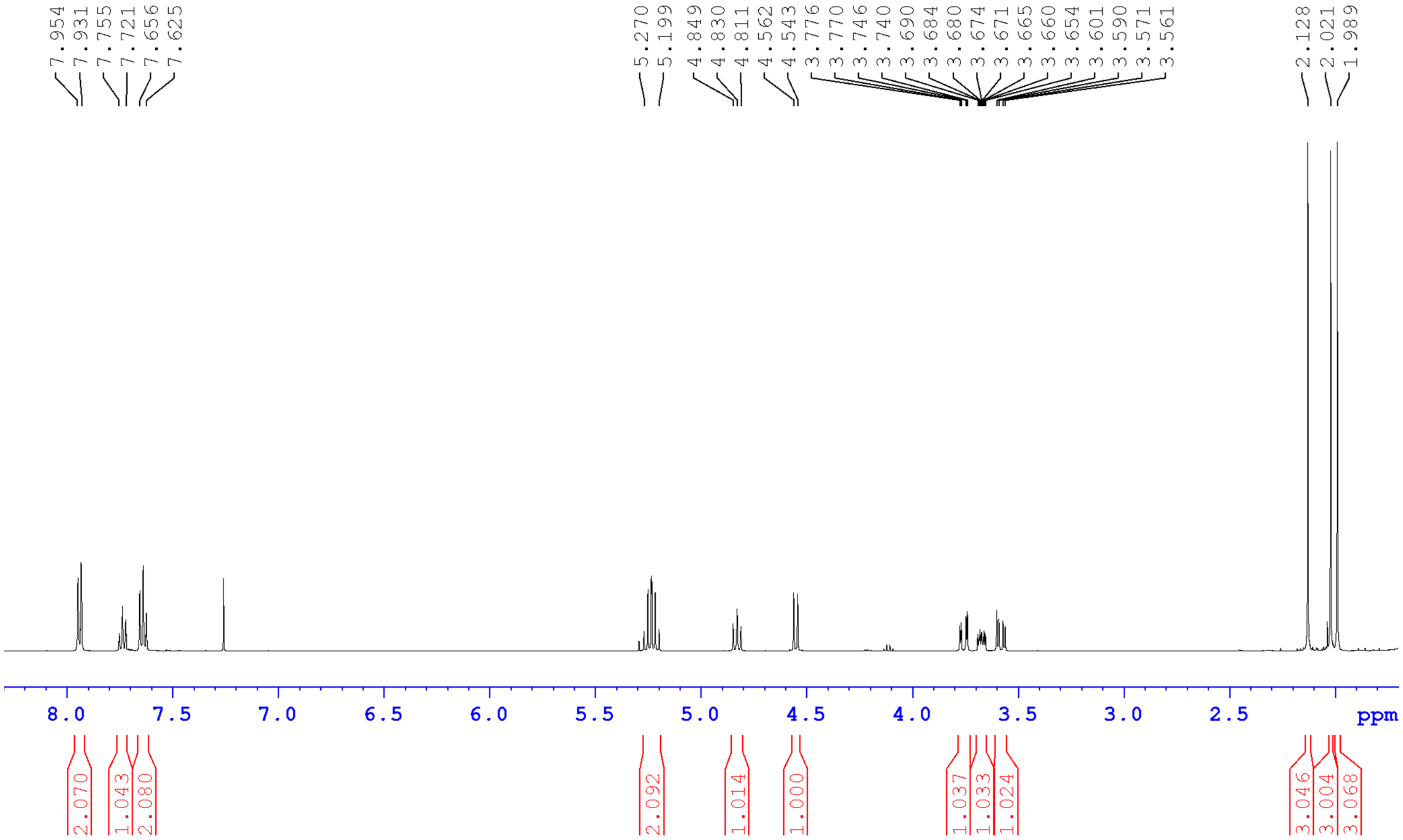

^{13}C-NMR (125 MHz, $CDCl_3$)

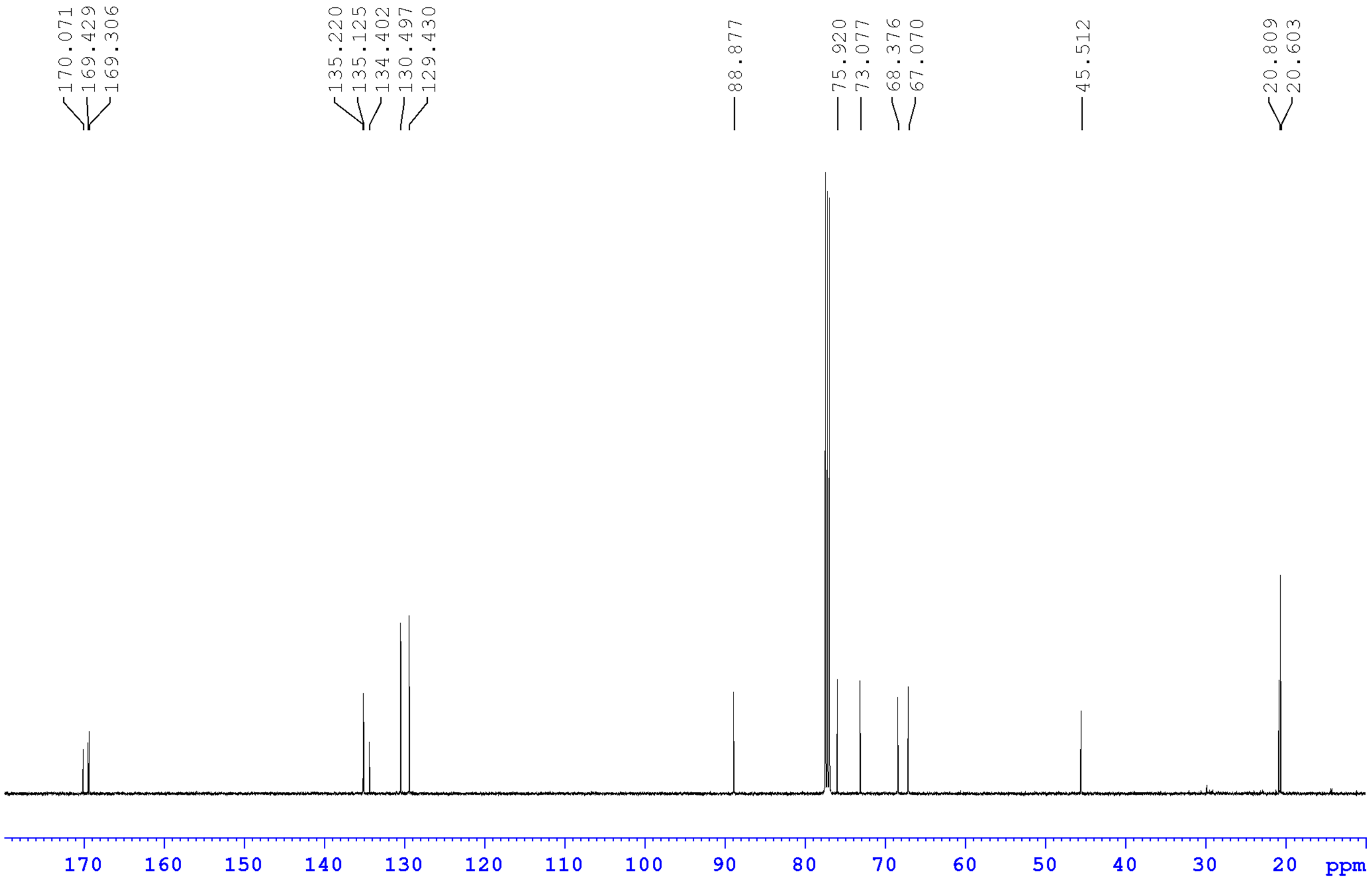

HRMS

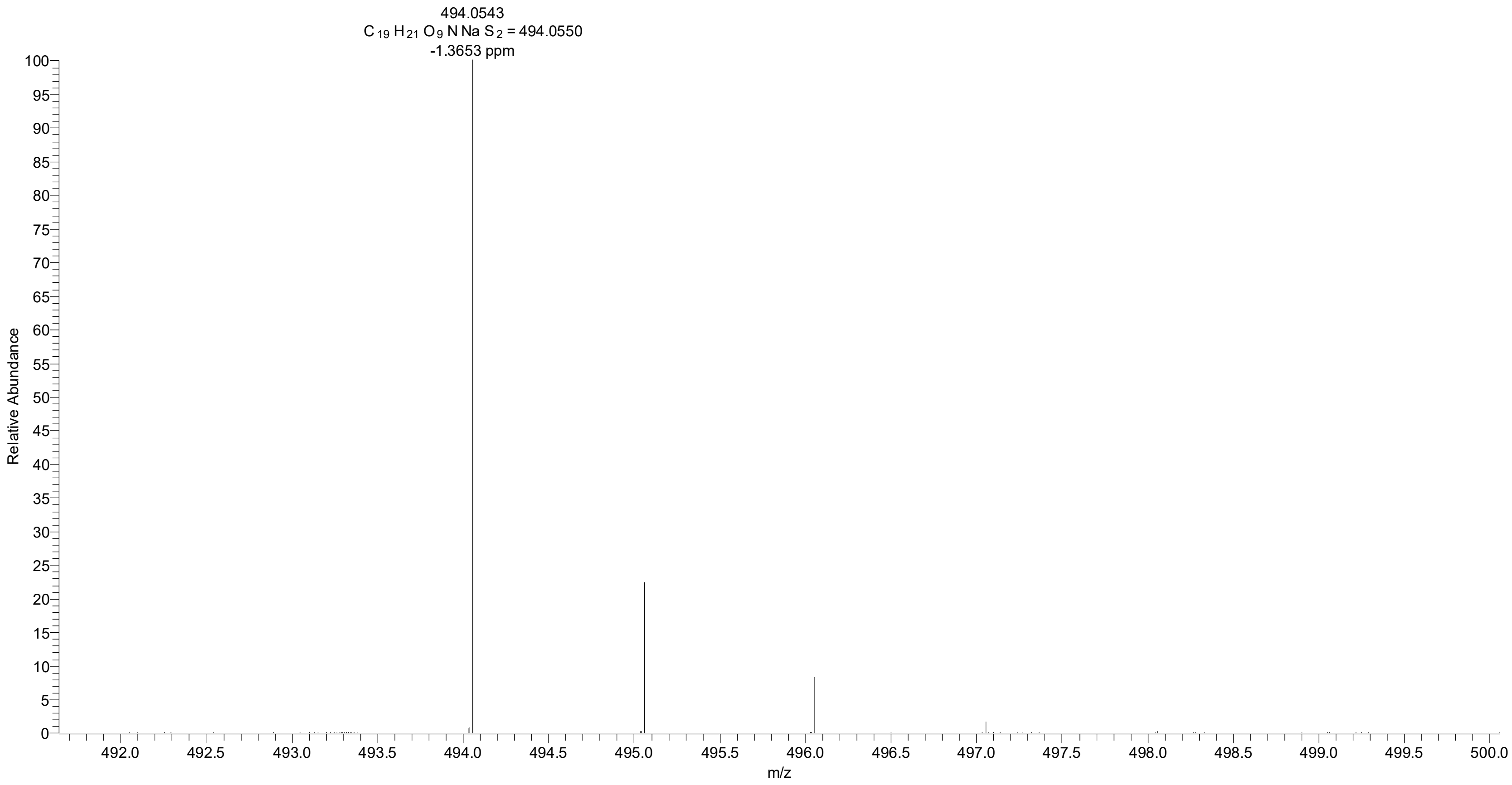

494.0543
C 19 H 21 O 9 N Na S 2 = 494.0550
-1.3653 ppm
Relative Abundance
m/z

Stability studies

To determine the compound's chemical stability, stability assays were performed. The compound was dissolved in a 4:1 mixture of $(CD_3)_2SO$ and D_2O. The resulting solution was monitored by 1H-NMR spectroscopy (300 MHz) at specific time intervals: 15 minutes, and 1, 2, 5, 10, and 24 hours. This procedure allowed for the real-time evaluation of the compound's stability by detecting any changes in the characteristic proton signals under the specified solvent conditions.

2,3,4-tri-*O*-acetyl-6-isothiocyanato-1,6-dideoxy-1-phenylsulfonyl-β-D-glucopyranose (**13**)

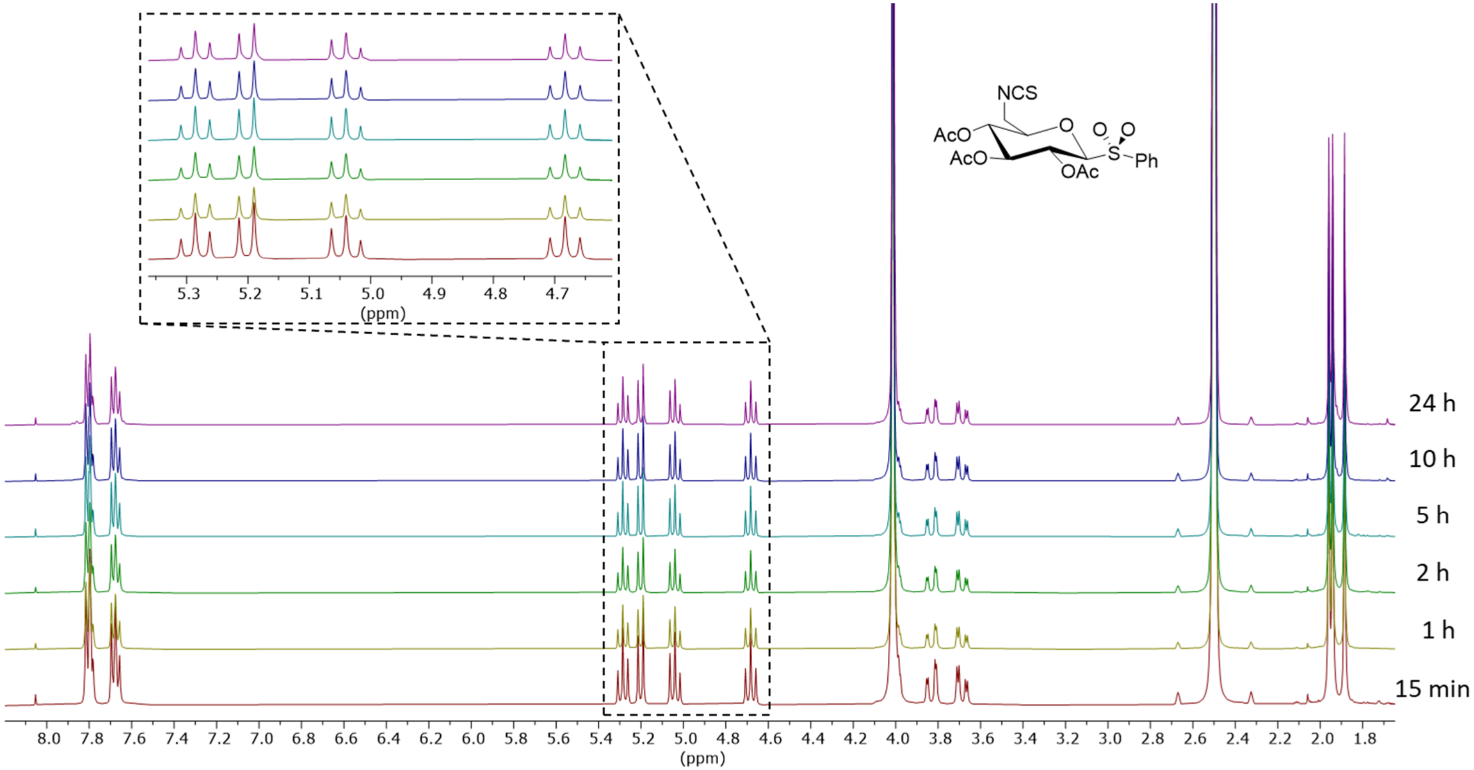

Figure S1. Time-dependent ^{1}H NMR spectra (300 MHz) of the compound **13** in $(CD_3)_2SO:D_2O$ 4:1 showing its stability profile at 15 min, 1 h, 2 h, 5 h, 10 h, and 24 h.

2,4,6-tri-*O*-acetyl-1-isothiocyanato-1,3-dideoxy-3-phenylsulfonyl-β-D-glucopyranoside, (**20**)

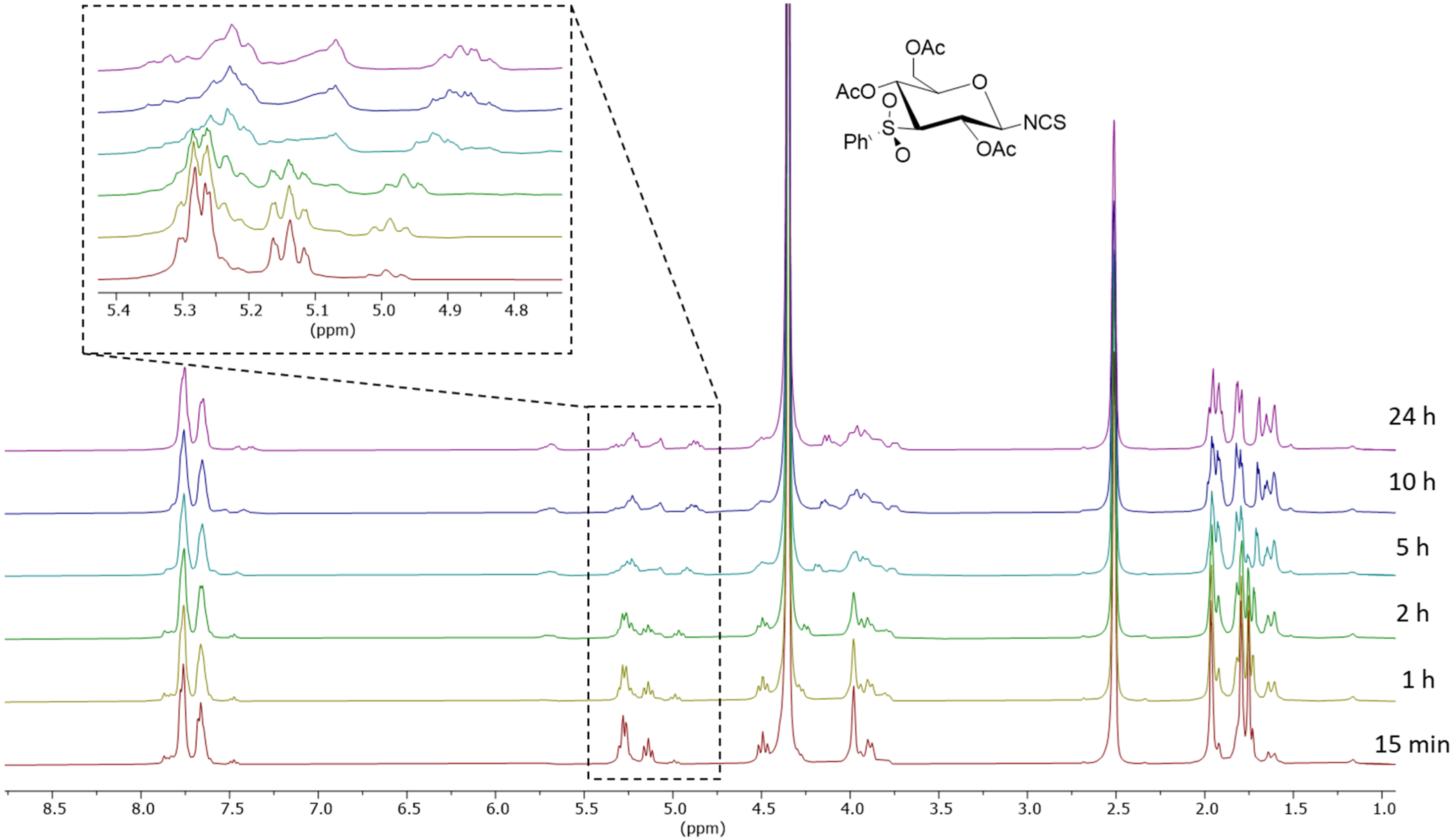

Figure S2. Time-dependent ^{1}H NMR spectra (300 MHz) of the compound **20** in $(CD_3)_2SO:D_2O$ 4:1 showing its stability profile at 15 min, 1 h, 2 h, 5 h, 10 h, and 24 h.

Cytotoxic activity against solid tumor cell lines

Cell viability assays and selectivity index calculations

Reagents and cell lines: Resazurin was procured from Sigma (St. Louis, MO, USA), while carboplatin was obtained from Teva (Teva Pharma S.L.U., Madrid, Spain). Cell culture reagents were sourced from Biowest (Nuaillé, France). Human keratinocytes (HaCaT), [4] human lung adenocarcinoma cells (A549), human melanoma cells (MeWo), and human bladder cancer cells (T24) were all acquired from the Cell Lines Service (CLS, Hamburg, Germany). Cell maintenance involved culturing in Dulbecco's Modified Eagle's Medium (DMEM) supplemented with 10% fetal bovine serum and 1% penicillin/streptomycin. All cell lines were cultured under standard conditions of 37 °C, 5% CO_2, and saturated humidity.

Assessment of cell viability: The resazurin assay, a widely utilized colorimetric/fluorescent technique, was employed to quantify cell viability. This method relies on the metabolic activity of cells to reduce the blue resazurin compound into a pink, soluble product, resorufin. The concentration of the generated resorufin is directly proportional to the number of viable cells. For the assay, 3,000 to 6,000 cells were seeded per well in 96-well plates and incubated for 24 hours to facilitate adherence. Subsequently, cells were exposed to varying concentrations of the tested compounds for 72 hours. Post-treatment, cells were rinsed once with phosphate-buffered saline (PBS), and 150 µL of resazurin (20 µg/mL in medium) was introduced into each well. Plates were then incubated for an additional 5 hours at 37 °C in a 5% CO_2 environment. Absorbance readings were recorded at 540 nm and 620 nm using a Multiskan EX Labsystems microplate spectrophotometer (Imark Bio Rad Laboratories Inc., Hercules, CA, USA). Results are expressed as a percentage of cell viability relative to untreated control cells. All reported data represent the mean ± standard error of the mean (SEM) from at least three independent experiments.

Statistical analysis and selectivity index determination: Statistical comparisons of cytotoxicity between HaCaT and T24 cell lines at specific compound concentrations were performed using a paired, two-tailed Student's t-test. A p-value exceeding 0.05 was considered statistically non-significant and is not explicitly marked. Statistical significance levels were defined as follows: $p < 0.05$ (#), $p < 0.01$ (##), and $p < 0.001$ (###). The selectivity index (S.I.) for each cancer cell line was determined by calculating the ratio of the non-malignant HaCaT cell line to the IC_{50} of the corresponding cancer cell line, based on the mean IC_{50} values derived from each independent experiment.

Graphical representations for the calculation of IC_{50} values

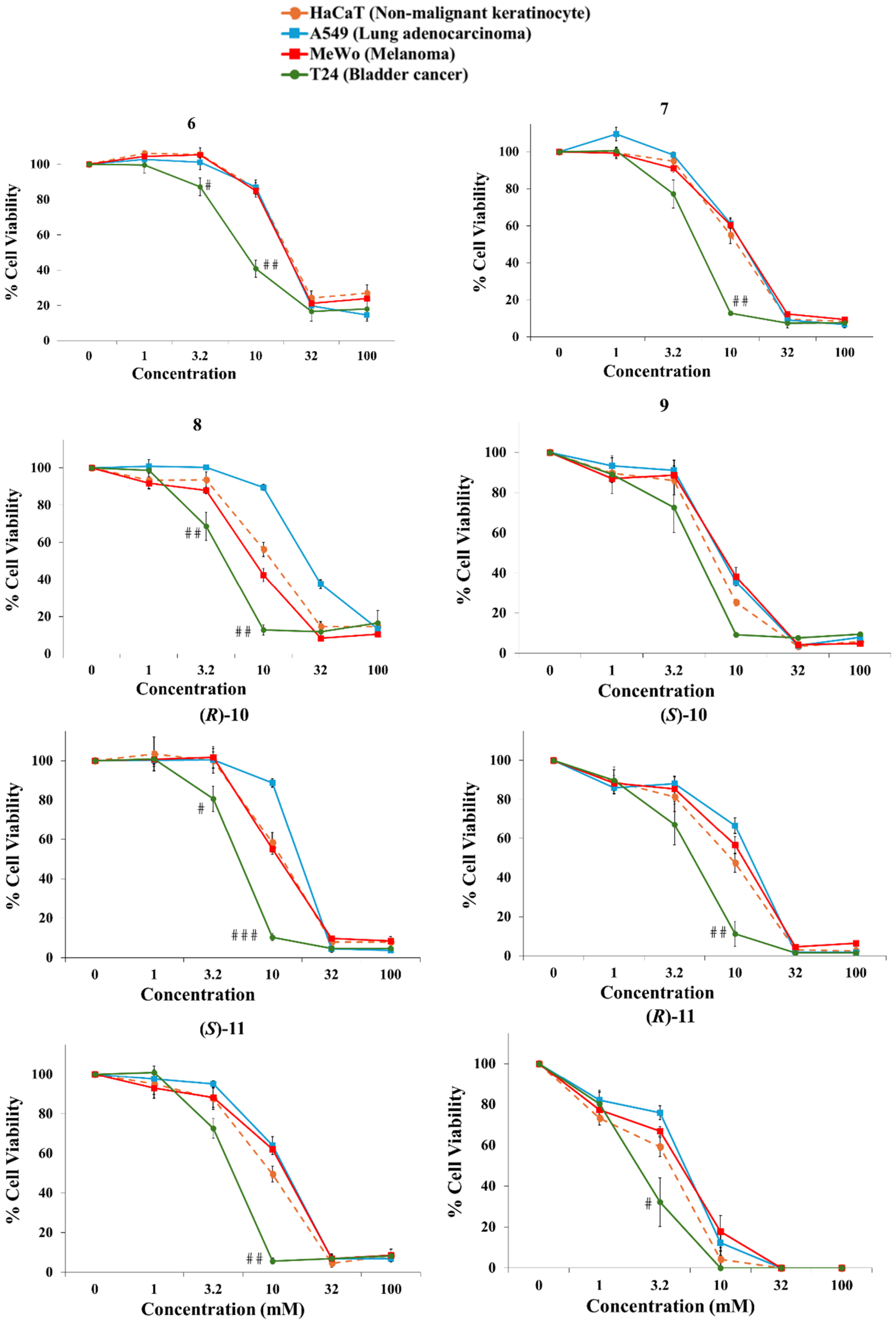

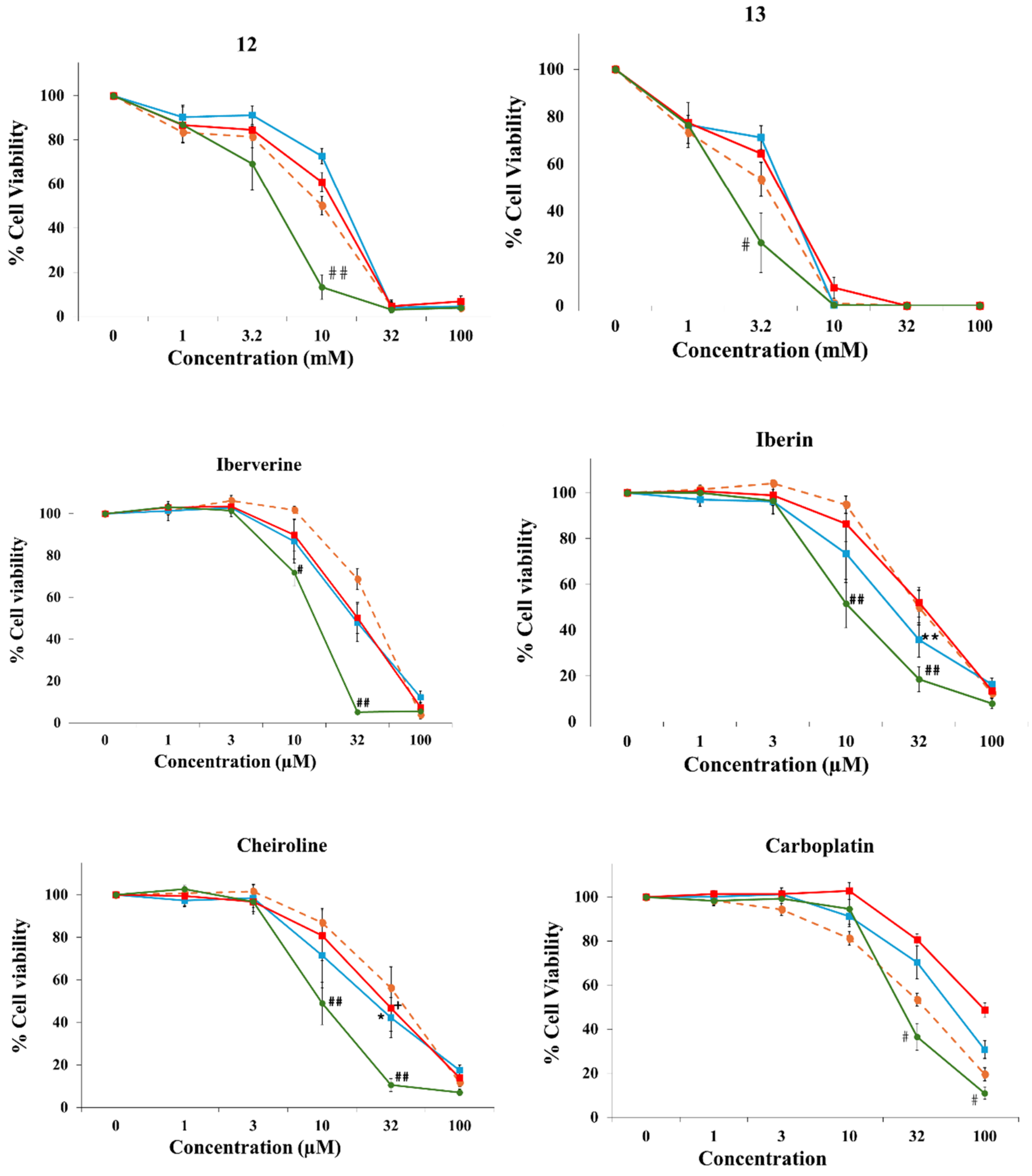

Figure S3. Graphical representations for the calculation of the IC_{50} values of 6-ITC *S*-glycosyl derivatives (compounds **6**–**13**), natural ITCs (iberverine, iberin and cheiroline), and carboplatin against solid tumor cell lines.

Cytotoxicity activity against leukemia cell lines of selected compounds

Graphical representations for the calculation of IC_{50} values

7

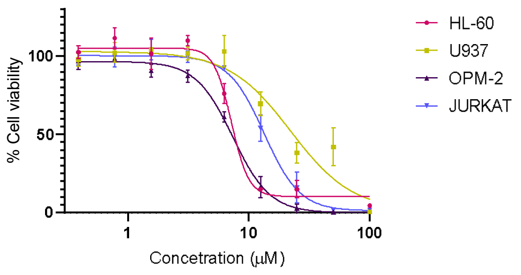

8

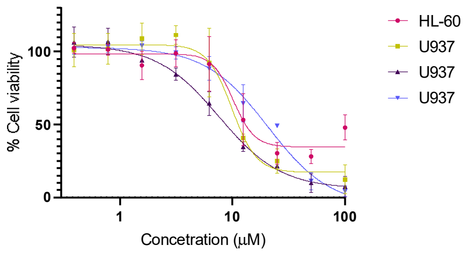

(*S*)-11

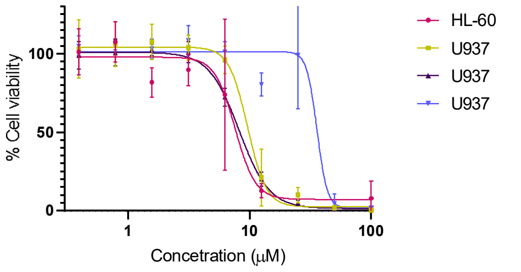

(*R*)-11

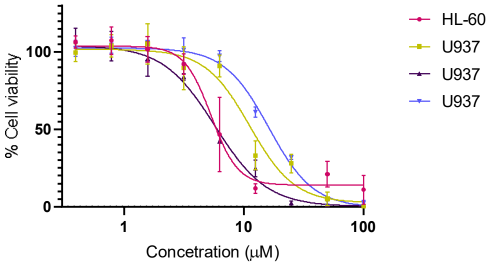

12

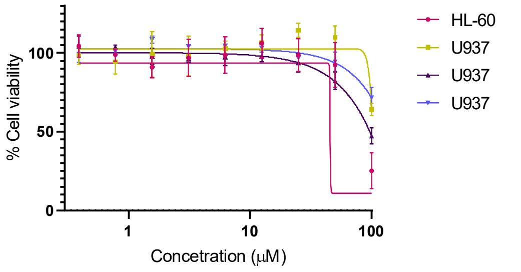

13

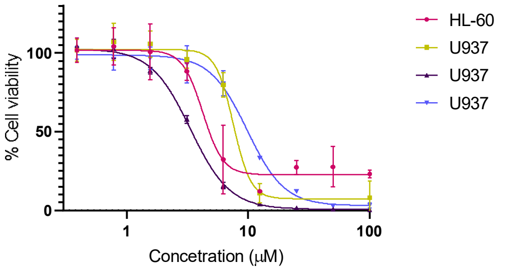

14

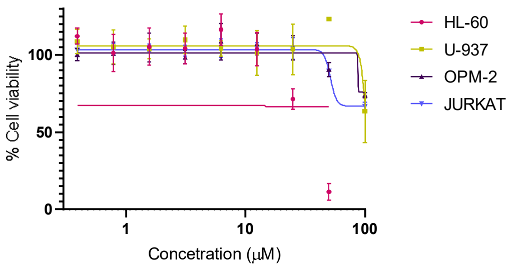

15

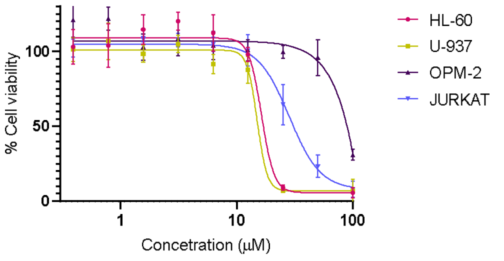

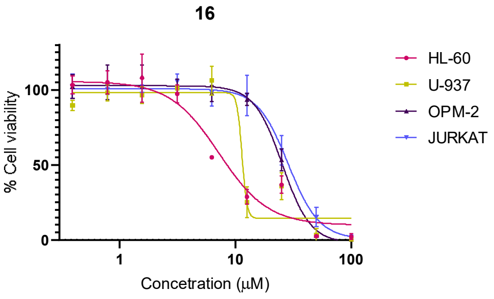
16
% Cell viability
100
50
0
1
10
100
Concetration (µM)
HL-60
U-937
OPM-2
JURKAT

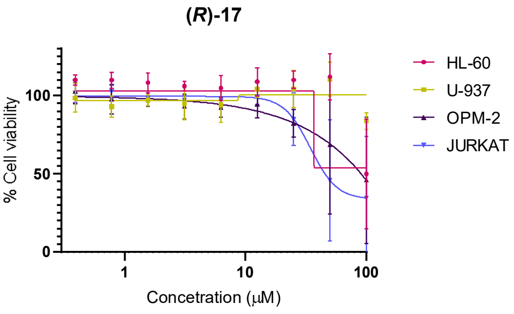
(R)-17
% Cell viability
100
50
0
1
10
100
Concetration (µM)
HL-60
U-937
OPM-2
JURKAT

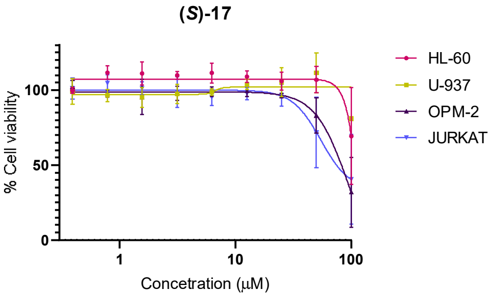
(S)-17
% Cell viability
100
50
0
1
10
100
Concetration (µM)
HL-60
U-937
OPM-2
JURKAT

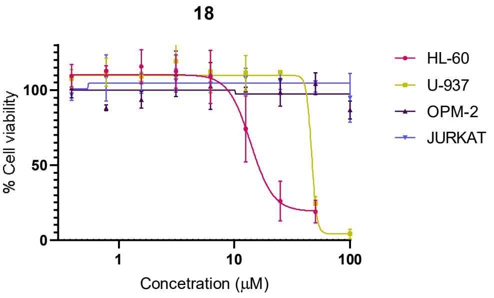
18
% Cell viability
100
50
0
1
10
100
Concetration (µM)
HL-60
U-937
OPM-2
JURKAT

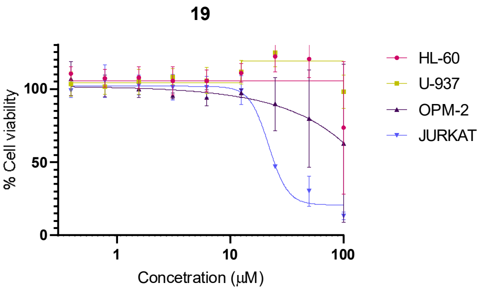
19
% Cell viability
100
50
0
1
10
100
Concetration (µM)
HL-60
U-937
OPM-2
JURKAT

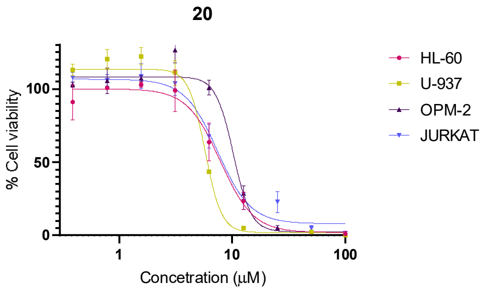
20
% Cell viability
100
50
0
1
10
100
Concetration (µM)
HL-60
U-937
OPM-2
JURKAT

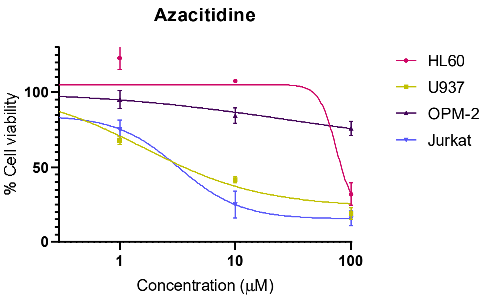
Azacitidine
% Cell viability
100
50
0
1
10
100
Concentration (µM)
HL60
U937
OPM-2
Jurkat

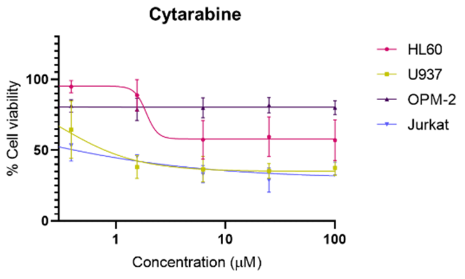
Cytarabine
% Cell viability
100
50
0
1
10
100
Concentration (µM)
HL60
U937
OPM-2
Jurkat

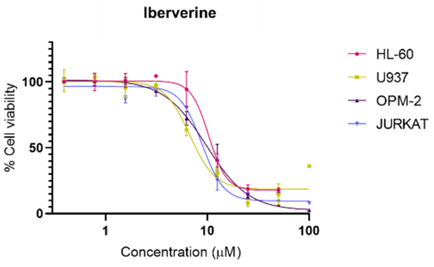
Iberverine
% Cell viability
100
50
0
1
10
100
Concentration (µM)
HL-60
U937
OPM-2
JURKAT

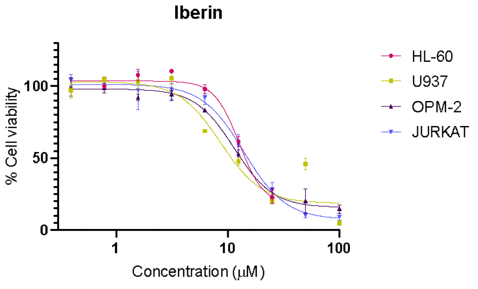
Iberin
% Cell viability
100
50
0
1
10
100
Concentration (µM)
HL-60
U937
OPM-2
JURKAT

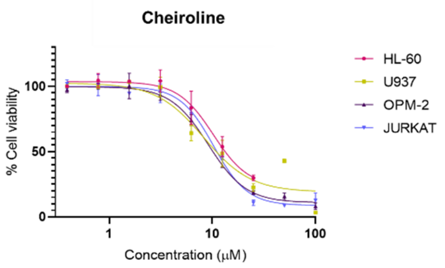

Figure S4. Graphical representations for the calculation of the IC_{50} values of glucose-derived β-*N*-glycosyl (**7**, **8** and **11**–**13**), β-*S*-glycosyl (**14**–**20**), natural isothiocyanates (iberverine, iberin, and chieroline), and reference antileukemic drugs, azacitidine and cytarabine, against leukemia cell lines.

Cell population distribution by FACS with 7AAD/Annexin V staining

HL-60

13 (0 μM) **13** (6 μM) **13** (50 μM)

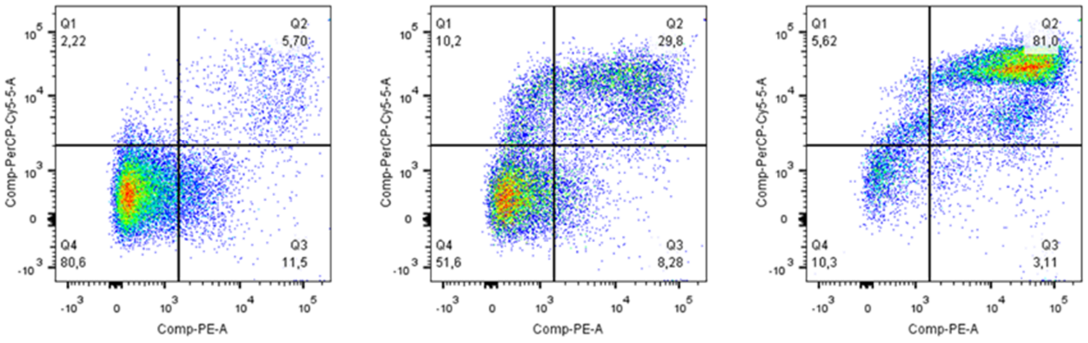

HL-60

20 (0 μM) **20** (6 μM) **20** (50 μM)

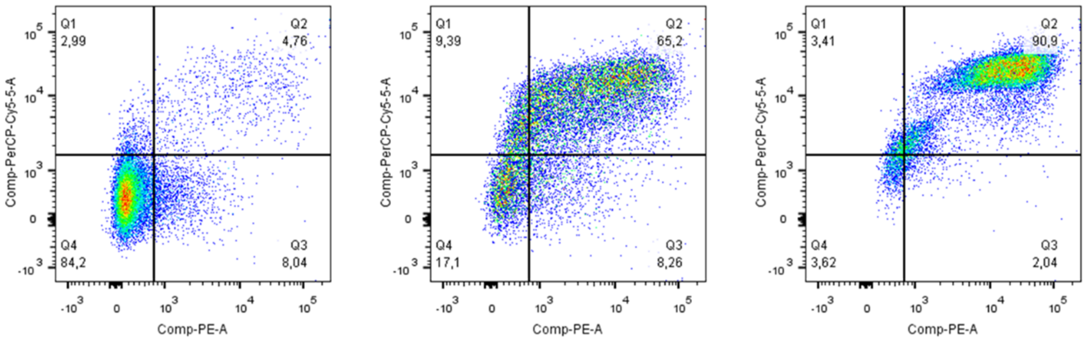

U937

13 (0 µM)

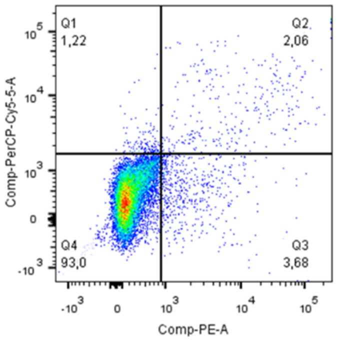

13 (6 µM)

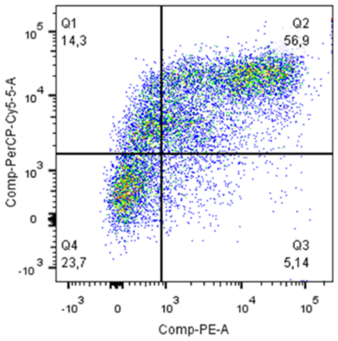

13 (50 µM)

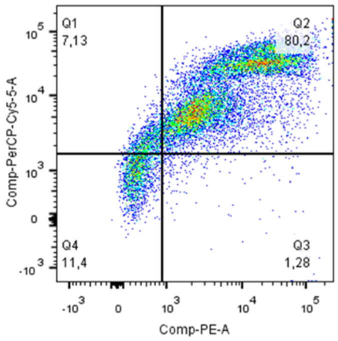

U937

20 (0 µM)

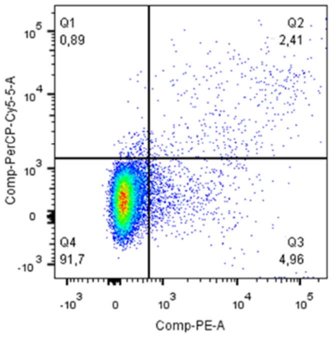

20 (6 µM)

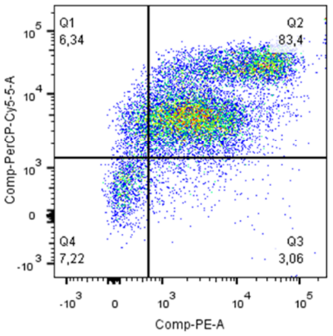

20 (50 µM)

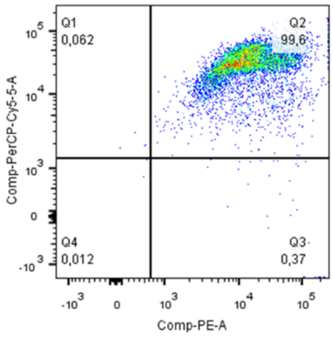

OPM-2

13 (0 µM)

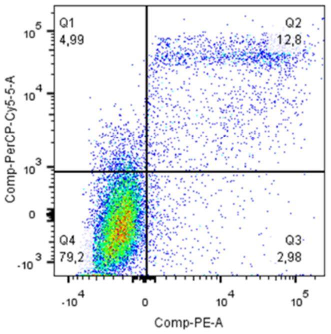

13 (6 µM)

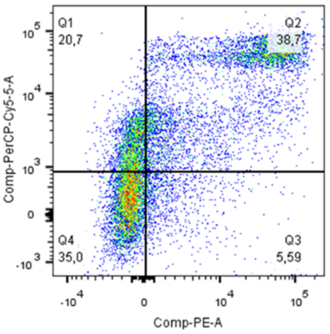

13 (50 µM)

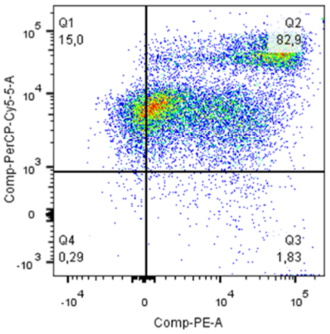

OPM-2

20 (0 µM)

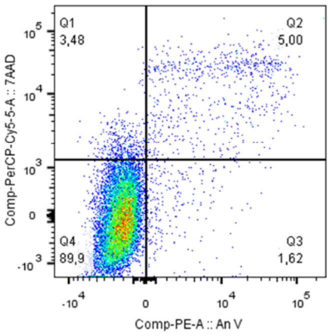

20 (6 µM)

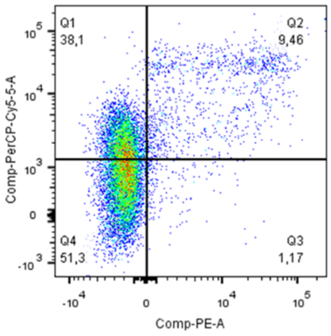

20 (50 µM)

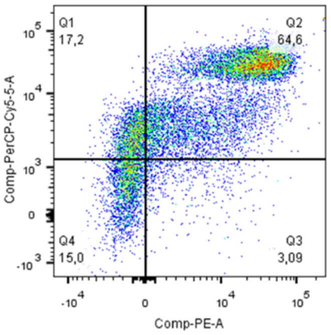

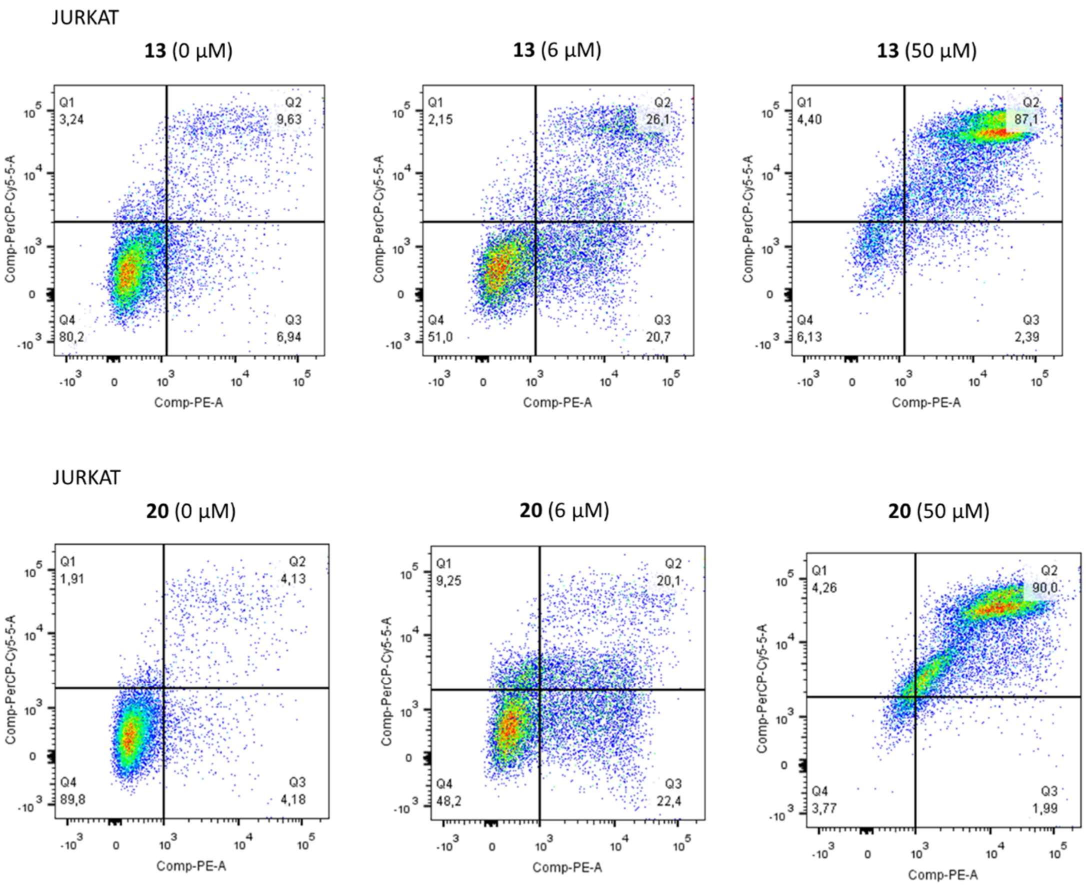

Figure S5. Cell viability analysis of HL60, U937, JURKAT, and OPM-2 cell lines. Cells were analyzed after 18 h incubation with vehicle or compounds 13 and 20 (6 µM and 50 µM doses). Apoptosis and viability were assessed by Annexin V/7AAD staining using FACS analysis.

Antioxidant activity (Nrf2 induction activity)

For evaluating Nrf2 induction, AREc32 cells, a MCF7 cell line stably modified with the pGL-8xARE plasmid, were used. This plasmid incorporates eight copies of luciferase reporter gen after the antioxidant response element (ARE) sequences. This design allows for the measurement of Nrf2 activation by quantifying the luminescence produced through luciferase activity.

The AREc32 cells, a generous contribution from CR Wolf, were cultivated in Dulbecco's modified Eagle's medium (DMEM) with GlutaMAX (Gibco, Invitrogen, Spain). This medium was further supplemented with 10% (v/v) filtered fetal bovine serum (FBS; Gibco, Invitrogen, Spain), 1% penicillin/streptomycin antibiotics, and geneticin (0.8 mg/mL G418; Gibco, Invitrogen, Spain).

For Nrf2 induction experiments, 20,000 cells per well were plated into 96-well white flat-bottom plates. After allowing 24 hours for cell attachment, selected compounds were applied to the cells at various concentrations in duplicate for another 24 hours. Subsequently, the Luciferase assay system (Promega E1500) was used according to the manufacturer's guidelines, and luminescence was read on a Clariostar multiwell plate reader (BMG Labtech Germany).

Luciferase activity was normalized towards basal conditions. Data were expressed as CD values, which represent the concentration required to double the luciferase activity compared to the baseline. These CD values were calculated from dose-response curves, which were fitted via non-linear regression for each compound after logarithmically transforming the data using GraphPad Prism 8.0 software.

To determine EC_{50} values, an MTT assay was performed. Cells were seeded at a density of 20,000 cells per well in 96-well transparent plates and incubated for 24 hours. Following this, cells were treated with the corresponding compounds at increasing concentrations. After the incubation period, cell viability was assessed using 3-(4,5-dimethylthiazol-2-yl)-2,5-diphenyltetrazolium bromide (MTT, 0.5 mg/mL).

In this essay, the yellow MTT salt is reduced by oxidoreductase enzymes in viable cells, forming insoluble purple formazan crystals. These formazan crystals were then solubilized by adding dimethyl sulfoxide (DMSO) to the plates. Finally, absorbance was measured at 535 nm using a Clariostar Nano microplate reader (BMG Labtech, Germany). Basal absorbance was set to 100%, and all results were normalized to these basal conditions. Across all cases, the calculated cell viability, expressed as EC_{50}, was consistently found to be higher than 30 µM.

Solubility values and Lipinski rules of 6-ITC glucose-based derivatives, 6–13. [5]

Table S1. Solubility parameters and Lipinski's rule-of-five properties of 6-ITC glucose-based derivatives. The data summarize the predicted solubility profiles and key physicochemical parameters relevant to oral bioavailability, including molecular weight, hydrogen bond acceptors and donors, logP values, and compliance with Lipinski's guidelines.

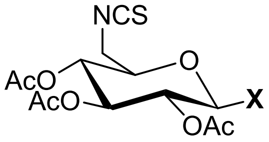

X = SOnR (**6**-**13**)
n = 0, 1, 2

X	Compounds	ESOL Log S	ESOL Solubility (mg/ml) [6]	ESOL Class	MW	H-bond acceptors	H-bond donors	MLogP	Lipinski violations [7]
MeS	**6**	-2.83	5.62e-01	Soluble	377.43	8	0	1.16	0
EtS	**7**	-3.08	3.25e-01	Soluble	391.46	8	0	1.42	0
PhS	**8**	-4.28	2.30e-02	Moderately soluble	439.50	8	0	2.41	0
PhS	**9**	-4.28	2.30e-02	Moderately soluble	439.50	8	0	2.41	0
(*R*)-EtSO	**(*R*)-10**	-2.14	2.95e+00	Soluble	407.46	9	0	0.56	0
(*S*)-EtSO	**(*S*)-10**	-2.14	2.95e+00	Soluble	407.46	9	0	0.56	0
(*S*)-PhSO	**(*S*)-11**	-3.44	1.66e-01	Soluble	455.50	9	0	1.55	0
(*R*)-PhSO	**(*R*)-11**	-3.44	1.66e-01	Soluble	455.50	9	0	1.55	0
$EtSO_2$	**12**	-2.47	1.42e+00	Soluble	423.46	10	0	0.52	0
$PhSO_2$	**13**	-3.67	1.01e-01	Soluble	471.50	10	0	1.52	0
-	**Iberverine**	-2.81	5.87E-01	Soluble	377.4	8	0	1.97	0
-	**Iberin**	-3.06	3.39E-01	Soluble	391.5	8	0	2.23	0
-	**Cheiroline**	-4.26	2.40E-02	Moderately soluble	439.5	8	0	3.22	0
-	**TBHQ**	-2.96	1.83E-01	Soluble	166.2	2	2	2.1	0
-	**Azacitidine**	-0.12	1.87e+02	Very soluble	244.20	7	4	-2.98	0
-	**Cytarabine**	-0.14	1.78e+02	Very soluble	243.22	6	4	-2.29	0

Parameter	
**Solubility scale (Log S)**	Insoluble < -10 < Poorly < -6 < Moderately < -4 < Soluble < -2 < Very < 0 < Highly
**Lipinski violations**	Based on Lipinski rules:<br><br>MW ≤ 500<br>MLogP ≤ 4.15<br>N or O ≤ 10<br>NH or OH ≤ 5

Oral viability:

<table>
<tr><th>6</th><th>7</th><th>8</th></tr>
<tr>
<td>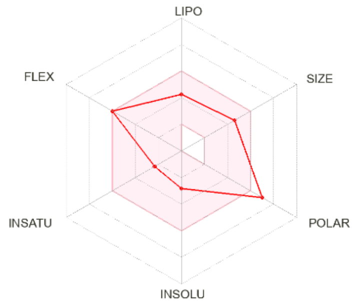
</td>
<td>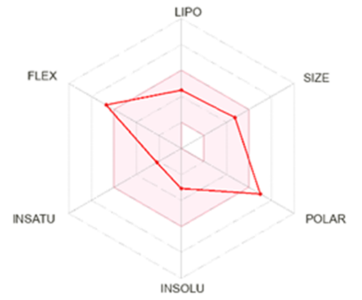
</td>
<td>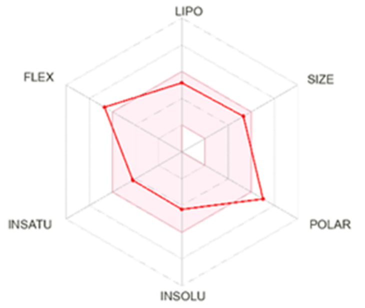
</td>
</tr>
<tr><th>9</th><th>(R)-10</th><th>(S)-10</th></tr>
<tr>
<td>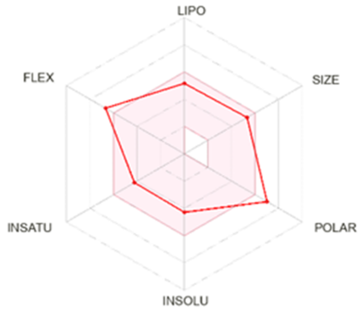
</td>
<td>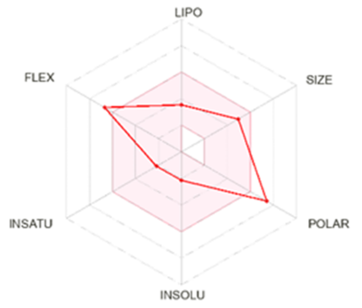
</td>
<td>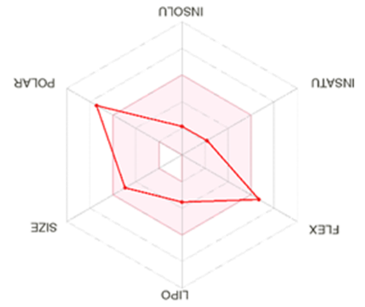
</td>
</tr>
<tr><th>(S)-11</th><th>(R)-11</th><th>12</th></tr>
<tr>
<td>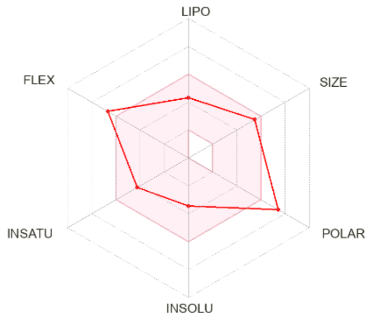
</td>
<td>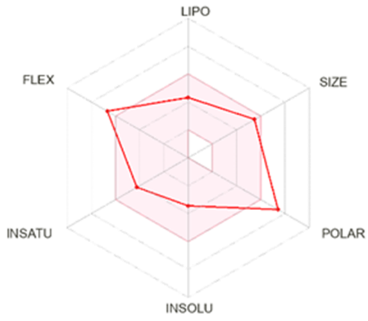
</td>
<td>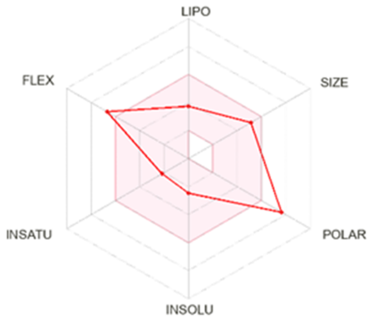
</td>
</tr>
</table>

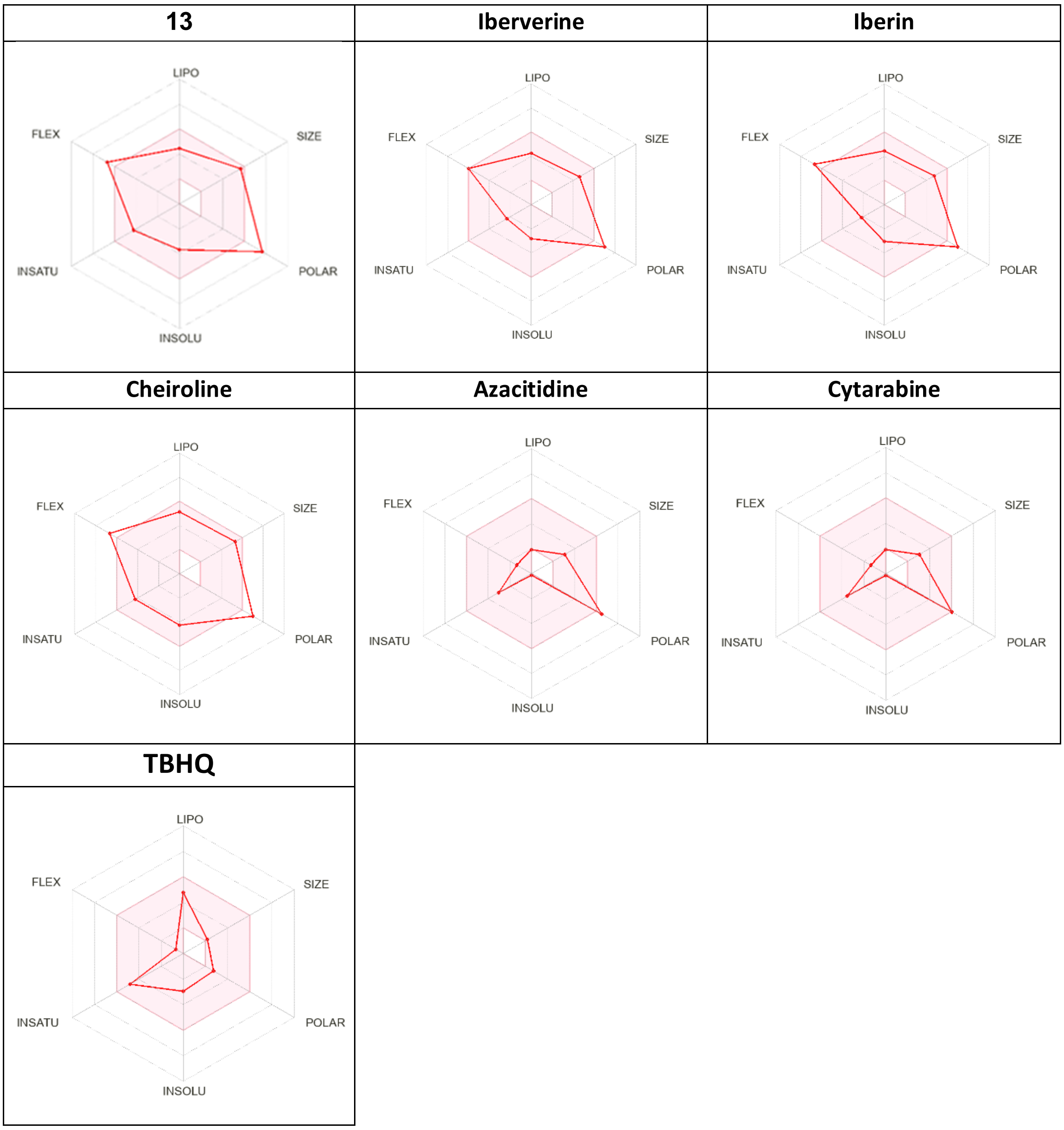

Figure S6. Radar chart representation of the oral drug-likeness profile of 6-ITC glucose-based derivatives (**6**–**13**), natural isothiocyanates (iberverine, iberin, and chieroline) and reference drugs (azacitidine, cytarabine, and *tert*-Butylhydroquinone). Parameters include lipophilicity (LIPO), size (SIZE), polarity (POLAR), solubility (INSOLU), flexibility (FLEX), and saturation (INSATU).

In Silico Docking to STAT3 SH2 Domain

An initial in silico investigation was performed to evaluate the potential interactions of the novel S-glycoside regioisomers at the SH2 domain of STAT3, following methodologies previously applied to the N-glycoside series [8] and other ITCs [9]. Docking analyses were subsequently performed to compare the binding behavior of the new *S*-glycosides 6–13 with that previously reported for their N-glycoside counterparts 14–20.

This computational analysis does not provide experimental evidence of direct target engagement or modulation of any specific protein, but suggested that the new *S*-glycoside regioisomers may engage the same three binding pockets previously identified for the *N*-glycoside derivatives [8], with the SH2 domain pocket 1 showing the most favorable binding energies and interaction profiles, Figure 7. Only the most favorable interaction energy values at each pocket (1–3), identified within the SH2 domain of STAT3, are reported in the Table 1. These results provided the basis for a detailed analysis of the residue-level contributions and the comparative binding behavior of representative compounds within this pocket.

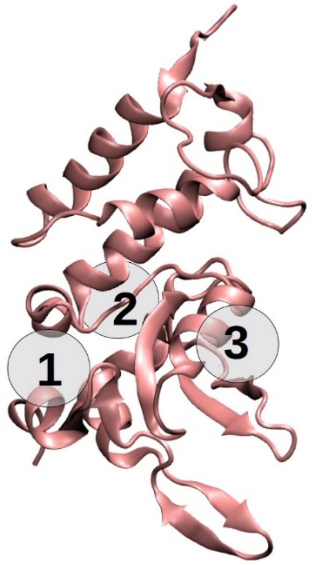

Figure S7: Schematic representation of the SH2 domain of the STAT3 protein highlighting the three predicted binding pockets within the domain structure.

Compounds bearing phenyl substituents at sulfur were predicted to form stronger interactions across all pockets compared to their ethyl-substituted analogues. For instance, ethyl sulfoxide (*S*)-10 (R=Et) showed weaker binding than the phenylsulfinyl derivative (*S*)-11 (R=Ph) within binding pocket 1 (Table 1). Additionally, the sulfur oxidation state appeared to directly influence binding stability, increasing its interaction efficiency: at pocket 3, the ethylthioether 7 (R=Et) showed a binding free energy of −1.95 kcal/mol, compared to the ethylsulfoxide (*R*)-10 (R=Et), −10.36 kcal/mol, and the ethylsulfone 12 (R=Et), −12.34 kcal/mol (Table 1). A similar trend was observed for phenyl substituents: at binding pocket 2, the phenylthioether 8 (R=Ph) displayed −14.23 kcal/mol, whereas the phenylsulfoxide (*R*)-11 (R=Ph) reached −15.80 kcal/mol; and at binding pocket 1, phenylsulfoxide (*S*)-11 (R= Ph) showed −33.30 kcal/mol compared to the phenylsulfone 13 (R=Ph), −40.28 kcal/mol (Table 1). Overall, *S*-configured sulfur epimers generally exhibited more favorable binding free energies and stronger interactions across the

studied pockets, suggesting that sulfur stereochemistry plays a determinant role in stabilizing the predicted ligand–STAT3 complexes.

Table S2. Binding free energy between the β-*S*-glycoside derivatives **7**–**13** and the most favorable binding pocket (1–3) of STAT3.

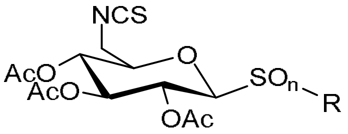

Entry	n	R	Compound	Binding Pocket	Energy[a] (kcal/mol)
1	2	Ph	**13**	1	-40.28
2	1	Ph	**(*S*)-11**	1	-33.30
3	1	Et	**(*S*)-10**	1	-23.95
4	1	Ph	**(*R*)-11**	2	-15.80
5	0	Ph	**8**	2	-14.23
6	2	Et	**12**	3	-12.34
7	1	Et	**(*R*)-10**	3	-10.36
8	0	Et	**7**	3	-1.95

[a]Applying the MMPBSA algorithm of the molecular dynamics simulations.

Among the evaluated compounds of both series, N- and S-glycosides, phenylsulfones 13 and 20 [8], displayed the most favorable binding energies within pocket 1 of the STAT3 SH2 domain, Table 2.

Table S3: Binding free energy between each iberin derivative and the main amino acids that characterize each binding pocket.

**Binding Pocket 1**					
**Residue**	**Energy (kcal/mol)**	**Residue**	**Energy (kcal/mol)**	**Residue**	**Energy (kcal/mol)**
**(*S*)-10**		**(*S*)-11**		**13**	
ARG27	-6.54	ARG27	-6.48	ARG27	-6.95
SER30	-1.65	SER30	-1.10	SER30	-1.09
GLU32	-1.13	GLN33	-2.27	GLN33	-2.17
GLN33	-1.95	LEU88	-0.23	TRP89	-2.04
TRP89	-2.35	TRP89	-2.41	GLY92	-4.09
GLY92	-1.91	GLU91	-2.18	TYR93	-6.77
TYR93	-1.41	GLY92	-3.49	ILE94	-2.11
ILE94	-2.94	TYR93	-6.93	MET95	-5.97
MET95	-6.68	ILE94	-2.08	GLY96	-0.76

GLY96	-0.23	MET95	-3.95	LEU116	-0.24
LEU116	-0.84	GLY96	-0.13	LEU117	-1.02
LEU117	-0.67	LEU117	-0.96	LEU182	-3.46
LEU182	-1.19	LEU182	-2.65	TYR183	-2.62
TYR183	-2.87	TYR183	-1.86	PRO184	-2.75
PRO184	-2.44	PRO184	-2.42	ARG27	-6.95
**Binding Pocket 2**					
**8**		**(*R*)-11**			
TYR84	-1.89	LYS83	-3.54		
ILE85	-0.95	TYR84	-5.35		
LEU86	-1.33	ILE85	-2.26		
ALA87	-1.65	LEU86	-4.12		
LEU88	-1.19	ALA87	-2.48		
SER158	-1.28	LEU154	-0.70		
ALA160	-0.64	ASN155	-1.03		
TYR195	-4.65	SER158	-2.81		
CYS196	-0.55				
**Binding Pocket 3**					
**7**		**(*R*)-10**		**12**	
LIS100	-1.62	THR9	-0.87	SER23	-0.25
GLU103	-0.74	TRP10	-5.57	THR24	-0.81
ARG104	-1.89	ASP11	-3.65	THR25	-0.84
THR135	-0.30	ALA14	-0.37	LYS26	-0.37
ILE143	-0.68	TYR48	-1.11	ILE143	-2.79
GLN144	-0.60	GLN142	-0.33	GLN144	-1.50
SER145	-0.94	ILE143	-0.21	SER145	-0.98
		GLN144	-0.43		
		SER145	-0.23		

In both cases, the complexes were mainly stabilized by Van der Waals and dispersion interactions, with a minor although relevant electrostatic contribution, Figure 8.

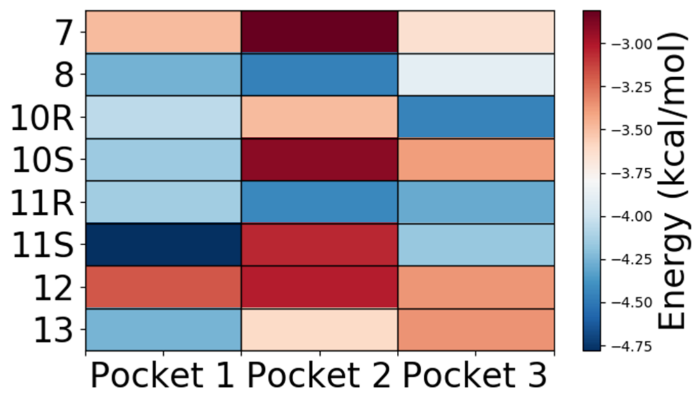

Figure S8: Color map of the binding free energies obtained for the interaction STAT3-ligand in each of the determined pockets from a previous study [8].

Considering compound **13**, the residues ARG27, GLY92, TYR93, MET95, and LEU182 contributed most significantly to binding stabilization, reflecting a deep accommodation of the ligand within the hydrophobic cavity. The guanidinium group of ARG27 established a strong electrostatic interaction with a polar oxygen atom of the ligand, whereas TYR93, MET95, and LEU182 reinforced binding through hydrophobic and dispersive contacts. In contrast, compound 20 showed a slightly different orientation within the same pocket, with predominant interactions involving SER30, GLU32, MET95, and TYR183. The displacement of the phenylsulfone group toward the more polar SER30–GLU32 region weakened its electrostatic complementarity with ARG27, leading to a moderate reduction in binding affinity relative to compound **13**. Overall, the docking results suggest a possible interaction with STAT3 that may contribute to the observed biological profile, and highlight the importance of the ligand orientation and substituent positioning in optimizing interactions within pocket 1 of STAT3. However, these findings should be regarded as exploratory and require experimental validation.

Taking together, these computational results provide a consistent structural rationale for the potential biological activity of the new *S*-glycoside ITCs derivatives. The strong stabilization of compound **13** within STAT3 SH2 pocket 1 could justify its selection for biological evaluation, should STAT3 prove to be a relevant target in future mechanistic studies. The comparative analysis with compound **20** further underscores the importance of sulfone orientation and the preservation of key interactions with ARG27 in achieving high binding affinity.

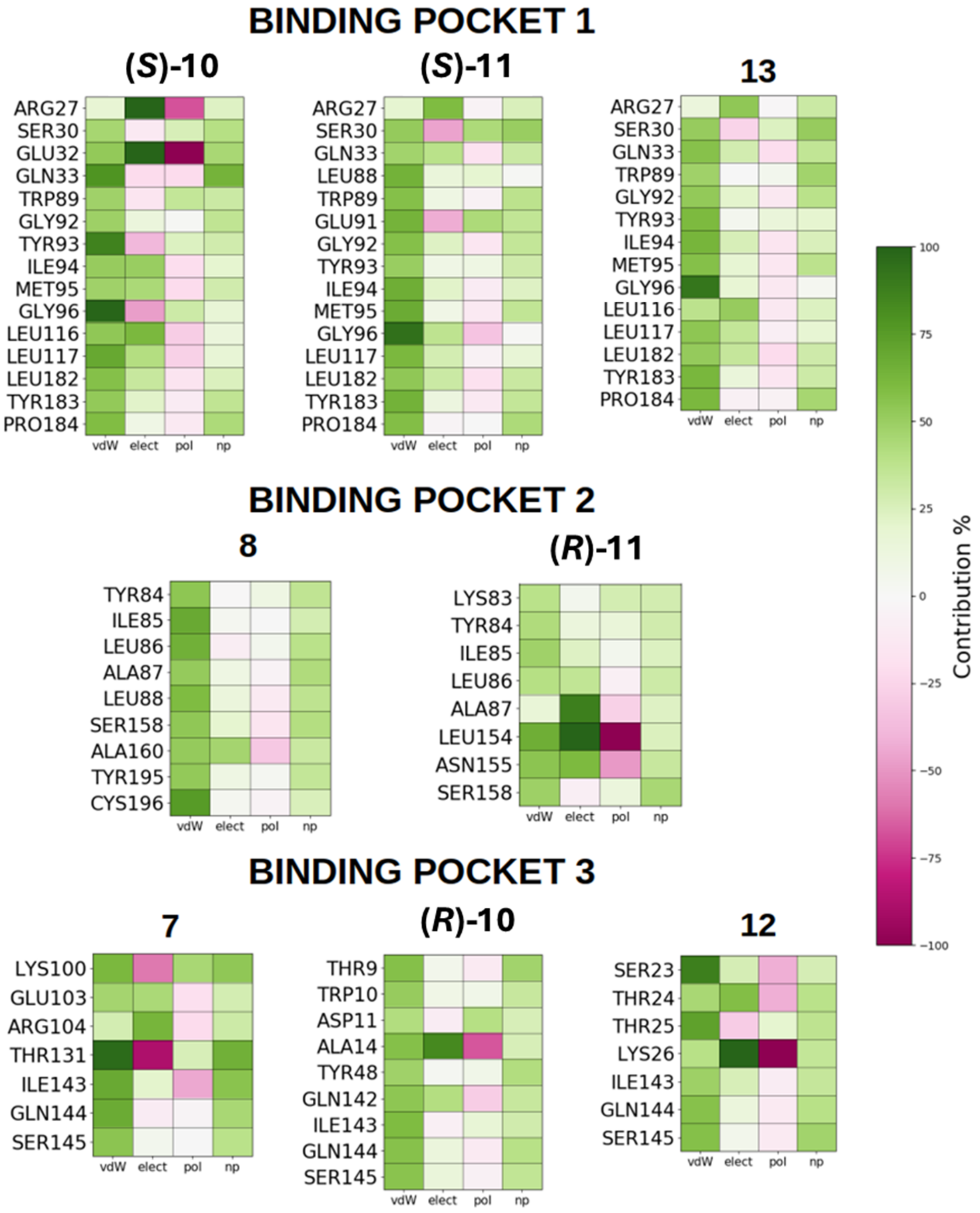

Figure S9: Pairwise residue decomposition of the binding free energy for every compound with the closest amino acids of the SH2 domain. The values are relative to the total binding free energy (% form): green and purple account for positive and negative contributions to the pairwise binding free energy. Total binding free energy is decomposed in four different contributions: van der Waals (vdW), electrostatic (elect), polar solvation (pol), and non-polar solvation (np).

Bibliography

[1] N. Darabedian, J. Gao, K.N. Chuh, C.M. Woo, M.R. Pratt, The Metabolic Chemical Reporter 6-Azido-6-deoxy-glucose Further Reveals the Substrate Promiscuity of *O*-GlcNAc Transferase and Catalyzes the Discovery of Intracellular Protein Modification by *O*-Glucose, J. Am. Chem. Soc. 140 (2018) 7092–7100. https://doi.org/10.1021/jacs.7b13488.

[2] K. Günther, C. Schips, T. Ziegler, Preparation of Some Glycosyl Amino Acid Building Blocks via Click Reaction and Construction of a Glycotetrapeptide Library Using Spot Synthesis, Journal of Carbohydrate Chemistry 27 (2008) 446–463. https://doi.org/10.1080/07328300802419873.

[3] S. Mehta, M. Meldal, V. Ferro, J.Ø. Duus, K. Bock, Internally quenched fluorogenic, α-helical dimeric peptides and glycopeptides for the evaluation of the effect of glycosylation on the conformation of peptides, J. Chem. Soc., Perkin Trans. 1 (1997) 1365–1374. https://doi.org/10.1039/a607624f.

[4] P. Boukamp, R.T. Petrussevska, D. Breitkreutz, J. Hornung, A. Markham, N.E. Fusenig, Normal keratinization in a spontaneously immortalized aneuploid human keratinocyte cell line., The Journal of Cell Biology 106 (1988) 761–771. https://doi.org/10.1083/jcb.106.3.761.

[5] A. Daina, O. Michielin, V. Zoete, SwissADME: a free web tool to evaluate pharmacokinetics, drug-likeness and medicinal chemistry friendliness of small molecules, Sci Rep 7 (2017) 42717. https://doi.org/10.1038/srep42717.

[6] J.S. Delaney, ESOL: Estimating Aqueous Solubility Directly from Molecular Structure, J. Chem. Inf. Comput. Sci. 44 (2004) 1000–1005. https://doi.org/10.1021/ci034243x.

[7] C.A. Lipinski, F. Lombardo, B.W. Dominy, P.J. Feeney, Experimental and computational approaches to estimate solubility and permeability in drug discovery and development settings, Advanced Drug Delivery Reviews 46 (2001) 3–26. https://doi.org/10.1016/S0169-409X(00)00129-0.

[8] L.A. Prieto, N. Khiar-Fernández, J.M. Calderón-Montaño, M. López-Lázaro, J. Lucía-Tamudo, J.J. Nogueira, R. León, N. Moreno, V. Valdivia, R. Recio, I. Fernández, Exploring the broad-spectrum activity of carbohydrate-based Iberin analogues: From anticancer effect to antioxidant properties, European Journal of Medicinal Chemistry 289 (2025) 117469. https://doi.org/10.1016/j.ejmech.2025.117469.

[9] T. Rajakumar, P. Pugalendhi, Allyl isothiocyanate inhibits invasion and angiogenesis in breast cancer via EGFR-mediated JAK-1/STAT-3 signaling pathway, Amino Acids 55 (2023) 981–992. https://doi.org/10.1007/s00726-023-03285-2.
